# A Systematic Review on the Clinical Pharmacokinetics of Cephalexin in Healthy and Diseased Populations

**DOI:** 10.3390/antibiotics12091402

**Published:** 2023-09-03

**Authors:** Mohammed Kanan, Shahd Atif, Faisal Mohammed, Yara Balahmar, Yasir Adawi, Revan AlSaleem, Ahmed Farhan, Manayer Alghoribi, Saud Mohammed, Raghad Alshanbari, Malak Fahad, Rana Kallab, Reem Mohammed, Dimah Alassaf, Ashwag Hazza

**Affiliations:** 1 Department of Clinical Pharmacy, King Fahad Medical City, Riyadh 12211, Saudi Arabia; 2 Al Iman General Hospital, Riyadh 12211, Saudi Arabia; shahad.atif1@gmail.com; 3 Department of Clinical Pharmacy, College of Pharmacy, Umm Al-Qura University, Makkah 24211, Saudi Arabia; faisalpo1029@gmail.com; 4 Department of Clinical Pharmacy, College of Pharmacy, Ibn Sina College, Jeddah, Saudi Arabia; yarabalahmar@hotmail.com; 5 Department of Clinical Pharmacy, Jazan University, Jazan 85534, Saudi Arabia; yasir50adawi@gmail.com; 6 College of Pharmacy, King Khalid University, Abha 61421, Saudi Arabia; refanq21@gmail.com (R.A.); sauod1128@gmail.com (S.M.); reemalshehri.m@gmail.com (R.M.); 7 Department of Pharmacy, Prince Sultan Military Medical City, Riyadh 12211, Saudi Arabia; ahmedaldafiri9@gmail.com; 8 Al-Nahda General Hospital, Private Healthcare, Taif 26575, Saudi Arabia; manayer.g@hotmail.com; 9 Department of Pharmacy, Erfan and Bagedo General Hospital, Jeddah 22230, Saudi Arabia; alshanbariraghad.1996@gmail.com; 10 Department of Clinical Pharmacy, Northern Borders University, Rafha 91911, Saudi Arabia; malak.fahad.f@gmail.com; 11 Department of Pharmacy, Aldawaa Pharmacy, Arar 73551, Saudi Arabia; ranakallab@hotmail.com; 12 College of Medicine, Princess Noura University, Riyadh 12211, Saudi Arabia; dimaalassaff@gmail.com; 13 Department of Pharmacy, Altaawin Medical Clinics, Alkharj 16443, Saudi Arabia; ashwag.alsubaie0@gmail.com

**Keywords:** cephalexin, pharmacokinetics, systematic review, renal disease, clearance

## Abstract

Cephalexin is a first-generation β-lactam antibiotic used in adults and pediatrics to treat various streptococcal and staphylococcal infections. This review aims to summarize and evaluate all the pharmacokinetic (PK) data on cephalexin by screening out all pertinent studies in human beings following the per oral (PO) route. By employing different online search engines such as Google Scholar, PubMed, Cochrane Central, and Science Direct, 23 studies were retrieved, among which nine were in healthy subjects, five in diseased ones, and the remaining were drug–drug, drug–food, and bioequivalence-related. These studies were included only based on the presence of plasma concentration-time profiles or PK parameters, i.e., maximum plasma concentration (C_max_), half-life (t_1/2_) area under the curve from time 0-infinity (AUC_0–∞),_ and clearance (CL/F). A dose-proportional increase in AUC_0–∞_ and C_max_ can be portrayed in different studies conducted in the healthy population. In comparison to cefaclor, C_max_ was recorded to be 0.5 folds higher for cephalexin in the case of renal impairment. An increase in AUC_0–∞_ was seen in cephalexin on administration with probenecid, i.e., 117 µg.h/mL vs. 68.1 µg.h/mL. Moreover, drug–drug interactions with omeprazole, ranitidine, zinc sulfate, and drug–food interactions for cephalexin and other cephalosporins have also been depicted in different studies with significant changes in all PK parameters. This current review has reported all accessible studies containing PK variables in healthy and diseased populations (renal, dental, and osteoarticular infections, continuous ambulatory peritoneal dialysis) that may be favorable for health practitioners in optimizing doses among the latter.

## 1. Introduction

Cephalexin is a semi-synthetic broad-spectrum β-lactam antibiotic [1] that was developed in 1967, followed by its arrival on the market in 1969 and approval by the Food and Drug Administration (FDA) in 1971 [2]. Cephalexin is recommended to treat various ailments such as lower and upper respiratory tract infections, gonorrhea, urinary tract infections (acute and chronic), scarlet fever, streptococcal septicemia, and β-lactamase induced staphylococcal infections [3]. Moreover, it is also considered when treating skin infections [4], pre- and post-surgeries, to minimize the risk of infections at the surgery site [5]. It acts by hindering the final step of peptidoglycan synthesis, which is a main component of bacterial cell wall configuration, resulting in the death of cells [6]. It is available as a per oral (PO) formulation in different doses, such as 250 mg, 500 mg, and 1000 mg [7] and in an intravenous form [8].

Cephalexin is ranked as a class I drug in the Biopharmaceutics Classification System (BCS), having high intestinal permeability and solubility [9]. It depicts rapid absorption even in the fed state [10], with a high bioavailability of 90% [11]. The plasma protein binding of cephalexin with albumin is about 15% [12], with a volume distribution (V_d_) of 0.23 L/kg [13]. Cephalexin undergoes no detectable metabolism [14], with a half-life of 0.5–1.2 h [10], and is 90% eliminated unchanged from the kidney with a clearance of 4.3 mL/min/kg [11].

Cephalexin is an off-white translucent powder with a chemical formula of C_16_H_17_N_3_O_4_S [2]. It shows minute solubility in water, i.e., 10 mg/mL, but is insoluble in all organic solvents like alcohol, chloroform, and ether [12]. The lipophilicity (Log P) and acid dissociation constant (pKa) values of cephalexin are 0.65 and 3.2, respectively [2,15]. The human jejunal permeability (P_eff_) of cephalexin is 1.56 × 10^−4^ cm/s, depicting its utmost importance in drug absorption.

Cephalexin is classified in class B of the pregnancy category by the FDA and, thus, is not recommended unless needed. It produces a very small concentration in milk that is not likely to cause any damage to breastfeeding infants [16]. Its prototypical adverse drug reactions include abdominal pain, gastritis, diarrhea, vomiting, dyspepsia, vaginitis, thrombocytopenia, eosinophilia, candidiasis, neutrophilia, arthritis, arthralgia, etc. [1]. Cephalexin is contraindicated in those subjects who are allergic to penicillin or have hypersensitivity to any cephalosporin [17]. Moreover, repeated therapies may cause superinfections and different non-susceptible microorganisms [18]. The drug–drug interactions (DDI’s) of cephalexin with probenecid and metformin has been reported in the literature as causing changes in plasma concentration and renal clearance (CL_R_) of cephalexin [1] in some cases. The dose adjustment for cephalexin is required in the case of patients with renal failure where the chance of aggravation of adverse drug reactions and DDI’s are quite explicit.

The reasoning of the present study for executing this systematic review is to furnish a comprehensive picture of all the pharmacokinetic (PK) parameters of cephalexin in human subjects after the PO route of administration. The comprehension of the impact of changes in various PK variables and gaps in knowledge regarding preferred clinical responses may aid in pursuing personalized drug therapy for humans. Furthermore, this information may be further utilized in establishing PK models. In the published literature, only one review on cephalexin along with cefadroxil regarding the treatment of urinary tract infections has been reported previously [19]. As no systematic review has been conducted so far to present the PK of cephalexin concisely, the purpose of this study is to conduct a thorough literature review available until now on clinical PK, its parameters, drug–drug interactions, and drug–disease interactions that would be beneficial for health practitioners in the selection of doses of cephalexin in healthy and renally-impaired subjects.

## 2. Results

**Figure 1:**
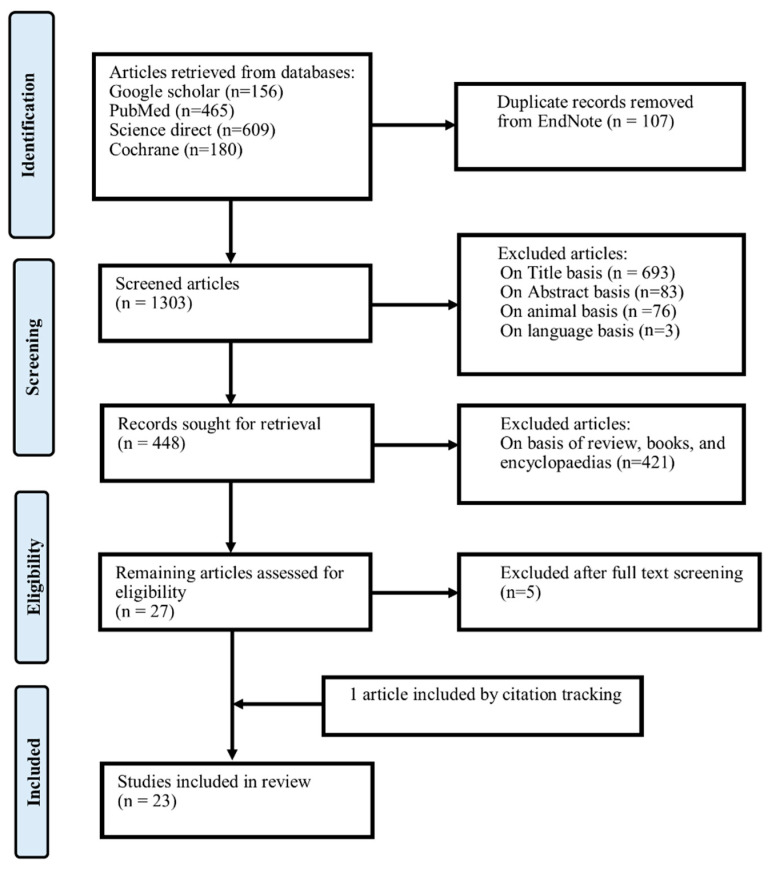
PRISMA flow chart for retrieval of relevant studies.

After a massive exploration of various databases, 1410 studies were obtained, among which 107 duplicate articles were found and eliminated. The 1303 left-over articles were then sorted according to the inclusion/exclusion basis. By clicking the option of “Relevant articles”, 01 article was further added, and ultimately, 23 articles are presented in the current study. The sorting process can be illustrated in Figure 1 as presented below. The particulars of all related studies containing the author’s name, population, number of participants, ethnicity, gender, age, dose, dosage form, frequency, list of different drugs being used, primary objective, and primary endpoints are represented in Table 1 and Table 2.

### 2.1. Results of the Evaluation of the Literature Quality

The quality of twenty-three included studies was evaluated utilizing the Jadad, CACPK, and CASP scoring tools. By Jadad’s scoring, 03 articles were moderate, and 20 were of low quality. The CACPK tool scoring showed that 19 studies were high, 01 was moderate, and 03 were of poor quality. Furthermore, by CASP scoring, 19 included studies were of high, and 04 were of moderate quality. The Cochrane Collaboration tool estimates the risk of bias in all studies, which indicated that 14 were at low risk and 09 were at high risk.

### 2.2. PO Studies of Cephalexin in Healthy Subjects

Among 23 studies, 09 studies were performed in healthy subjects following the PO route of administration for cephalexin. In a clinical study, CL/F was found to be greater in the case of the pretreatment of cephalexin with ammonium chloride as compared to that with sodium bicarbonate i.e., 245 ± 86 mL/min vs. 169 ± 45 mL/min [21]. Another study reported a comparison of cefroxadin and cephalexin in different doses (250 mg and 1000 mg) and formulations (solution, tablet, capsule) in which values of AUC_0-inf_ and t_1/2_ were significantly less than cephalexin (Table 3) [25]. One study reported a greater C_max_ of 32.7 ± 8.1 µg/mL for cephradine, followed by a decrease to 30.6 ± 3.6 µg/mL and 28.5 ± 14.5 µg/mL in the case of cephalexin capsules and tablets, respectively [30]. Another study reported a C_max_ of 21.29 µg/mL after administering a dose of 500 mg [26].

A comparison of cefaclor, cefadroxil, and CGP 9000 was undertaken with cephalexin in a study that showed greater AUC_0-inf_ of cefadroxil with 108.5 ± 18.4 µg.h/mL than with 93.0 ± 14.8 µg.h/mL for cephalexin [27]. Similarly, two other studies illustrated the same increase in AUC_0-inf_ of cefadroxil compared to cephalexin following the administration of 250 mg, 500 mg, and 1000 mg doses, and values can be seen in Table 3 [7,33]. Another study presented differences in PK parameters based on ethnicity and found greater CL/F in Chinese subjects than in Asian subjects, i.e., 258.89 ± 81.05 vs. 228.5 ± 38.29 [34].

### 2.3. Parenteral Study on Cephalexin in Healthy Subjects

In addition, CL/F was found to be slightly greater in the case of sodium cefazolin and significantly less in sodium cephalothin in comparison with parenteral sodium cephalexin at different doses of 0.5 g and 1 g (Table 3) [8]. The details of additional PK parameters are given in Table 3.

### 2.4. PO Studies on Cephalexin in Diseased Subjects

In patients with renal failure, the C_max_ has been reported to be greater for cephalexin in contrast to cefaclor, i.e., 27.6 ± 6.4 µg/mL vs. 23.1 ± 7.7 µg/mL, after taking a dose of 500 mg [11]. A study conducted in pediatrics and adults with cystic fibrosis and their comparison with healthy adults depicted an 11% and 44% decrease in AUC_0-inf_ among the former in comparison to the latter, respectively [36]. Following the administration of a PO dose of 500 mg, patients with dental infections reported a C_max_ of 10.58 ± 1.63 µg/mL in a clinical study [37]. In the case of continuous ambulatory peritoneal dialysis patients (CAPD), the AUC_0-inf_ of PO cephalexin showed a 2-fold decrease compared to intravenous/intraperitoneal cefazolin [38]. Another study presented a C_max_ of 50 (20–126) µg/mL upon administering a 40 mg/kg/dose in pediatrics suffering from osteoarticular (OA) infections [39]. Other PK parameters are presented in Table 4.

### 2.5. Drug–Drug Interaction Studies on Cephalexin

The AUC_0-inf_ of PO cephalexin was reported to be 1.8 folds increased in one study [28], whereas a decrease of 2 fold in CL/F for parenteral sodium cephalexin in another study [8] on the co-administration of probenecid in comparison to being taken alone. In addition, upon the administration of ranitidine and omeprazole in different doses (20 mg and 40 mg) with cephalexin, a slight decrease in C_max_ and an increase in CL/F of the latter has been depicted in a clinical study (Table 5) [29]. Another study presented a slight reduction in C_max_ of cephalexin when given along with zinc sulfate, i.e., 12.46 ± 2.73 µg/mL vs. 18.07 ± 4.27 µg/mL [31].

### 2.6. Drug–Food Interaction Studies on Cephalexin

One study narrated a 1.9-fold decrease in C_max_ of cephalexin and cefroxadin in the fed state as compared to the fasting state after the administration of a similar dose of 250 mg [25]. In addition, AUC_0-inf_ was found to be slightly decreased in the postprandial state in two clinical studies, one of which was conducted in adults on cephalexin [24] and the other in infants and pediatrics comparing the PK of cephalexin, cephradine, cefaclor, and cefadroxil, whose values can be discerned in Table 5 [35].

### 2.7. Bioequivalence/Bioavailability Studies on Cephalexin

A study narrated the bioequivalence between the reference (Ospexin^®^ Cephalexin 500 mg, lot no: AG5886, expiry date: April 2013, manufactured by Sandoz, Australia) and test 1 (MPI cephalexin tablet) and test 2 (MPI cephalexin capsule), among which values of all PK parameters were found to be almost the same [20]. Similarly, a comparison of bioavailability was reported between the standard (Keflex^®^ manufactured by Lilly pharmaceutical company, England) and the other four brands (J.I, J.II, L.I, L.II) in which the L.I brand showed greater C_max_ in comparison to the standard brand, whereas others depicted a slight decrease, respectively [22]. One study presented the bioequivalence of two of the same brands, one of which showed the AUC_0-inf_ was significantly less in cephalexin B (distributed to the government health sector) as compared to cephalexin A (distributed to the private health sector), i.e., 17.2 ug. h/mL vs. 11.93 ug. h/mL [23]. Another study compared the bioequivalence of reference formulation A (Keflex^®^ capsules product of Eli Lilly Italia S.P.A. Sesto Fiorentino-Florence, Italy, Lot No. S220319) with test formulation B (Amilexin^®^ capsules product of Amipharma Laboratories Ltd., Sudan, Batch No. CPF0) and test formulation C (Sigmacef^®^ capsules Sigma Tau SUDAN Ltd Drug Store, Sudan, Batch No. Ts 029-00). It depicted a decrease in CL/F in B, whereas an increase in C compared to the reference formulation, as can be seen in Table 6 [32].

## 3. Discussion

This systematic review focused on collating and analyzing all the PK variables of cephalexin from the published research articles that involved human subjects. Out of twenty-three articles, nine were reported in healthy subjects, five were in diseased individuals (renal disease, CAPD, cystic fibrosis, dental infections, OA infections), four were related to DDI’s, three were drug–food interactions and four were concerned with bioequivalence.

A linear dose–concentration relationship was found in cephalexin at various doses that was perceived from values of C_max_ and AUC_0-inf_. Moreover, cephalexin showed a slightly higher AUC_0-inf_ than cefroxadin, which depicted a greater residence of the former in circulation which may require monitoring [25]. In a comparison between the two cephalosporins, the t_1/2_ of cefadroxil was found to be longer, and its plasma levels were higher than the published minimum inhibitory concentration (MIC) values for sensitive organisms for lengthy durations than cephalexin, which suggests using small, repeated dosing of the former drug [7,33]. Due to its shorter half-life of 1.5–2 h, cephalexin is liable to exhibit the flip–flop pharmacokinetics, as mentioned in a previously published study [40] and, thus, has therapeutic implications in predicting drug–drug interactions and toxicity with respect to the outcomes. Similarly, another clinical study compared the PK of four different drugs (cefaclor, cefadroxil CGP 9000, and cephalexin), among which cefadroxil showed a higher AUC_0-inf_, suggesting that it is the most favorable for antibiotic therapy [27].

The renal clearance (CL_R_) of cephalexin was greater in the case of pretreatment with ammonium chloride in comparison to sodium bicarbonate, which may be due to functional changes in the H^+^/peptide co-transporter 2 (PEPT2) and, in turn, the pH of urine [21]. Moreover, no significant variations in PK parameters were observed between cephradine and two formulations of cephalexin (tablets and capsules). The study proposed the same bioavailability in all. The clearance values of both drugs have been reported as greater than 125 mL/min, suggesting their clearance through two processes, i.e., tubular secretion and glomerular filtration [30]. Inter-ethnic and PEPT2 genotype differences were ruled out for cephalexin, in which its C_max_ among the Chinese population was markedly less than the Asian population, which may be due to the greater mean body weight in the former [34].

Cephalexin steadily achieves a plasma therapeutic concentration better than cefaclor, resulting in more accumulation among renally-compromised patients, suggesting dose adjustments in the former [11]. In cystic fibrosis, the CL_R_ of cephalexin was found to be higher in pediatric patients as compared to adults, which may suggest that a high dose is required for pediatric patients [36]. The peritoneal CL/F of cephalexin and cefazolin was not reported to be high in a clinical study, meaning no changes are required in CAPD patients in contrast to those having renal impairment [38]. In the case of OA infections, the AUC_0-tau_ was found to be significantly greater for children aged between 1 and 6 y than for ages 7–16 y, resulting in the requirement for close monitoring of younger children [39]. The differences in PK parameter values have been seen with respect to the population’s age (adults and pediatrics), ethnicity (Chinese and Asian), and disease state (renal failure, CAPD, and cystic fibrosis), as mentioned individually above. The analysis of patients with different diseases according to their PK parameters may provide individualized dosing regimens as significant changes can be seen with respect to the comparison of cephalexin with any other cephalosporin drug.

DDI’s are very important to mention as they widely impact the PK and pharmacodynamics (PD) of the drug under consideration. Mainly, DDI’s produce negative effects, but some can result in beneficial outcomes. The AUC_0-inf_ of cephalexin increases with the co-administration of probenecid, resulting in a greater likelihood of achieving PK/PD goals. This depicts that probenecid–cephalexin-boosted regimens may be used as an alternative to intravenous cefazolin–probenecid in treating Gram-positive cocci infections [28]. Moreover, in another study, probenecid increased the serum t_1/2_ and decreased the CL_R_ of cephalexin, which may be due to the impairment of its tubular secretion [8]. One clinical study narrated the interactions between ranitidine and omeprazole. The study showed no notable changes in any PK parameter, except for T_max_, which was delayed, and, in turn, the amount of time in which serum concentrations persisted above the MIC_90_ (T > MIC_90_) of susceptible organisms (Staphylococcus aureus and Streptococcus pyogenes) in a course with dose interval decreases, thus encouraging maximum dosing of cephalexin [29]. Similarly, the intake of zinc sulfate with cephalexin decreases T > MIC_90_ microorganisms, i.e., Staphylococcus aureus, and values of all PK parameters, such as C_max_, T_max_, and AUC_0-inf_, that may affect the outcomes of the standard therapy of cephalexin, resulting in a decrease in the dose [31].

Both cephalosporins (cephalexin and cefroxadin) showed a significant decrease in C_max_ in the fed state, depicting delayed absorption in the gastrointestinal tract [25]. One study reported a greater C_max_ of cephalexin at the 20th hour than at the 8th hour after the intake of food, which may show the effect of time in the postprandial state as compared to the fasting condition [24]. In another study, the effect of food on cephalexin and cephradine was promptly indicated by a decrease in C_max_ and AUC_0-inf_ in comparison to cefadroxil and cefaclor, which may be due to changes in PK features of the latter that were considered to be the predecessors of the former [35]. The bioequivalence of cephalexin was investigated in several clinical studies, among which one was between different formulations [20], the other was between different brands [22], and the third was among formulations from different countries [32]. All depicted similar PK parameters and were thus found to be bioequivalent and require no dosage adjustments in patients when using any brand or formulation.

Overall, the studies had variations with respect to study design and methods to measure the plasma concentration, but most studies followed the randomized crossover protocol and the analysis method of high-pressure liquid chromatography (HPLC), which depicted less effect on the PK parameter values among all.

The firmness of this current study is that it encompassed all studies on cephalexin concerning the PK from its inception until 20 June 2023. Four databases were thoroughly screened to maximize the validity and reliability of the findings. Moreover, the differences in PK owing to parenteral formulation, ethnicity, and special population (pediatric) are explicitly explained, due to which chances of bias were reduced to a great extent. However, scarce data are available on diseased populations. Therefore, more studies are required in the future to further elaborate different perspectives.

## 4. Materials and Methods

### 4.1. Study Design and Search Strategy

**Figure 2:**
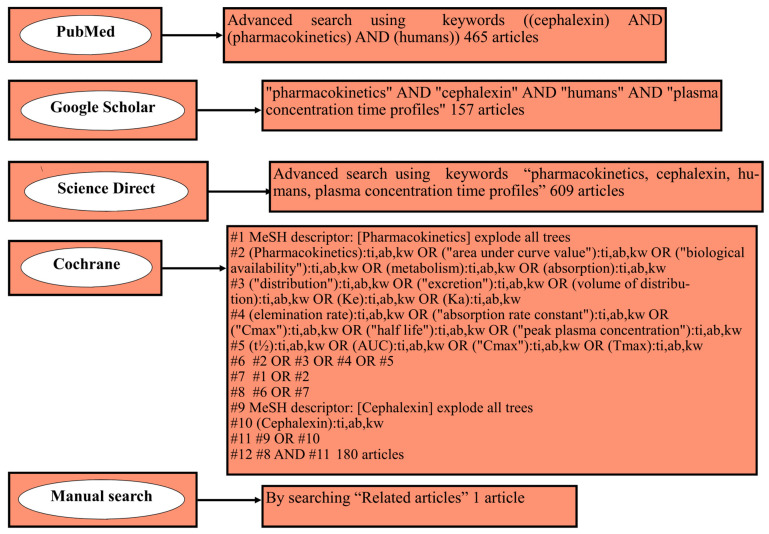
Flow chart of the literature search strategy.

This methodical search was performed following the standard guidelines of Preferred Reporting Items for Systematic Reviews and Meta-Analysis (PRISMA) [41] and the Cochrane updated Handbook guidelines [42]. This systematic review has been registered in the International Prospective Register of Systematic Reviews (PROSPERO) with registration number of CRD42023456056. Different online databases, such as PubMed, Google Scholar, Science Direct, and Cochrane, were utilized for performing an exhaustive search to retrieve all the pertinent articles concerning the PK of cephalexin from the inception on 20 March 2023 to 20 June 2023. The development of the research question was carried out by utilizing the Sample, Phenomenon of Interest, Design, Evaluation, and Research (SPIDER) tool. The keywords used to carry out the systematic search were pharmacokinetics, cephalexin, humans, plasma concentration-time profiles, the area under the curve (AUC), biological availability, metabolism, absorption, distribution, excretion, V_d_, elimination rate, absorption rate constant, peak plasma concentration (C_max_), half-life (t_1/2_), and time to reach maximum plasma concentration (T_max_). Furthermore, citation tracking was conducted by thoroughly searching “Related articles”. The details of the literature search can be seen in Figure 2.

### 4.2. Eligibility Criteria

Relevant clinical research studies that met the standards of the inclusion criteria, i.e., studies conducted in humans (healthy and diseased) after PO administration of cephalexin, narrating at a minimum of one PK parameter, such as C_max_, the area under the concentration-time curve from 0–∞ (AUC_0–∞_), oral clearance (CL/F), t_1/2_, or presence of time vs. plasma concentration graphs, were included. Moreover, only articles published in the English language were added to the review.

### 4.3. Selection of Clinical Studies

The retrieved studies from all online search databases were then exported to EndNote version X9 for the detection and removal of duplicates. The articles were then sorted for exclusion based on title, abstract, language, and animal studies, whose details are explicitly presented in Supplementary Table S1. Books, reviews, encyclopedias, letters to editors, conference abstracts, and short commentaries were also precluded. The articles that complied with the eligibility assessment were then further screened by reading wholly, and then, purely suitable articles were embodied in the review. The flow chart for the process of study retrieval can be seen in Figure 2.

### 4.4. Extraction of Data from Relevant Studies

The extraction of data was conducted for each study. The data comprised the name of the author, gender, population, ethnicity, age, study size, different medications (either for comparison or DDI), administration dose, frequency, dosage form, primary objective, and primary endpoint. Two independent reviewers (M.K. and S.A.) executed the whole process of data retrieval and assessment of eligibility. A third reviewer (A.F.) further evaluated the extraction of data to enhance the reliability of results. To conduct a facile evaluation of the outcomes and sustain uniformity, the units of all clinical PK parameters (C_max_, AUC_0–∞_, T_max_, CL/F, and t_1/2_) were assimilated into similar units. Furthermore, non-compartmental analysis (NCA) was undertaken to extract the values of C_max_, AUC_0–∞_, T_max_, CL/F, and t_1/2_ from the research articles where time and concentration values were given at different points.

### 4.5. Assessment of Literature Quality

The literature quality of each of the selected articles was appraised by employing Jadad scoring [43], critical appraisal for clinical pharmacokinetic studies (CACPK) [44], and the Critical Appraisal Skill Programme (CASP) scoring [45] tools, whose particulars are cited in Supplementary Tables S2–S4. Furthermore, Supplementary Table S5 explicitly chronicles the risk of bias in pertinent studies by utilizing the Cochrane collaboration tool [46]. All these standardized tools pave the way to clearly assess the quality of the articles being added to the study by employing point-to-point scoring for each question, evaluating the whole paper thoroughly, and, in turn, increasing the reliability. In Jadad scoring (5-item questionnaire), a score “0” or “1” was given to all studies based on 3 main particulars, i.e., randomization (0–2 marks), blinding (0–2 marks), and withdrawal (0–1 marks). Articles that scored > 4, 3–4, and <3 were of high, moderate, and low quality, respectively. The CACPK tool is a 21-item checklist that is ranked for every question as yes, no, and I don’t know. A score of >13, 12–13, and <12, was considered to be high, fair to moderate, and poor quality, respectively. CASP scoring evaluates the validity and transparency of relevant articles by utilizing the options of “Yes”, “No”, and “Can’t tell”, in which points of <4 present low quality, 4–6 show moderate quality, and >6 show high quality. The Cochrane Collaboration tool evaluates the risk of bias by responding to 6 questions and categorizing them into low, moderate, and unclear risk.

## 5. Conclusions

This systematic review epitomized all previously reported research studies on cephalexin to pool data on its clinical PK, DDI’s, drug–food interactions, and bioequivalence. The AUC_0–∞_ and C_max_ increased with dose in linear proportionality among healthy subjects. The T_max_ and AUC_0–∞_ significantly increased in patients with renal impairment. DDI with probenecid increased the clinical efficacy of cephalexin, which is important in further PD studies. The displayed results provide researchers with an opportunity to develop and evaluate the PK models and adverse drug reactions occurring from DDI’s so they can be avoided. Furthermore, this may assist clinicians in decision-making regarding the adjustment of doses in diseased subjects, thus furnishing the perception of personalized drug therapy.

## Figures and Tables

**Table 1:** Characteristics of studies in a healthy population.

Sr. No.	Author	Population (Ethnicity)	Study Size (N)	Age (Years)	Gender	Medication Used	Dose (mg)	Dosage Form	Frequency	Primary Objective	Primary Endpoint
1	Liew et al. [20]	Healthy (Malay)	24	20–41	M	Reference cephalexin	500	Tab	OD	PK, BE	PK parameters
						Test cephalexin 1		Tab			
						Test cephalexin 2		Cap			
2	Liu R et al. [21]	Healthy (Asian)	16	22.8–36.8	M	Cephalexin	1000	Syrup ^a^	OD	PK	PK parameters
3	Hassanzadeh et al. [22]	Healthy (N/R)	8	22–28	M	Standard cephalexin	500	Cap	N/R	PK, BA	PK parameters
						4 different brands ^b^					
4	Bataineh et al. [23]	Healthy (Jordian)	18	20–25	N/R	Cephalexin A	500	Cap	OD	PK, BE	PK parameters
						Cephalexin B					
5	Ding et al. [24]	Healthy (Chinese)	20	30–37	M	Cephalexin	500	Cap	OD	PK	PK parameters
6	Lecailllom et al. [25]	Healthy (N/R)	21	22–50	N/R	Cephalexin	250, 1000,	Solution, Cap	N/R	PK	PK parameters
						Cefroxadin	250, 1000	Solution, Tab			
7	Nakagawa et al. [26]	Healthy	3	28–37	M	Cephalexin	500	Cap	OD	PK	PK parameters
8	Lode et al. [27]	Healthy (N/R)	12	20–41	6 M, 6 F	Cephalexin	1000	Cap	OD	PK	PK parameters
						Cefaclor					
						Cefadroxil					
						CGP 9000					
9	Evert et al. [28]	Healthy (N/R)	11	18–29	4 M, 7 F	Cephalexin	1000	Cap	OD	PK	PK parameters
						Probenecid	500				
10	Kelly et al. [29]	Healthy (N/R)	21	27–31	16 M, 5 F	Cephalexin	500	Cap	Single/Multiple	PK	PK parameters
						Ranitidine	150				
						Omeprazole	20, 40				
11	Barbhayia et al. [7]	Healthy (N/R)	36	21.3–3.1	M	Cephalexin	250, 500, 1000,	Solution	OD	PK	PK parameters
						Cefadroxil	250, 500, 1000,				
12	Finkelstein et al. [30]	Healthy (N/R)	9	23–38	M	Cephradine	1000	Cap	Q.I.D for 5 doses	PK	PK parameters
						Cephalexin		Cap			
						Cephalexin		Tab			
13	Ding Y et al. [31]	Healthy (N/R)	12	30–40	M	Cephalexin	500	Cap	N/R	PK	PK parameters
						Zinc sulfate	250	Tab			
14	Mohamed S et al. [32]	Healthy (Sudanese)	24	20–38	M	Reference formulation A	500	Cap	OD	PK, BE	PK parameters
						Test formulation B					
						Test formulation C					
15	Welling et al. [33]	Healthy (N/R)	12	21–29	M	Cephalexin	500	Cap	OD	PK	PK parameters
						Cefadroxil					
16	Liu R et al. [34]	Healthy (Asian)	288			Cephalexin	1000	Syrup ^a^	OD	PK	PK parameters
		Chinese	15	20–29.8	M						
		Asian Indians	15	22.5–30.7	M						
17	Regamey et al. [8]	Healthy (N/R)	4	28–32	M	Sodium cephalexin	0.5 ^c^	IV infusion/IM	OD	PK	PK parameters
						Sodium cephalexin + Probenecid	0.5 ^c^ + 1 ^c^	IV infusion/PO			
						Sodium cefazolin	0.5 ^c^	IV infusion			
						Sodium cephalothin	1 ^c^	IV infusion			

M: Male, F: Female, Tab: Tablet, Cap: Capsule, PK: Pharmacokinetics, BE: Bioequivalence, BA: Bioavailability, OD: Once a day, T.I.D: Thrice a day, Q.I.D: Four times a day, IV: Intravenous, IP: Intra-peritoneal, IM: Intramuscular, PO: Per oral, N/R: Not reported ^a^ 40 mL cephalexin syrup 125 mg/5 mL Keflex, Advancis USA, ^b^ 4 different brands are J.I, J.II, L.I, L.II, ^c^ dose in gram.

**Table 2:** Characteristics of studies in diseased population.

Sr. No.	Author	Population (Ethnicity)	Study Size (N)	Age (Years)	Gender	Medication Used	Dose (mg)	Dosage Form	Frequency	Primary Objective	Primary Endpoint
1	Ginsburg et al. [35]	Diseased (UTI) infants and children	17	4.9 ^a^	N/R	Cefadroxil	15 ^c^	Susp.	N/R	PK	PK parameters
			28 (15) (13)	7.5 ^a^			14.1 ^c^ 15 ^c^	Cap Susp.			
			14	18.6 ^b^		Cefaclor	15 ^c^	Susp.			
			20	21 ^b^		Cephalexin	15 ^c^	Susp.			
			16	3.5 ^a^		Cephradine	15 ^c^	Susp.			
2	Spyker et al. [11]	Diseased (renal)	24	22–76	M	Cefaclor	500	N/R	OD	PK	PK parameters
						Cephalexin					
3	Nahata et al. [36]	Diseased (cystic fibrosis)									
		Pediatric	7	6.7–12.3	2 M, 5 F	Cephalexin	250, 500	Susp.	OD	PK	PK parameters
		Adult	4	21–34.2	3 M, 1 F		500				
		Healthy adult	4	23.6–35.2	2 M, 2 F		500				
4	Akimoto et al. [37]	Diseased (Dental infection)	57	18–54	20 M, 37 F	Cephalexin	500	Cap	OD	PK	PK parameters
5	Bunke et al. [38]	Diseased (CAPD)	N/R	36.4–70.4	N/R	Cefazolin	10 ^c^	IV/IP	OD	PK	PK parameters
						Cephalexin	500	N/R			
6	Autmizguine et al. [39]	Diseased (OA infection) (Paediatric)	12	1–16	7 M, 5 F	Cephalexin	40 ^d^	Cap/Susp.	T.I.D	PK	PK parameters

M: Male, F: Female, Tab: Tablet, Cap: Capsule, Susp.: Suspension, PK: Pharmacokinetics, BE: Bioequivalence, BA: Bioavailability, OD: Once a day, T.I.D: Thrice a day, Q.I.D: Four times a day, PO: Per oral, UTI: Urinary tract infections, CAPD: Continuous ambulatory peritoneal dialysis, OA: Osteoarticular, N/R: Not reported, ^a^ age in mean, ^b^ age in months, ^c^ dose is in mg/kg, ^d^ mg/kg/dose.

**Table 3:** PK parameters of cephalexin in healthy subjects.

					Plasma Pharmacokinetic Parameters				
Sr. No.	Author			Dose (mg)	C_max_ (µg/mL)	AUC_0-inf_ (µg.h/mL)	T_max_ (h)	CL/F (mL/min)	t_1/2_ (h)
**Oral studies**									
1	Liu R et al. [21]	Cephalexin (Pooled)	Ammonium chloride	1000	32.8 ± 8.2	74.6 ± 11.2 ^a^	1	245 ± 86	1.11 ± 0.13
			Sodium bicarbonate		32.6 ± 7.4	73.0 ± 11.0	1	169 ± 45	1.11 ± 0.18
2	Lecailllom et al. [25]	Cephalexin		250 (solution)	9.9 ± 0.4	14.6 ± 1.3	0.58	N/R	0.74 ± 0.06
		Cefroxadin		250 (solution)	10.1 ± 1.1	12.8 ± 1.1	0.58		0.61 ± 0.02
		Cephalexin		1000 (solution)	26.3 ± 3.4	56.6 ± 5.9	0.75		0.98 ± 0.17
		Cefroxadin		1000 (solution)	26.5 ± 2.7	50.3 ± 4.9	0.75		0.93 + 0.16
		Cephalexin		1000 (film-coated tablets)	30.1 ± 7.3	60.2 ± 7.2	0.95		0.91 ± 0.11
		Cefroxadin		1000 (capsules)	25.0 ± 4.6	51.2 ± 6.4	1.1		0.84 ± 0.10
3	Nakagawa et al. [26]	Cephalexin		500	21.29	30.14	1	276.22	1.3
4	Lode et al. [27]	Cephalexin		1000		93.0 ± 14.8			
		Cefaclor				74.5 ± 9.9			
		Cefadroxil				108.5 ± 18.4			
		CGP 9000				70.1 ± 9.0			
5	Barbhayia et al. [7]	Cephalexin		250	12.0 ± 4.2	14.1 ± 1.7	0.6 ± 0.1	240 ± 35	1.1 ± 0.2
				500	20.7 ± 3.2	29.0 ± 4.0	0.6 ± 0.1	250 ± 54	1.0 ± 0.2
				1000	42.0 ± 5.3	59.9 ± 6.7	0.6 ± 0.1	208 ± 30	1.1 ± 0.1
		Cefadroxil		250	8.7 ± 0.9	22.6 ± 2.3	0.8 ± 0.1	146 ± 17	1.9 ± 0.3
				500	15.1 ± 2.8	44.8 ± 6.9	1.3 ± 0.4	170 ± 36	2.1 ± 0.6
				1000	30.8 ± 3.2	93.1 ± 12.8	0.9 ± 0.1	140 ± 37	2.1 ± 0.3
6	Finkelstein et al. [30]	Cephradine		1000	32.7 ± 8.1	61.86 ± 11.19	1.15 ± 0.36	239.3 ^b^	N/R
								277.7 ^c^	
		Cephalexin (capsule)			30.6 ± 3.6	52.76 ± 9.35	1.0 ± 0.19	308.5 ^b^	
								325.7 ^c^	
		Cephalexin (tablet)			28.5 ± 14.5	51.34 ± 6.96	1.10 ± 0.24	321.3 ^b^	
								329.7 ^c^	
7	Welling et al. [33]	Cephalexin		500	17.5 ± 4.3	24.9 ± 5.1	1.02 ± 0.38	N/R	N/R
		Cefadroxil			16.0 ± 3.1	40.8 ± 9.7	1.8 ± 0.5		
8	Liu R et al. [34]	Cephalexin (Chinese)		1000	29.80 ± 4.09	63.20 ± 13.75	0.5–1.5	258.89 ± 81.05	1.04 ± 0.20
		Cephalexin (Asian Indian)			33.29 ± 4.97	68.61 ± 12.31	1.0–1.0	228.5 ± 38.29	0.99 ± 0.16
**Parenteral study**									
9	Regamey et al. [8]	Sodium cephalexin		0.5 g	N/R	N/R	N/R	252.2 ± 4.7 ^d^	0.92 ± 0.02
		Sodium cephalexin + Probenecid		0.5 g + 1 g				123.9 ± 8.8 ^d^	1.71 ± 0.08
		Sodium cefazolin		0.5 g				62.3 ± 1.8 ^d^	1.80 ± 0.16
		Sodium cephalothin		1 g				267.0 ± 69.7 ^d^	0.47 ± 0.03

C_max_: Maximum plasma concentration, AUC_0-inf_: Area under the curve from time 0 to infinity, T_max_: Time to reach maximum plasma concentration, CL/F: Oral clearance, t_1/2_: Half-life, N/R: Not reported, ^a^ AUC is reported as AUC_0–8_, ^b^ CL is renal, ^c^ CL is total body clearance (TBC), ^d^ CL is in mL/min/1.73 m^2^.

**Table 4:** PK parameters of cephalexin in diseased subjects.

				Plasma Pharmacokinetic Parameters				
Sr. No.	Author		Dose (mg)	C_max_ (µg/mL)	AUC_0-inf_ (µg.h/mL)	T_max_ (h)	CL/F (mL/min)	t_1/2_ (h)
1	Spyker et al. [11]	Cefaclor	500	23.1 ± 7.7 ^a^	N/R	0.88 ± 0.33	N/R	N/R
		Cephalexin		27.6 ± 6.4		1.35 ± 0.83		
2	Nahata et al. [36]	Cephalexin		N/R		N/R		N/R
		Pediatric	250, 500		0.185 ^b^		5.85 ^c^	
		Adult	500		0.242 ^b^		4.61 ^c^	
		Healthy adult	500		0.272 ^b^		N/R	
3	Akimoto et al. [37]	Cephalexin	500	10.58 ± 1.63	N/R	1.49 ± 0.02	N/R	N/R
4	Bunke et al. [38]	Cefazolin IV	10 ^d^	N/R	2016	N/R	5.7	N/R
		Cefazolin IP	10 ^d^		1580		5.85	
		Cephalexin	500		489		18.47	
5	Autmizguine et al. [39]	Cephalexin				N/R		
		(Pediatric)	40 ^e^	50 (20–126)	138 (75–245) ^f^		4.83 (2.83–8.99)	1.1 (0.7–4.0)

C_max_: Maximum plasma concentration, AUC_0-inf_: Area under the curve from time 0 to infinity, T_max_: Time to reach maximum plasma concentration, CL/F: Oral clearance, t_1/2_: Half-life, N/R: Not reported, ^a^ C_max_ is in µg/mL/70 kg, ^b^ AUC is in AUC/dose/kg (mL/min/kg), ^c^ CL is in mL/min/kg, ^d^ Dose is in mg/kg, ^e^ Dose is in mg/kg/dose ^f^ AUC is presented as AUC_0-tau_.

**Table 5:** Drug–drug and drug–food interaction studies on cephalexin in healthy and diseased subjects.

					Plasma Pharmacokinetic Parameters				
Sr. No.	Author			Dose (mg)	C_max_ (µg/mL)	AUC_0-inf_ (µg.h/mL)	T_max_ (h)	CL/F (mL/min)	t_1/2_ (h)
**Drug–drug interaction**									
1	Evert et al. [28]	Cephalexin		1000	26.9	68.1	1.9	244.9	1.14
		Cephalexin + Probenecid		500	35.8	117	2.7	141.61	1.47
2	Kelly et al. [29]	Cephalexin		500	16	32.7	1.19	274.89	0.6
		Cephalexin + Ranitidine		150	15.2	34.2	1.48	288.21	0.69
		Cephalexin		500	17.7	33.8	0.76	271.55	0.51
		Cephalexin + Omeprazole		20	16.3	35.6	1.38	291.55	0.51
		Cephalexin		500	17.6	36	0.94	293.21	0.5
		Cephalexin + Omeprazole		40	15.5	33.4	1.09	293.21	0.45
3	Regamey et al. [8]	Sodium cephalexin		0.5 g	N/R	N/R	N/R	252.2 ± 4.7 ^a^	0.92 ± 0.02
		Sodium cephalexin + Probenecid		0.5 g + 1 g				123.9 ± 8.8	1.71 ± 0.08
4	Ding Y et al. [31]	Cephalexin		500	18.07 ± 4.27	41.97 ± 6.04	1	N/R	1.50 ± 0.58
		Cephalexin + Zinc sulfate		250	12.46 ± 2.73	30.47 ± 3.52	1		1.13 ± 0.27
		Zinc sulfate 3 h before cephalexin		250	16.00 ± 4.06	34.37 ± 1.58	1		1.65 ± 0.79
		Cephalexin 3 h before zinc sulfate		250	17.35 ± 3.67	41.13 ± 6.62	1		1.28 ± 0.26
**Drug–food interaction**									
1	Lecailllom et al. [25]	(Fasting)	Cephalexin	250	10.1 ± 2.3	15.1 ± 2.2	0.67	N/R	0.67 ± 0.06
			Cefroxadin	250	10.4 ± 2.5	13.1 + 1.8	0.55		0.62 ± 0.07
		(Fed)	Cephalexin	250	5.9 ± 2.0	13.6 ± 1.5	1.13		1.00 ± 0.20
			Cefroxadin	250	6.3 ± 2.5	12.4 ± 1.2	0.96		0.97 ± 0.20
2	Ding et al. [24]	Cephalexin		500					
		Postprandial (*n* = 10)							
		8:00 h			18.98 ± 3.8	38.25 ± 7.8	1(0.75, 1.5)		1.17 ± 0.3
		20:00 h			15.29 ± 3.0	36.65 ± 3.7	0.88(0.5, 1.5)		1.64 ± 0.6
		Fasting (*n* = 10)							
		8:00 h			18.0 ± 3.7	38.59 + 5.2	1.25(0.75, 1.5)		1.28 ± 0.4
		20:00 h			17.0 ± 3.2	35.33 ± 4.5	1(0.75, 1.5)		1.17 ± 0.2
3	Ginsburg et al. (UTI patients) ^c^ [35]	Cephalexin (Fasting)		15 ^b^	N/R	40	N/R	N/R	0.96
		Fed				23			0.98
		Cephradine (Fasting)		15 ^b^		29			0.8
		Fed				23			1
		Cefaclor (Fasting)		15 ^b^		20			0.6
		Fed				18			0.76
		Cefadroxil							
		Suspension (Fasting)		15 ^b^		41			1.33
		Fed				39			1.5
		Capsule (Fasting)		12–18 ^b^		44			1.4
		Fed				43			2

C_max_: Maximum plasma concentration, AUC_0-inf_: Area under the curve from time 0 to infinity, T_max_: Time to reach maximum plasma concentration, CL/F: Oral clearance, t_1/2_: Half-life, N/R: Not reported, ^a^ CL is in mL/min/1.73 m^2^, ^b^ Dose is in mg/kg, ^c^ infants and pediatric patients.

**Table 6:** Bioequivalence studies on cephalexin.

				Plasma Pharmacokinetic Parameters				
Sr. No.	Author		Dose (mg)	C_max_ (µg/mL)	AUC_0-inf_ (µg.h/mL)	T_max_ (h)	CL/F (mL/min)	t_1/2_ (h)
1	Liew et al. [20]	Reference ^a^	500	17.39 (4.15)	30.07 (5.94)	0.85 (0.26)	13.75 (2.39)	1.08 (0.21)
		Test 1 ^b^		18.29 (3.01)	31.33 (5.18)	0.69 (0.23)	13.15 (2.41)	1.10 (0.30)
		Test 2 ^c^		18.25 (3.92)	31.22 (5.29)	0.77 (0.18)	13.16 (2.19)	1.12 (0.26)
3	Hassanzadeh et al. [22]	Keflex	500	16.2	28.3	1.1	304.87	1.1
		J.I		14.3	24.4	1.1	356.52	1.1
		J.II		15	28.6	1.1	306.54	1.1
		L.I		18.1	32	1.1	269.89	1.2
		L.11		13.3	28.5	1.3	304.87	1
		Mean ± SD		15.4 ± 1.8	28.4 ± 2.7	1.1 ± 0.1	308.21 ± 29.98	1.1 ± 0.1
4	Bataineh et al. [23]	Cephalexin A ^d^	500	10.782	17.28	0.61	N/R	N/R
		Cephalexin B ^e^		6.839	11.93	0.59	N/R	N/R
11	Mohamed S et al. [32]	Reference formulation A ^f^	500	19.05 ± 4.95	33.35 ± 4.97	1.05 ± 0.26	255.56 ± 43.64	0.98 ± 0.13
		Test formulation B ^g^		17.36 ± 4.07	35.07 ± 7.35	1.10 ± 0.29	246.73 ± 49.64	0.99 ± 0.21
		Test formulation C ^h^		17.31 ± 5.28	32.46 ± 13.34	1.10 ± 0.29	296.71 ± 116.28	0.98 ± 0.16

C_max_: Maximum plasma concentration, AUC_0-inf_: Area under the curve from time 0 to infinity, T_max_: Time to reach maximum plasma concentration, CL/F: Oral clearance, t_1/2_: Half-life, N/R: Not reported, ^a^ Reference: Ospexin^®^, ^b^ Test 1: MPI cephalexin tablet, ^c^ Test 2: MPI cephalexin capsule, ^d^ Cephalexin A: distributed to the private health sector, ^e^ Cephalexin B: distributed to the government health sector, ^f^ Reference formulation A: Keflex^®^ capsules, ^g^ Test formulation B: Amilexin^®^ capsules, ^h^ Test formulation C: Sigmacef^®^ capsules.

## Data Availability

All the data used for this publication are either presented in the main article or are available as Supplementary Material.

## Supplementary Materials

The following supporting information can be downloaded at https://www.mdpi.com/article/10.3390/antibiotics12091402/s1, Table S1: Screening of articles based on title, abstract, presence of animals and language, Table S2: Quality assessment of included articles by Jadad scoring, Table S3: Quality assessment of included articles by CACPK scoring, Table S4: Quality assessment of included articles by CASP scoring, Table S5: Risk of bias assessment by the Cochrane Collaboration tool.

## References

[1] Herman T.F.H.M. (2023). Cephalexin. [Updated 2022 Aug 18]. StatPearls [Internet].

[2] PubChem Compound Summary for CID 27447, Cephalexin. https://pubchem.ncbi.nlm.nih.gov/compound/Cephalexin.

[3] Bailey A., Walker A., Hadley A., James D.G. (1970). Cephalexin—A new oral antibiotic. Postgrad. Med. J..

[4] Derrick C.W., Reilly K. (1983). The role of cephalexin in the treatment of skin and soft-tissue infections. Postgrad. Med. J..

[5] Valent A.M., DeArmond C., Houston J.M., Reddy S., Masters H.R., Gold A., Boldt M., DeFranco E., Evans A.T., Warshak C.R. (2017). Effect of Post-Cesarean Delivery Oral Cephalexin and Metronidazole on Surgical Site Infection among Obese Women: A Randomized Clinical Trial. JAMA.

[6] Pandey N., Cascella M. (2023). Beta-Lactam Antibiotics. StatPearls.

[7] Barbhaiya R.H. (1996). A pharmacokinetic comparison of cefadroxil and cephalexin after administration of 250, 500 and 1000 mg solution doses. Biopharm. Drug Dispos..

[8] Regamey C., Libke R.D., Clarke J.T., Kirby W.M. (1974). Pharmacokinetics of parenteral sodium cephalexin in comparison with cephalothin and cefazolin. Infection.

[9] Plöger G.F., Quizon P.M., Abrahamsson B., Cristofoletti R., Groot D.W., Parr A., Langguth P., Polli J.E., Shah V.P., Tajiri T. (2020). Biowaiver Monographs for Immediate Release Solid Oral Dosage Forms: Cephalexin Monohydrate. J. Pharm. Sci..

[10] Pharmacists A.S.o.H.-S., McEvoy G.K. (2001). AHFS Drug Information, 2001.

[11] Spyker D.A., Thomas B.L., Sande M.A., Bolton W.K. (1978). Pharmacokinetics of cefaclor and cephalexin: Dosage nomograms for impaired renal function. Antimicrob. Agents Chemother..

[12] Sweetman S. (2003). Martindale: The Complete Drug Reference.

[13] Toll L.L., Hurlbut K.M., Poisindex (2003). Poisindex System.

[14] Griffith R.S. (1983). The pharmacology of cephalexin. Postgrad. Med. J..

[15] Williams D., Williams D.A., Lemke T.L. (2002). pKa Values for Some Drugs and Miscellaneous Organic Acids and Bases.

[16] Cephalexin Pregnancy and Breastfeeding Warnings. https://www.drugs.com/pregnancy/cephalexin.html#:~:text=Cephalexin%20Pregnancy%20Warnings,-Animal%20models%20have&text=US%20FDA%20pregnancy%20category%20B,not%20recommended%20unless%20clearly%20needed.

[17] Pichichero M.E. (2006). Cephalosporins can be prescribed safely for penicillin-allergic patients. J. Fam. Pract..

[18] Cephalexin (Rx). https://reference.medscape.com/drug/keflex-cephalexin-342490#5.

[19] Nguyen H.M., Graber C.J. (2020). A Critical Review of Cephalexin and Cefadroxil for the Treatment of Acute Uncomplicated Lower Urinary Tract Infection in the Era of “Bad Bugs, Few Drugs”. Int. J. Antimicrob. Agents.

[20] Liew K.B., Peh K.K., Loh G.O., Tan Y.T. (2014). Three-ways crossover bioequivalence study of cephalexin in healthy Malay volunteers. Drug Dev. Ind. Pharm..

[21] Liu R., Tang A.M., Tan Y.L., Limenta L.M., Lee E.J. (2011). Effects of sodium bicarbonate and ammonium chloride pre-treatments on PEPT2 (SLC15A2) mediated renal clearance of cephalexin in healthy subjects. Drug Metab. Pharmacokinet..

[22] Hassanzadeh M., Fazli-Bazzaz S., Shirazie A. (1996). Relative bioavailability of cephalexin different brands of capsules. Razi J. Med. Sci..

[23] Bataineh Y.A., Aga Q.A.A.K., Al-Jaidi B.A., Al Shorman H.M., Najar A.A., Nair A., Dakkah A.N., Aldhoun M., Daradka H.M., Kurji H.A. (2020). Bioequivalence and Pharmacokinetic Evaluation of Two Batches of Cephalexin Capsules in Healthy Volunteers. J. Pharm. Sci. Res..

[24] Ding Y., Jia Y., Liu W., Lu C., Zhu Y., Yang J., Ding L., Yang L., Wen A. (2012). Chronokinetic study of cefalexin in postprandial and fasting volunteers. Biol. Rhythm. Res..

[25] Lecaillon J.B., Hirtz J.L., Schoeller J.P., Humbert G., Vischer W. (1980). Pharmacokinetic comparison of cefroxadin (CGP 9000) and cephalexin by simultaneous administration to humans. Antimicrob. Agents Chemother..

[26] Nakagawa T., Haginaka J., Yamaoka K., Uno T. (1978). High speed liquid chromatographic determination of cephalexin in human plasma and urine. J. Antibiot..

[27] Lode H., Stahlmann R., Koeppe P. (1979). Comparative pharmacokinetics of cephalexin, cefaclor, cefadroxil, and CGP 9000. Antimicrob. Agents Chemother..

[28] Everts R.J., Gardiner S.J., Zhang M., Begg R., Chambers S.T., Turnidge J., Begg E.J. (2021). Probenecid effects on cephalexin pharmacokinetics and pharmacodynamics in healthy volunteers. J. Infect..

[29] Madaras-Kelly K., Michas P., George M., May M.P., Adejare A. (2004). A randomized crossover study investigating the influence of ranitidine or omeprazole on the pharmacokinetics of cephalexin monohydrate. J. Clin. Pharmacol..

[30] Finkelstein E., Quintiliani R., Lee R., Bracci A., Nightingale C.H. (1978). Pharmacokinetics of oral cephalosporins: Cephradine cephalexin. J. Pharm. Sci..

[31] Ding Y., Jia Y.Y., Li F., Liu W.X., Lu C.T., Zhu Y.R., Yang J., Ding L.K., Yang L., Wen A.D. (2012). The effect of staggered administration of zinc sulfate on the pharmacokinetics of oral cephalexin. Br. J. Clin. Pharmacol..

[32] Mohamed S.S., Mustafa M.A., Ahmed E.A., Algarai n., Alawad Z.A., Ali A.A. (2011). Comparative pharmacokinetics and bioequivalence studies of three oral cephalexin monohydrate formulations. Jordan J. Pharm. Sci..

[33] Welling P.G., Selen A., Pearson J.G., Kwok F., Rogge M.C., Ifan A., Marrero D., Craig W.A., Johnson C.A. (1985). A pharmacokinetic comparison of cephalexin and cefadroxil using HPLC assay procedures. Biopharm. Drug Dispos..

[34] Liu R., Tang A.M., Tan Y.L., Limenta L.M., Lee E.J. (2009). Interethnic differences of PEPT2 (SLC15A2) polymorphism distribution and associations with cephalexin pharmacokinetics in healthy Asian subjects. Eur. J. Clin. Pharmacol..

[35] Ginsburg C.M. (1982). Comparative pharmacokinetics of cefadroxil, cefaclor, cephalexin and cephradine in infants and children. J. Antimicrob. Chemother..

[36] Nahata M.C., Lubin A.H., Visconti J.A. (1984). Cephalexin pharmacokinetics in patients with cystic fibrosis. Dev. Pharmacol. Ther..

[37] Akimoto Y., Komiya M., Kaneko K., Fujii A. (1994). Cefadroxil concentrations in human serum, gingiva, and mandibular bone following a single oral administration. J. Oral Maxillofac. Surg. Off. J. Am. Assoc. Oral Maxillofac. Surg..

[38] Bunke C.M., Aronoff G.R., Brier M.E., Sloan R.S., Luft F.C. (1983). Cefazolin and cephalexin kinetics in continuous ambulatory peritoneal dialysis. Clin. Pharmacol. Ther..

[39] Autmizguine J., Watt K.M., Théorêt Y., Kassir N., Laferrière C., Parent S., Tapiéro B., Ovetchkine P. (2013). Pharmacokinetics and pharmacodynamics of oral cephalexin in children with osteoarticular infections. Pediatr. Infect. Dis. J..

[40] Garrison K.L., Sahin S., Benet L.Z. (2015). Few Drugs Display Flip-Flop Pharmacokinetics and These Are Primarily Associated with Classes 3 and 4 of the BDDCS. J. Pharm. Sci..

[41] Moher D., Liberati A., Tetzlaff J., Altman D.G. (2009). Preferred reporting items for systematic reviews and meta-analyses: The PRISMA statement. Ann. Intern. Med..

[42] Higgins J. (2011). Cochrane Handbook for Systematic Reviews of Interventions. Version 5.1. 0 [Updated March 2011]. The Cochrane Collaboration. https://www.cochrane-handbook.org.

[43] Clark H.D., Wells G.A., Huët C., McAlister F.A., Salmi L.R., Fergusson D., Laupacis A. (1999). Assessing the quality of randomized trials: Reliability of the Jadad scale. Control. Clin. Trials.

[44] Soliman A.B.E., Pawluk S.A., Wilby K.J., Rachid O. (2022). The use of a modified Delphi technique to develop a critical appraisal tool for clinical pharmacokinetic studies. Int. J. Clin. Pharm..

[45] Al-Dirini R.M., Thewlis D., Paul G. (2012). A comprehensive literature review of the pelvis and the lower extremity FE human models under quasi-static conditions. Work.

[46] Higgins J.P., Altman D.G., Gøtzsche P.C., Jüni P., Moher D., Oxman A.D., Savovic J., Schulz K.F., Weeks L., Sterne J.A. (2011). The Cochrane Collaboration’s tool for assessing risk of bias in randomised trials. BMJ (Clin. Res. Ed.).

