# Supplementary material for: A Systematic Review on the Clinical Pharmacokinetics of Cephalexin in Healthy and Diseased Populations
Source: Antibiotics (Basel). 2023 Sep 3;12(9):1402. doi: 10.3390/antibiotics12091402 (PMC10526061; doi:10.3390/antibiotics12091402)
Supplement: Supplementary file 1 [file antibiotics-12-01402-s001.zip › antibiotics-2525041-supplementary.pdf]

Supplementary Information

A Systematic Review on the Clinical Pharmacokinetics of Cephalexin in Healthy and Diseased Populations

Mohammed Kanan [1,*], Shahd Atif [2], Faisal Mohammed [3], Yara Balahmar [4], Yasir Adawi [5], Revan AlSaleem [6], Ahmed Farhan [7], Manayer Alghoribi [8], Saud Mohammed [6], Raghad Alshanbari [9], Malak Fahad [10], Rana Kallab [11], Reem Mohammed [6], Dimah Alassaf [12] and Ashwag Hazza [13]

[1] Department of Clinical Pharmacy, King Fahad Medical City, Riyadh 12211, Saudi Arabia
[2] Al Iman General Hospital, Riyadh 12211, Saudi Arabia; shahad.atif1@gmail.com
[3] Department of Clinical Pharmacy, College of Pharmacy, Umm Al-Qura University, Makkah 24211, Saudi Arabia; faisalpo1029@gmail.com
[4] Department of Clinical Pharmacy, College of Pharmacy, Ibn Sina College, Jeddah, Saudi Arabia; yarabalahmar@hotmail.com
[5] Department of Clinical Pharmacy, Jazan University, Jazan 85534, Saudi Arabia; yasir50adawi@gmail.com
[6] College of Pharmacy, King Khalid University, Abha 61421, Saudi Arabia; refanq21@gmail.com (R.A.) sauod1128@gmail.com (S.M.); reemalshehri.m@gmail.com (R.M.)
[7] Department of Pharmacy, Prince Sultan Military Medical City, Riyadh 12211, Saudi Arabia; ahmedaldafiri9@gmail.com
[8] Al-Nahda General Hospital, Private Healthcare Sector, Taif City 26575, Saudi Arabia; manayer.g@hotmail.com
[9] Department of Pharmacy, Erfan and Bagedo General Hospital, Jeddah 22230, Saudi Arabia; alshanbariraghad.1996@gmail.com AlshanbariRaghad.1996@gmail.com
[10] Department of Clinical Pharmacy, Northern Borders University, Rafha 91911, Saudi Arabia; malak.fahad.f@gmail.com
[11] Department of Pharmacy, Aldawaa Pharmacy, Arar 73551, Saudi Arabia; ranakallab@hotmail.com
[12] College of Medicine, Princess Noura University, Riyadh 12211, Saudi Arabia; dimaalassaff@gmail.com
[13] Department of Pharmacy, Altaawin Medical Clinics, Alkharj 16443, Saudi Arabia; ashwag.alsubaie0@gmail.com
* Correspondence: ii_kanan101@outlook.com

Supplementary Table S1: Screening of articles based on title, abstract, presence of animals and language

Sr. No.	Title of Article	Exclusion basis
1	(1971). "Cephalexin (Keflex)." Med Lett Drugs Ther 13(7): 25-26.	Abstract
2	(1991). "Swedish Study Group. A randomized multicenter trial to compare the influence of cefaclor and amoxycillin on the colonization resistance of the digestive tract in patients with lower respiratory tract infection." Infection 19(4): 208-215.	Title
3	(2000). "Antimicrobial treatment guidelines for acute bacterial rhinosinusitis: Sinus and Allergy Health Partnership *." Otolaryngology - Head and Neck Surgery 123(1, Part 2): S4-S31.	Title
4	(2001). "Antimicrobial treatment guidelines for acute bacterial rhinosinusitis." Disease-a-Month 47(11): 537-585.	Title
5	(2004). Chapter 4 Mechanisms of immunotoxic effects. Immunotoxicology of Drugs and Chemicals: an Experimental and Clinical Approach. J. Descotes, Elsevier. 1: 127-162.	Title
6	(2010). Oral Drug Absorption: Prediction And Assessment	Title
7	(2012). "APhA2012 abstracts of contributed papers." Journal of the American Pharmacists Association 52(2): 200-284.	Title
8	(2021). "Abstracts from the North American Neuromodulation Society's 2021 Virtual Meeting, January 15–16, 2021." Neuromodulation: Technology at the Neural Interface 24(4): e1-e276.	Title
9	Abduljalil, K. M. A. (2010). Advanced population pharmacokinetic modelling to quantify selected characteristics of drugs, Universitäts- und Landesbibliothek Bonn.	Title
10	Abdulwahab, H. G., et al. (2020). "Novel thiobarbiturates as potent urease inhibitors with potential antibacterial activity: Design, synthesis, radiolabeling and biodistribution study." Bioorg Med Chem 28(23): 115759.	Title
11	Actor, P., et al. (1976). "Cefatrizine (SK&F 60771), a new oral cephalosporin: serum levels and urinary recovery in humans after oral or intramuscular administration; comparative study with cephalexin and cefazolin." Antimicrobagents chemother 9(5): 800-803.	Abstract
12	Adam, D., et al. (1978). "Interference of the tissue concentration of antibiotics with a salidiuretic. Behaviour of cephradine and cephalothin in brain tissue after additional administration of furosemide (author's transl)." Klinische Wochenschrift 56(5): 247-251.	Title
13	Agatonovic-Kustrin, S., et al. (2002). "Molecular descriptors that influence the amount of drugs transfer into human breast milk." J Pharm Biomed Anal 29(1): 103-119.	Title

14	Agoram, B., et al. (2001). "Predicting the impact of physiological and biochemical processes on oral drug bioavailability." Advanced Drug Delivery Reviews 50: S41-S67.	Title
15	Ahmadi, S., et al. (2016). "Comparison of single dose of cefazolion versus single dose of cefazolin plus seven days cephalexin prophylactic therapy in postoperative infections after elective cesarean section and immediate adverse effect of antibiotic on infants." Iranian journal of obstetrics, gynecology and infertility 19(15): 1-7.	Abstract
16	Ahrens, T. and K. G. Naber (1983). "Activity of cefroxadine and cephalexin in urinary tract infections: a double-blind comparative study." Infection 11(1): 25-30.	Abstract
17	Akimoto, Y., et al. (1998). "Cefaclor concentration in radicular granuloma after a single oral administration." Gen Pharmacol 31(2): 283-285.	Title
18	Akimoto, Y., et al. (1994). "Cefadroxil concentrations in human serum, gingiva, and mandibular bone following a single oral administration." J Oral Maxillofac Surg 52(4): 397-400; discussion 400-391.	Title
19	Akimoto, Y., et al. (1992). "Cefaclor concentrations in human serum, gingiva, mandibular bone, and dental follicle following a single oral administration." Gen Pharmacol 23(4): 639-642.	Title
20	Akimoto, Y., et al. (1996). "Cefaclor concentration in pus from abscess caused by odontogenic infection after a single oral administration." Gen Pharmacol 27(1): 177-179.	Title
21	Ali, I., et al. (2023). "Advancement in oral pharmacokinetics of an antibiotic Cefixime using amphiphilic 4-armed macrocyclic surfactant based niosomes." Arabian Journal of Chemistry 16(8): 104958.	Title
22	Allen, J. G. and L. J. Lees (1980). "Pharmacokinetics of alafosfalin, alone and in combination with cephalexin, in humans." Antimicrob Agents Chemother 17(6): 973-979.	Abstract
23	Altergott, C., et al. (2008). "Pediatric fingertip injuries: do prophylactic antibiotics alter infection rates?" Pediatric emergency care 24(3): 148-152.	Title
24	Aman, S. F., et al. (2010). "Studies of food drug interactions." Pak J Pharm Sci 23.	Title
25	Amidon, G. L., et al. (1988). "Estimating human oral fraction dose absorbed: a correlation using rat intestinal membrane permeability for passive and carrier-mediated compounds." Pharm Res 5(10): 651-654.	Animal
26	Anand, B. S., et al. (2004). "Pharmacokinetics of novel dipeptide ester prodrugs of acyclovir after oral administration: intestinal absorption and liver metabolism." Journal of Pharmacology Experimental Therapeutics 311(2): 659-667.	Title

27	Anand, B. S., et al. (2003). "Interactions of the dipeptide ester prodrugs of acyclovir with the intestinal oligopeptide transporter: competitive inhibition of glycylsarcosine transport in human intestinal cell line-Caco-2." J Pharmacol Exp Ther 304(2): 781-791.	Title
28	Andersson, K. E. (1978). "On the pharmacokinetics of cephalosporin antibiotics." Scand J Infect Dis Suppl(13): 37-46.	Abstract
29	Andreas, C. J., et al. (2017). "Mechanistic investigation of the negative food effect of modified release zolpidem." European Journal of Pharmaceutical Sciences 102: 284-298.	Title
30	Andriole, V. T. (1978). "Pharmacokinetics of cephalosporins in patients with normal or reduced renal function." J Infect Dis 137 Suppl: S88-s99.	Abstract
31	Arakawa, H., et al. (2016). "Possible interaction of quinolone antibiotics with peptide transporter 1 in oral absorption of peptide-mimetic drugs." Biopharm Drug Dispos 37(1): 39-45.	Title
32	Arakawa, H., et al. (2014). "Evaluation of a thiodipeptide, L-phenylalanyl-Ψ[CS-N]-L-alanine, as a novel probe for peptide transporter 1." Drug Metab Pharmacokinet 29(6): 470-474.	Title
33	Arakawa, S., et al. (1990). "A double-blind comparative study of S 6472 (a long-acting cefaclor) versus a conventional cefaclor preparation in complicated urinary tract infections." Japanese journal of antibiotics 43(11): 1873-1892.	Title
34	Arguello, F., et al. (1998). "Flavopiridol Induces Apoptosis of Normal Lymphoid Cells, Causes Immunosuppression, and Has Potent Antitumor Activity In Vivo Against Human Leukemia and Lymphoma Xenografts." Blood 91(7): 2482-2490.	Title
35	Avery, R. K. (2010). "Infectious Disease Following Kidney Transplant: Core Curriculum 2010." American Journal of Kidney Diseases 55(4): 755-771.	Title
36	Baba, S., et al. (1983). "The double-blind trial of cefroxadine and cephalexin in the treatment of acute suppurative otitis media and acute exacerbation of chronic suppurative otitis media." Japanese journal of antibiotics 36(9): 2595-2634.	Abstract
37	Bachmann, K., et al. (1986). "Impact of cefaclor on the pharmacokinetics of theophylline." Ther Drug Monit 8(2): 151-154.	Title
38	Badenoch, P. R., et al. (1986). "Effect of inflammation on antibiotic penetration into the anterior segment of the rat eye." Invest Ophthalmol Vis Sci 27(6): 958-965.	Animal
39	Badhan, R. K. S., et al. (2014). "Development of a physiologically-based pharmacokinetic model of the rat central nervous system." Pharmaceutics 6(1): 97-136.	Animal
40	Bailey, A., et al. (1970). "Cephalexin--a new oral antibiotic." Practitioner 205(230): 791-795.	Abstract

41	Ball, P., et al. (2004). "Safety profile of oral and intravenous moxifloxacin: Cumulative data from clinical trials and postmarketing studies." Clin Ther 26(7): 940-950.	Title
42	Ballantyne, F. N. (1985). "Comparative efficacy of cefadroxil and cefaclor in the treatment of skin and soft-tissue infections." Clin Ther 7(4): 487-491.	Title
43	Bamfo, N. O., et al. (2021). "Examination of urinary excretion of unchanged drug in humans and preclinical animal models: increasing the predictability of poor metabolism in humans." Pharm Res 38: 1139-1156.	Title
44	Bandak, S. I., et al. (1999). "Cefaclor af versus amoxycillin/clavulanate in acute bacterial exacerbations of chronic bronchitis: a randomised multicentre study." Int J Clin Pract 53(8): 578-583.	Title
45	Barberán, J., et al. (2007). "Update on osteo-articular infections and severe skin and soft tissue infections." Enfermedades Infecciosas y Microbiología Clínica 25: 28-36.	Title
46	Barbhaiya, R. H., et al. (1990). "Comparison of the effects of food on the pharmacokinetics of cefprozil and cefaclor." Antimicrob Agents Chemother 34(6): 1210-1213.	Title
47	Barbhaiya, R. H., et al. (1990). "Phase I study of multiple-dose cefprozil and comparison with cefaclor." Antimicrob Agents Chemother 34(6): 1198-1203.	Title
48	Barbhaiya, R. H., et al. (1990). "Comparison of cefprozil and cefaclor pharmacokinetics and tissue penetration." Antimicrob Agents Chemother 34(6): 1204-1209.	Title
49	Barkworth, M. F., et al. (1991). "[The biological availability of cefadroxil given simultaneously with N-acetylcysteine]." Arzneimittelforschung 41(8): 839-843.	Title
50	Barreca, T., et al. (1984). "Therapeutic experience with cofosfolactamines: clinical and bacteriological results of two multicentre double-blind studies." Drugs Exp Clin Res 10(1): 55-62.	Title
51	Barrios, S., et al. (1975). "Bioavailability of cephalexin after intramuscular injection of its lysine salt." J Pharm Pharmacol 27(9): 711-712.	Abstract
52	Basha, M., et al. (2018). "A potential antibacterial wound dressing of cefadroxil chitosan nanoparticles in situ gel: Fabrication, in vitro optimization and in vivo evaluation." Int J Pharm 544(1): 129-140.	Title
53	Bathini, L., et al. (2019). "Clinical Outcomes of Failing to Dose-Reduce Cephalosporin Antibiotics in Older Adults with CKD." Clin J Am Soc Nephrol 14(2): 197-205.	Title

54	Bauer, P. and A. Windorfer (1981). "[Cefadroxil - fewer single administrations in better bioavailability]." ZFA (Stuttgart) 57(31): 2174-2177.	Title
55	Bedenic, B., et al. (2006). "Urinary bactericidal activity of oral antibiotics against common urinary tract pathogens in an ex vivo model." Chemotherapy 52(6): 293-297.	Title
56	Belousov, I., et al. (1998). "Selection of antibacterial therapy for treatment of infections in elderly patients." Antibiot Khimioter 43(10): 19-23.	Title
57	Bennett, P., et al. (1996). Monographs on individual drugs. Drugs and Human Lactation, Elsevier: 75-532.	Title
58	Bennett, W. M., et al. (1983). "Drug Prescribing in Renal Failure: Dosing Guidelines for Adults." American Journal of Kidney Diseases 3(3): 155-193.	Title
59	Bergan, T. (1980). "Pharmacokinetics of a new cephalosporin, CGP 9000 (cefroxadine), in healthy volunteers." Chemotherapy 26(4): 225-230.	Title
60	Bergan, T. (1987). "Pharmacokinetic properties of the cephalosporins." Drugs 34 Suppl 2: 89-104.	Abstract
61	Bergan, T., et al. (1983). "Relationship between pharmacokinetics and bioavailability of cefroxadine (CGP 9000) and renal function." Chemotherapy 29(3): 163-173.	Title
62	Bergan, T., et al. (1970). "Human pharmacokinetics of cephalexin." Pharmacology 4(5): 264-272.	Abstract
63	Bergogne-Berezin, E., et al. (1978). "Pharmacokinetic study of beta-lactam antibiotics in bronchial secretions." Scand J Infect Dis Suppl(14): 267-272.	Title
64	Berlioz, F., et al. (2000). "Chronic nifedipine dosing enhances cephalexin bioavailability and intestinal absorption in conscious rats." Drug Metab Dispos 28(11): 1267-1269.	Animal
65	Berlioz, F., et al. (2000). "alpha(2)-adrenergic receptors stimulate oligopeptide transport in a human intestinal cell line." J Pharmacol Exp Ther 294(2): 466-472.	Title
66	Bersani, C., et al. (1986). "[Pharmacokinetics of cofosfolactamines in subjects with impaired kidney function]." G Ital Chemioter 33(2-3): 121-128.	Title
67	Beumer, H. M. and J. Veldkamp (1982). "Oral cefaclor for treatment of bronchitis--a comparative double-blind study versus amoxycillin." Int J Clin Pharmacol Ther Toxicol 20(3): 113-117.	Title

68	Beyssac, E., et al. (1991). "Comparison of continuous, constant rate enteral tube feeding in supine patients to bolus food intake in ambulatory, healthy subjects regarding bioavailability of perorally administered cefroxadine." Methods Find Exp Clin Pharmacol 13(9): 637-642.	Title
69	Beyssac, E., et al. (1991). "Peroral absorption of cefroxadine in patients within the first day after severe trauma: comparison to cefroxadine pharmacokinetics in fasted, healthy volunteers." Methods Find Exp Clin Pharmacol 13(8): 565-572.	Title
70	Bhavnani, S. M., et al. (2004). "Pharmacokinetics, safety, and tolerability of ascending single intravenous doses of oritavancin administered to healthy human subjects." Diagnostic microbiology infectious disease 50(2): 95-102.	Title
71	Bhutani, U., et al. (2021). "Oral Drug Delivery: Conventional to Long Acting New-Age Designs." European Journal of Pharmaceutics and Biopharmaceutics 162: 23-42.	Title
72	Billig, H., et al. (1990). "The disposition of valpromide in rats and the isolated perfused rat liver." Drug metabolism disposition 18(2): 238-244.	Animal
73	Bins, J. W. and H. Mattie (1988). "Saturation of the tubular excretion of beta-lactam antibiotics." Br J Clin Pharmacol 25(1): 41-50.	Title
74	Bjerre, C., et al. (1996). "Bioavailability of the sedative propiomazine after nasal administration in rats." Int J Pharm 144(2): 217-224.	Animal
75	Blondeau, J. M. (2000). "A review of clinical trials with fluoroquinolones with an emphasis on new agents." Expert opinion on investigational drugs 9(2): 383-413.	Title
76	Blumer, J. L. (2005). "Evolution of a new drug formulation: the rationale for high-dose, short-course therapy with azithromycin." Int J Antimicrob Agents 26: S143-S147.	Title
77	Bolding, O. T. (1980). "Comparison of the efficacy and safety of cefadroxil and cephalexin in treating acute urinary tract infections in women." Journal of international medical research 8(Suppl. 1): 34-39.	Abstract
78	Boobis, A. R. and P. J. Lewis (1983). "Pharmacokinetics in pregnancy." Clinical Pharmacology in Obstetrics.	Title
79	Boothman, R., et al. (1973). "Absorption and excretion of cephalexin by the newborn infant." Arch Dis Child 48(2): 147-150.	Abstract
80	Bottaro, G., et al. (2012). "5 days Cefaclor vs. 10 days amoxicillin/clavulanate in the treatment of childhood streptococcal pharyngitis. Data from a randomized clinical trial." Minerva pediatrica 64(3): 341-346.	Title
81	Bottoni, P. and S. Caroli (2018). "Presence of residues and metabolites of pharmaceuticals in environmental compartments, food commodities and workplaces: A review spanning the three-year period 2014–2016." Microchemical Journal 136: 2-24.	Title

82	Bourdet, D. L. (2005). Novel mechanisms in the intestinal absorption of hydrophilic cationic drugs, The University of North Carolina at Chapel Hill.	Title
83	Bouza, E. and A. Burillo (2010). "Oritavancin: a novel lipoglycopeptide active against Gram-positive pathogens including multiresistant strains." Int J Antimicrob Agents 36(5): 401-407.	Title
84	Bradley, J. S. (2023). "What Is the Appropriate Dose, Route, and Duration of Antibiotic Therapy for Pediatric Acute Hematogenous Osteomyelitis (AHO)? I Wish I Knew." J Pediatric Infect Dis Soc 12(2): 61-63.	Title
85	Bradley, J. S., et al. (2019). "Safety and efficacy of oral and/or intravenous tedizolid phosphate (TZD) in adolescents with acute bacterial skin and skin structure tissue infections (ABSSSI)." Open forum infectious diseases 6: S230-S231.	Title
86	Bratlid, D. and T. Bergan (1976). "Displacement of albumin-bound antimicrobial agents by bilirubin." Pharmacology 14(5): 464-472.	Title
87	Bretschneider, B., et al. (1999). "Intestinal transport of beta-lactam antibiotics: analysis of the affinity at the H+/peptide symporter (PEPT1), the uptake into Caco-2 cell monolayers and the transepithelial flux." Pharm Res 16(1): 55-61.	Title
88	Brisson, A. M. and J. B. Fourtillan (1982). "Pharmacokinetic study of cefadroxil following single and repeated doses." J Antimicrob Chemother 10 Suppl B: 11-15.	Title
89	Brockmann, W. and M. Badr (2010). "Chronic Kidney Disease: Pharmacological considerations for the dentist." The Journal of the American Dental Association 141(11): 1330-1339.	Title
90	Brogard, J. M. and F. Comte (1982). "Pharmacokinetics of the new cephalosporins." Antibiot Chemother (1971) 31: 145-210.	Abstract
91	Brogard, J. M., et al. (1978). "Pharmacokinetics of cephalosporin antibiotics." Antibiot Chemother (1971) 25: 123-162.	Abstract
92	Brogden, R. N., et al. (1982). "Trimethoprim: a review of its antibacterial activity, pharmacokinetics and therapeutic use in urinary tract infections." Drugs 23(6): 405-430.	Title
93	Brooks, S. and A. R. Dent (1984). "Comparison of bone levels after intramuscular administration of cephradine ('Velosef') or flucloxacillin/ampicillin in hip replacement." Pharmatherapeutica 3(10): 642-649.	Title
94	Brumfitt, W. and J. M. Hamilton-Miller (1999). "Cefaclor into the millennium." J Chemother 11(3): 163-178.	Title
95	Bucko, A. D., et al. (2002). "Randomized, double-blind, multicenter comparison of oral cefditoren 200 or 400 mg BID with either cefuroxime 250 mg BID or cefadroxil 500 mg BID for the treatment of uncomplicated skin and skin-structure infections." Clin Ther 24(7): 1134-1147.	Title

96	Buckpitt, A. R. and M. R. Boyd (1980). "A sensitive method for determination of 5-fluorouracil and 5-fluoro-2′-deoxyuridine in human plasma by high-pressure liquid chromatography." Analytical Biochemistry 106(2): 432-437.	Title
97	Bundgaard, H. and G. J. Friis (1992). "Prodrugs of peptides. 16. Isocyclosporin A as a potential prodrug of cyclosporin A." Int J Pharm 82(1): 85-90.	Title
98	Bunke, C. M., et al. (1983). "Pharmacokinetics of common antibiotics used in continuous ambulatory peritoneal dialysis." Am J Kidney Dis 3(2): 114-117.	Title
99	Bustrack, J. A., et al. (1980). "A comparative pharmacokinetic and safety study of an investigational oral cephalosporin, RMI 19,592." Current therapeutic research - clinical and experimental 28(2): 208-217.	Abstract
100	Butcher, R. H., et al. (1972). "Blood and urine levels of cephalexin in patients with impaired renal function." Med J Aust 2(23): 1282-1284.	Abstract
101	Buur, A., et al. (1988). "Prodrugs of propranolol: hydrolysis and intramolecular aminolysis of various propranolol esters and an oxazolidin-2-one derivative." Int J Pharm 42(1): 51-60.	Title
102	Byun, S.-Y., et al. (2016). "Pharmacokinetic study of meropenem in healthy beagle dogs receiving intermittent hemodialysis." Journal of Veterinary Pharmacology Therapeutics 39(6): 560-565.	Animal
103	Cadórniga, R., et al. (1990). "Pharmacokinetics of cefroxadine after infusion to healthy volunteers." Int J Clin Pharmacol Ther Toxicol 28(10): 435-439.	Title
104	Cain, T. J., et al. (1987). "Bone levels of cephradine and cefuroxime after intravenous administration in patients undergoing total hip replacement." International orthopaedics 11(1): 61-63.	Title
105	Campanacci, L., et al. (1975). "[Antibiotic therapy and renal insufficiency. Pharmacokinetics of pivampicillin, cephaloridin and streptomycin in chronic uremic patients undergoing conservative or hemodialytic treatment]." Minerva Med 66(68): 3547-3565.	Title
106	Campbell, D. B. (1997). Chirality and kinetics. Pharmacochemistry Library. F. Awouters and K. C. Waugh, Elsevier. 28: 45-60.	Title
107	Cappelletty, D. M. and M. J. Rybak (1996). "Bactericidal activities of cefprozil, penicillin, cefaclor, cefixime, and loracarbef against penicillin-susceptible and -resistant Streptococcus pneumoniae in an in vitro pharmacodynamic infection model." Antimicrob Agents Chemother 40(5): 1148-1152.	Title
108	Caramatti, C., et al. (1980). "[Clinical evaluation of use of combinations of beta-lactamines in anti-infective therapy]." Ateneo Parmense Acta Biomed 51(1): 57-66.	Title

109	Carli, S., et al. (1999). "Absorption kinetics and bioavailability of cephalexin in the dog after oral and intramuscular administration." J Vet Pharmacol Ther 22(5): 308-313.	Animal
110	Carr, R. A. (1995). "Pharmacokinetics of Sotalol enantiomers." Clin Pharmacol.	Title
111	Carsenti-Etesse, H., et al. (1998). "Pharmacokinetic parameters and killing rates in serum of volunteers receiving amoxicillin, cefadroxil or cefixime alone or associated with niflumic acid or paracetamol." Eur J Drug Metab Pharmacokinet 23(3): 357-366.	Title
112	Casewell, M. W. and S. G. Bragman (1987). "The in-vitro activity of cefadroxil, and the interpretation of disc-susceptibility testing." J Antimicrob Chemother 19(5): 597-603.	Title
113	Castro, M. (1986). "A comparative study of cefadroxil and co-trimoxazole in patients with lower respiratory tract infections." Drugs 32 Suppl 3: 50-56.	Title
114	Cattrall, J. W. S., et al. (2019). "A pharmacokinetic-pharmacodynamic assessment of oral antibiotics for pyelonephritis." Eur J Clin Microbiol Infect Dis 38(12): 2311-2321.	Title
115	Cayen, M. N. (1985). "Disposition, metabolism and pharmacokinetics of antihyperlipidemic agents in laboratory animals and man." Pharmacology & Therapeutics 29(2): 157-204.	Title
116	Cazzola, M., et al. (2000). "Interrelationship between the pharmacokinetics and pharmacodynamics of cefaclor advanced formulation in patients with acute exacerbation of chronic bronchitis." J Chemother 12(3): 216-222.	Title
117	Chang, Q., et al. (2009). "Studies on the influence of esterase inhibitor to the pharmacokinetic profiles of oseltamivir and oseltamivir carboxylate in rats using an improved LC/MS/MS method." Biomed Chromatogr 23(8): 852-857.	Animal
118	Charles, B. and S. Chulavatnatol (1993). "Simple analysis of amoxycillin in plasma by high performance liquid chromatography with internal standardization and ultraviolet detection." Biomed Chromatogr 7(4): 204-207.	Title
119	Chavada, V. D., et al. (2020). "Citrate/melamine functionalized gold nanoparticles for concurrent determination of allopurinol and its major metabolite, oxypurinol in plasma and pharmaceuticals." Journal of Industrial and Engineering Chemistry 84: 141-149.	Title
120	Chen, J., et al. (2012). "Bioequivalence studies of 2 oral cefaclor capsule formulations in chinese healthy subjects." Arzneimittelforschung 62(3): 134-137.	Title
121	Chen, L. and X. Chen (2012). "Results of molecular docking as descriptors to predict human serum albumin binding affinity." Journal of Molecular Graphics and Modelling 33: 35-43.	Title

122	Chen, M. L. (1992). "An alternative approach for assessment of rate of absorption in bioequivalence studies." Pharm Res 9(11): 1380-1385.	Title
123	Chen, X. (2000). Pharmacokinetically guided chemotherapy for biliary tract infection, The Ohio State University.	Title
124	Chen, X. (2017). Influence of PEPT2 on the Regional Distribution Kinetics of Cefadroxil in Brain Using Intracerebral Microdialysis in Rats, Wildtype and PEPT2 Knockout mice.	Animal
125	Chen, X., et al. (2017). "Influence of peptide transporter 2 (PEPT2) on the distribution of cefadroxil in mouse brain: a microdialysis study." Biochem Pharmacol 131: 89-97.	Animal
126	Chen, X., et al. (2012). "Investigation of dried blood spot card-induced interferences in liquid chromatography/mass spectrometry." J Pharm Biomed Anal 61: 30-37.	Title
127	Chen, X., et al. (2003). "Determination of cefaclor in human plasma by a sensitive and specific liquid chromatographic-tandem mass spectrometric method." J Chromatogr B Analyt Technol Biomed Life Sci 784(1): 17-24.	Title
128	Cheng, C.-L. and C.-H. Chou (2001). "Determination of metformin in human plasma by high-performance liquid chromatography with spectrophotometric detection." Journal of Chromatography B: Biomedical Sciences and Applications 762(1): 51-58.	Title
129	Chew, M. L., et al. (2008). "Anticholinergic activity of 107 medications commonly used by older adults." J Am Geriatr Soc 56(7): 1333-1341.	Title
130	Chi Chan, A., et al. (1999). "Efficacy and safety of cefalexin-bromhexin vs amoxicillin in acute bronchitis and chronic bronchitis acute exacerbations." Investigacion medica internacional 26(1): 19-24.	Abstract
131	Chien, J. Naine: PPD.	Title
132	Chin, K. C., et al. (1981). "A pharmacological study of cefaclor in the newborn infant." Curr Med Res Opin 7(3): 168-170.	Title
133	Chin, P. K. L., et al. (2023). "One giant leap for cephalexin dosing, one small step for antimicrobial stewardship." Cjem 25(1): 7-8.	Abstract
134	Choi, S. J., et al. (2009). "Rapid and simple method for determination of cephradine in human plasma using liquid chromatography-tandem mass spectrometry (LC-MS/MS): application to the bioequivalence study." J Chromatogr B Analyt Technol Biomed Life Sci 877(31): 4059-4064.	Title
135	Chougule, D. D. and N. S. Naikwade (2015). "Pharmacokinetic and bioequivalence evaluation of two generic brands of cephalexin: a single-dose, randomized, open-label, parallel study." Latin american journal of pharmacy 34(7): 1459-1463.	Abstract
136	Chow, M., et al. (1979). "Pharmacokinetics of high-dose oral cephalosporins." J Clin Pharmacol 19(4): 185-194.	Abstract

137	Chowdhry, B. Z., et al. (1983). "Analysis of drugs by microcalorimetry: Isothermal power-conduction calorimetry and thermometric titrimetry." Talanta 30(4): 209-243.	Title
138	Christenson, J. C., et al. (1991). "Comparative efficacy and safety of cefprozil and cefaclor in the treatment of acute uncomplicated urinary tract infections." J Antimicrob Chemother 28(4): 581-586.	Title
139	Christenson, J. C., et al. (1991). "Comparative efficacy and safety of cefprozil (BMY-28100) and cefaclor in the treatment of acute group A beta-hemolytic streptococcal pharyngitis." Antimicrob Agents Chemother 35(6): 1127-1130.	Title
140	Chu, X. Y., et al. (2001). "Correlation between epithelial cell permeability of cephalexin and expression of intestinal oligopeptide transporter." J Pharmacol Exp Ther 299(2): 575-582.	Abstract
141	Cole, P. (1997). "Pharmacologic and clinical comparison of cefaclor in immediate-release capsule and extended-release tablet forms." Clin Ther 19(4): 617-625; discussion 603.	Title
142	Condon, R. E. and D. H. Wittmann (1991). "The use of antibiotics in general surgery." Current Problems in Surgery 28(12): 807-907.	Title
143	Cooper, R. D. G. (1992). "The carbacephems: A new beta-lactam antibiotic class." The American Journal of Medicine 92(6, Supplement 1): S2-S6.	Title
144	Corson, A. H., et al. (2016). "Why isn't cefadroxil used more often?" Am J Health Syst Pharm 73(11): 754-755.	Title
145	Couet, W., et al. (1991). "Pharmacokinetics of oral cefatrizine in patients with impaired renal function." Int J Clin Pharmacol Ther Toxicol 29(6): 213-217.	Title
146	Couet, W., et al. (1988). "[Pharmacokinetics of cefatrizine administered in repeated doses]." Pathol Biol (Paris) 36(5): 513-516.	Title
147	Couet, W. R., et al. (1991). "Theoretical model for both saturable rate and extent of absorption: simulations of cefatrizine data." J Pharmacokinet Biopharm 19(3): 271-285.	Title
148	Covitz, K. M., et al. (1996). "Human dipeptide transporter, hPEPT1, stably transfected into Chinese hamster ovary cells." Pharm Res 13(11): 1631-1634.	Title
149	Craft, J. C., et al. (1987). "Malabsorption of oral antibiotics in humans and rats with giardiasis." Pediatr Infect Dis J 6(9): 832-836.	Title
150	Crandon, J. and D. P. Nicolau (2008). "Oritavancin: a potential weapon in the battle against serious Gram-positive pathogens." Future Microbiol 3(3): 251-263.	Title
151	Creasey, W. A., et al. (1984). "Pharmacokinetic interaction of aztreonam with other antibiotics." J Clin Pharmacol 24(4): 174-180.	Title

152	Crespi, C. L., et al. (1996). "Development of Caco-2 cells expressing high levels of cDNA-derived cytochrome P4503A4." Pharm Res 13(11): 1635-1641.	Title
153	Crowe, H. M. and R. Quintiliani (1995). "Antibiotic formulary selection." Medical Clinics of North America 79(3): 463-476.	Title
154	Cui, H., et al. (2016). "Development of a drug-in-adhesive patch combining ion pair and chemical enhancer strategy for transdermal delivery of zaltoprofen: pharmacokinetic, pharmacodynamic and in vitro/in vivo correlation evaluation." Drug Delivery 23(9): 3461-3470.	Title
155	Cummings, D. M. and K. M. Uttech (1990). "Antibiotics for Common Infections in the Elderly." Primary Care: Clinics in Office Practice 17(4): 883-903.	Title
156	Curtin, C. D., et al. (2003). "Efficacy of cephalexin two vs. three times daily vs. cefadroxil once daily for streptococcal tonsillopharyngitis." Clinical pediatrics 42(6): 519-526.	Full text
157	Cutler, R. (1980). "Cefadroxil kinetics in patients with impaired renal function." J Int Med Res 8(Suppl 1): 17-20.	Title
158	Dagan, R., et al. (2000). "Bacteriologic efficacies of oral azithromycin and oral cefaclor in treatment of acute otitis media in infants and young children." Antimicrob Agents Chemother 44(1): 43-50.	Title
159	Dahlgren, D., et al. (2015). "Direct in vivo human intestinal permeability (Peff) determined with different clinical perfusion and intubation methods." Journal of pharmaceutical sciences and research 104(9): 2702-2726.	Title
160	Dahlin, M. (2000). Nasal administration of compounds active in the central nervous system: exploring the olfactory system, Acta Universitatis Upsaliensis.	Title
161	Dallmann, A., et al. (2017). "Physiologically Based Pharmacokinetic Modeling of Renally Cleared Drugs in Pregnant Women." Clin Pharmacokinet 56(12): 1525-1541.	Title
162	Dantzig, A. H. and L. Bergin (1988). "Carrier-mediated uptake of cephalexin in human intestinal cells." Biochem Biophys Res Commun 155(2): 1082-1087.	Abstract
163	Dantzig, A. H. and L. Bergin (1990). "Uptake of the cephalosporin, cephalexin, by a dipeptide transport carrier in the human intestinal cell line, Caco-2." Biochim Biophys Acta 1027(3): 211-217.	Abstract
164	Dantzig, A. H., et al. (1994). "Transport mechanisms responsible for the absorption of loracarbef, cefixime, and cefuroxime axetil into human intestinal Caco-2 cells." Biochim Biophys Acta 1191(1): 7-13.	Title

165	Darmstadt, G. L. (1998). "ANTIBIOTICS IN THE MANAGEMENT OF PEDIATRIC SKIN DISEASE." Dermatologic Clinics 16(3): 509-525.	Title
166	Davies, A. J., et al. (1986). "Comparative pharmacokinetics of cefamandole, cefuroxime and cephradine during total hip replacement." J Antimicrob Chemother 17(5): 637-640.	Title
167	Davies, B. J., et al. (2006). "Determination of the 4-monohydroxy metabolites of perhexiline in human plasma, urine and liver microsomes by liquid chromatography." Journal of Chromatography B 843(2): 302-309.	Title
168	Davies, J. A. and J. M. Holt (1975). "Absorption of cephalexin in diseased and aged subjects." J Antimicrob Chemother 1(3 Suppl): 69-70.	Abstract
169	Davies, J. A., et al. (1970). "Absorption of cephalexin from the gastrointestinal tract in disease subjects." Postgrad Med J: Suppl:16-19.	Abstract
170	Davis, C. M., et al. (2016). "Prevalence of Surgical Site Infections Following Orthognathic Surgery: a Double-Blind, Randomized Controlled Trial on a 3-Day Versus 1-Day Postoperative Antibiotic Regimen." Journal of oral and maxillofacial surgery. (no pagination), 2016 Date of Publication: August 07.	Title
171	Davis, C. M., et al. (2017). "Prevalence of Surgical Site Infections Following Orthognathic Surgery: a Double-Blind, Randomized Controlled Trial on a 3-Day Versus 1-Day Postoperative Antibiotic Regimen." Journal of oral and maxillofacial surgery 75(4): 796-804.	Title
172	Davis, G. A. and M. H. H. Chandler (1996). "DRUG THERAPY AND DRUG INTERACTIONS." Oral and Maxillofacial Surgery Clinics of North America 8(2): 245-263.	Title
173	Davis, J. L., et al. (2006). "Mucosal permeability of water-soluble drugs in the equine jejunum: a preliminary investigation." J Vet Pharmacol Ther 29(5): 379-385.	Title
174	Dawborn, J. K. (1974). "Renal failure and the action of drugs." Prog Biochem Pharmacol 9: 206-220.	Title
175	de Fijter, C. W., et al. (1989). "Are intracellularly penetrating antibiotics warranted in CAPD-related peritonitis?" Adv Perit Dial 5: 124-127.	Title
176	de, l., et al. (2002). "Comparative target site pharmacokinetics of immediate- and modified-release formulations of cefaclor in humans." J Clin Pharmacol 42(4): 403-411.	Title
177	de la Peña, A., et al. (2004). "PK-PD modelling of the effect of cefaclor on four different bacterial strains." Int J Antimicrob Agents 23(3): 218-225.	Title

178	De Maine, J. B. and W. M. Kirby (1970). "Clinical pharmacology of cephalexin administered intravenously." Antimicrob Agents Chemother (Bethesda) 10: 190-194.	Abstract
179	de Vrueh, R. L., et al. (1998). "Transport of L-valine-acyclovir via the oligopeptide transporter in the human intestinal cell line, Caco-2." J Pharmacol Exp Ther 286(3): 1166-1170.	Title
180	Dean, R. A., et al. (1994). "Simultaneous determination of primaquine and carboxyprimaquine in plasma using high-performance liquid chromatography with electrochemical detection." Journal of Chromatography B: Biomedical Sciences and Applications 655(1): 89-96.	Title
181	Dean, S., et al. (1979). "Absorption and excretion of cephalexin in health and acute illness." Eur J Clin Pharmacol 16(1): 73-74.	Abstract
182	Dellamonica, P., et al. (1993). "Pharmacokinetic and bacteriological study of cefadroxil-salicylate and josamycin-salicylate drug regimens." Int J Clin Pharmacol Res 13(1): 11-20.	Title
183	Deppermann, K. M., et al. (1990). "Comparative pharmacokinetics of cefotiam hexetil (CEHX), cefuroxime axetil (CEFUR), Cefixime (CEFIX), Cephalexine (CEPHA), and the influence of a standard breakfast, ranitidine, and Maalox 70 on enteral absorption of cefotiam hexetil." Klinische Wochenschrift 68(Suppl 19): 48-49.	Abstract
184	Deppermann, K. M., et al. (1989). "Influence of ranitidine, pirenzepine, and aluminum magnesium hydroxide on the bioavailability of various antibiotics, including amoxicillin, cephalexin, doxycycline, and amoxicillin-clavulanic acid." Antimicrob Agents Chemother 33(11): 1901-1907.	Full text
185	Derendorf, H. and S. Schmidt (2019). "Rowland and Tozer's clinical pharmacokinetics and pharmacodynamics: concepts and applications."	Title
186	Derrick, C. W., Jr. and K. Reilly (1983). "The role of cephalexin in the treatment of skin and soft-tissue infections." Postgrad Med J 59 Suppl 5: 43-46.	Abstract
187	Derry, J. E. (1981). "Evaluation of cefaclor." Am J Hosp Pharm 38(1): 54-58.	Title
188	Dey, N., et al. (2022). "Role of nanomaterials in deactivating multiple drug resistance efflux pumps – A review." Environmental Research 204: 111968.	Title
189	Dhopeshwarkar, V., et al. (1994). "Development of an oral sustained-release antibiotic matrix tablet using in-vitro/in-vivo correlations." Drug Dev Ind Pharm 20(11): 1851-1867.	Title
190	Dhuria, S. V., et al. (2009). "Intranasal drug targeting of hypocretin-1 (orexin-A) to the central nervous system." J Pharm Sci 98(7): 2501-2515.	Title

191	DiMattia, A. F., et al. (1981). "Efficacy of two dosage schedules of cephalexin in dermatologic infections." J Fam Pract 12(4): 649-652.	Abstract
192	Ding, H., et al. (2020). "Pharmacokinetic behavior of peramivir in the plasma and lungs of rats after trans-nasal aerosol inhalation and intravenous injection." 129: 110464.	Animal
193	Ding, Y., et al. (2013). "Effects of amlodipine on the oral bioavailability of cephalexin and cefuroxime axetil in healthy volunteers." J Clin Pharmacol 53(1): 82-86.	Abstract
194	DiPiro, J. T., et al. (1983). "The prophylactic use of antimicrobials in surgery." Current Problems in Surgery 20(2): 69-132.	Title
195	Dorow, P. (1989). "Safety and efficacy of cefixime versus cefaclor in respiratory tract infections." J Chemother 1(4): 257-260.	Title
196	Douglas, L. R., et al. (1998). "Oral management of the patient with end-stage liver disease and the liver transplant patient." Oral Surgery, Oral Medicine, Oral Pathology, Oral Radiology, and Endodontology 86(1): 55-64.	Title
197	Dubbelboer, I. R., et al. (2019). "Rat intestinal drug permeability: A status report and summary of repeated determinations." European Journal of Pharmaceutics and Biopharmaceutics 142: 364-376.	Animal
198	Dubinsky, S., et al. (2022). "Determining the Effects of Chronic Kidney Disease on Organic Anion Transporter1/3 Activity Through Physiologically Based Pharmacokinetic Modeling." 61(7): 997-1012.	Title
199	Dudley, M. N., et al. (1990). "Significance of "extravascular" protein binding for antimicrobial pharmacodynamics in an in vitro capillary model of infection." Antimicrob Agents Chemother 34(1): 98-101.	Title
200	Dvorácek, K., et al. (1976). "[A contribution on the pharmacokinetics of oral cephalosporin antibiotics (author's transl)]." Cas Lek Cesk 115(20-21): 622-627.	Abstract
201	Eichenwald, H. F. and H. J. Schmitt (1986). "The cephalosporin antibiotics in pediatric therapy." Eur J Pediatr 144(6): 532-538.	Title
202	Elder, M. G., et al. (1977). "Pelvic tissue and serum concentrations of various antibiotics given as pre-operative medication." Br J Obstet Gynaecol 84(12): 887-893.	Title
203	Ellison, M. J. and D. W. Crabtree (1990). "Antibiotic Therapy for Common Infections." Primary Care: Clinics in Office Practice 17(3): 521-541.	Title
204	Embil, J. M. and L. E. Nicolle (1999). "Antimicrobial Agents." Physical Medicine and Rehabilitation Clinics of North America 10(2): 403-436.	Title
205	Epling, D. (2019). Characterization of the Intestinal Permeability and Oral Absorption of Valacyclovir in Wildtype and huPepT1 Transgenic Mice.	Animal

206	Epling, D., et al. (2018). "Evaluating the intestinal and oral absorption of the prodrug valacyclovir in wildtype and huPepT1 transgenic mice." Biochem Pharmacol 155: 1-7.	Animal
207	Epstein, M. E., et al. (1997). "Antimicrobial agents for the dermatologist. I. β-Lactam antibiotics and related compounds." Journal of the American Academy of Dermatology 37(2, Part 1): 149-165.	Title
208	Fagerholm, U., et al. (2023). "Prediction of the Human Pharmacokinetics of 30 Modern Antibiotics Using the ANDROMEDA Software." bioRxiv: 2023.2003. 2028.534601.	Title
209	Fazulbhoy, R., et al. (2021). "A Comparative Analysis of Amoxicillin and Cefuroxime." International Journal of Scientific Research in Science and Technology 8: 96-124.	Title
210	Federspil, P., et al. (1979). "[Experience with cefaclor in the treatment of ear, nose and throat infections. Indications for cefaclor therapy (author's transl)]." Infection 7 Suppl 6: 609-614.	Title
211	Ferreira, C., et al. (2018). "The scaffold protein PDZK1 modulates expression and function of the organic anion transporting polypeptide 2B1." European Journal of Pharmaceutical Sciences 120: 181-190.	Title
212	Ferreira, J. M., et al. (2013). "Use of glass ionomer cement containing antibiotics to seal off infected dentin: a randomized clinical trial." Brazilian dental journal 24(1): 68-73.	Title
213	Fillastre, J. P., et al. (1980). "Cefaclor pharmacokinetics and renal impairment." J Antimicrob Chemother 6(1): 155-156.	Title
214	Finkelstein, E. R., et al. (1978). "Pharmacokinetics of oral cephalosporins." J Pediatr 93(5): 902.	Abstract
215	Flaherty, J. H. (1998). "Commonly Prescribed and Over-The-Counter Medications: Causes of Confusion." Clinics in Geriatric Medicine 14(1): 101-128.	Title
216	Flores-Murrieta, F. J., et al. (2001). "Oral pharmacokinetics of cefaclor in Mexican subjects." Proc West Pharmacol Soc 44: 71-72.	Title
217	Fong, I. W., et al. (1995). "Clarithromycin versus cefaclor in lower respiratory tract infections. The Canadian Bronchitis Study Group." Clinical and investigative medicine. Medecine clinique et experimentale 18(2): 131-138.	Title
218	Force, T. M. A. and T. M. A. R. Team. "Keppra suspension dosing hydrocodone."	Title
219	Foster, D. R., et al. (2009). "Lack of interaction between the peptidomimetic substrates captopril and cephradine." J Clin Pharmacol 49(3): 360-367.	Title
220	Foster, D. R. and X. Zheng (2007). "Cephalexin inhibits N-formylated peptide transport and intestinal hyperpermeability in Caco2 cells." J Pharm Pharm Sci 10(3): 299-310.	Abstract

221	Fowler, S., et al. (2022). "Addressing today’s Absorption, Distribution, Metabolism, and Excretion (ADME) challenges in the translation of in vitro ADME characteristics to humans: a case study of the SMN2 mRNA splicing modifier risdiplam." Drug metabolism disposition 50(1): 65-75.	Title
222	Fowler, S., et al. (2021). "Addressing today's ADME challenges in the translation of in vitro absorption, distribution, metabolism and excretion characteristics to human: A case study of the SMN2 mRNA splicing modifier risdiplam." Drug metabolism disposition.	Title
223	Franke, A. A., et al. (2012). "Equol production changes over time in postmenopausal women." The Journal of Nutritional Biochemistry 23(6): 573-579.	Title
224	Fraschini, F., et al. (1988). "Correlation between plasma and tonsillar levels of cefroxadine." Chemotherapy 34(1): 8-12.	Title
225	Freeman, C. D., et al. (1994). "Minocycline: old and new therapeutic uses." Int J Antimicrob Agents 4(4): 325-335.	Title
226	Fresno, L., et al. (2008). "Placental transfer of etomidate in pregnant ewes after an intravenous bolus dose and continuous infusion." The Veterinary Journal 175(3): 395-402.	Title
227	Fujimori, I. (1982). "[Cefadroxil]." Jpn J Antibiot 35(12): 2736-2747.	Title
228	Fujimoto, M., et al. (1985). "[Pharmacological and clinical studies on the long acting cefaclor (S6472)]." Jpn J Antibiot 38(3): 849-857.	Title
229	Fujita, K., et al. (1983). "Clinical Evaluation of Norfloxacin in the Treatment of Superficial Suppurative Skin and Soft Tissue Infections -A Double-blind Study in Comparison with L-Cephalexin." Rinsho hyoka (clinical evaluation) 11(1): 133-154.	Title
230	Fujita, K., et al. (1982). "Clinical Evaluation of Cefadroxil in the Treatment of Superficial Suppurative Skin and Soft Tissue Infections - A Double-blind Study Comparing to L-Cephalexin." Rinsho hyoka (clinical evaluation) 10(1): 175-200.	Title
231	Ganapathy, M. E., et al. (2000). "beta-lactam antibiotics as substrates for OCTN2, an organic cation/carnitine transporter." J Biol Chem 275(3): 1699-1707.	Title
232	Gandhi, M. D. (2005). An amino acid transporter targeted approach for the treatment of cytomegalovirus infections, University of Missouri-Kansas City.	Title
233	Gao, S., et al. (2008). "Determination of faropenem in human plasma and urine by liquid chromatography-tandem mass spectrometry." Biomed Chromatogr 22(1): 5-12.	Title
234	García-Rodríguez, J. A., et al. (1995). "Oral cephalosporins: current perspectives." Int J Antimicrob Agents 5(4): 231-243.	Abstract
235	Garrigues, T. M., et al. (1991). "Dose-dependent absorption and elimination of cefadroxil in man." Eur J Clin Pharmacol 41(2): 179-183.	Title

236	Garrison, D. A. (2022). Interaction of Gilteritinib, a novel FLT-3 Tyrosine Kinase Inhibitor, with Xenobiotic Uptake Transporters, The Ohio State University.	Title
237	Garrison, K. L., et al. (2015). "Few Drugs Display Flip-Flop Pharmacokinetics and These Are Primarily Associated with Classes 3 and 4 of the BDDCS." J Pharm Sci 104(9): 3229-3235.	Title
238	Gartenberg, G., et al. (1979). "Pharmacokinetics of cefaclor in patients with stable renal impairment, and patients undergoing haemodialysis." J Antimicrob Chemother 5(4): 465-470.	Title
239	Genvresse, I. and C. Carbon (1993). "Cefixime." Int J Antimicrob Agents 3(1): 1-16.	Title
240	George, M. (2013). The role of organic cation transporters in the nasal uptake and brain distribution of organic cation substrates, The University of Iowa.	Title
241	Gerardin, A., et al. (1982). "Pharmacokinetics of cefroxadin (CGP 9000) in man." J Pharmacokinet Biopharm 10(1): 15-26.	Title
242	Gerber, M. A., et al. (1999). "Potential mechanisms for failure to eradicate group A streptococci from the pharynx." Pediatrics 104(4 Pt 1): 911-917.	Title
243	Ghafelehbashi, R., et al. (2019). "Preparation, physicochemical properties, in vitro evaluation and release behavior of cephalexin-loaded niosomes." Int J Pharm 569: 118580.	Abstract
244	Ghafourian, T., et al. (2006). "QSPR models for the prediction of apparent volume of distribution." Int J Pharm 319(1): 82-97.	Title
245	Gibson, M. J., et al. (1987). "The penetration of antibiotics into the normal intervertebral disc." J Bone Joint Surg Br 69(5): 784-786.	Title
246	Gillett, A. P. and Wise (1978). "Penetration of four cephalosporins into tissue fluid in man." Lancet 1(8071): 962-964.	Title
247	Ginsburg, C. M. and G. H. McCracken, Jr. (1979). "Pharmacokinetics of cephradine suspension infants and children." Antimicrob Agents Chemother 16(1): 74-76.	Title
248	Ginsburg, C. M. and G. H. McCracken, Jr. (1980). "Bioavailability of cefadroxil capsules and suspension in pediatric patients." J Int Med Res 8(Suppl 1): 9-14.	Title
249	Gisby, J., et al. (1991). "Comparative efficacies of ciprofloxacin, amoxicillin, amoxicillin-clavulanic acid, and cefaclor against experimental Streptococcus pneumoniae respiratory infections in mice." Antimicrob Agents Chemother 35(5): 831-836.	Animal
250	Gleckman, R., et al. (1981). "Trimethoprim: mechanisms of action, antimicrobial activity, bacterial resistance, pharmacokinetics, adverse reactions, and therapeutic indications." Pharmacotherapy 1(1): 14-20.	Title

251	Goldstein, F. W. (1997). "Choice of an oral beta-lactam antibiotic for infections due to penicillin-resistant Streptococcus pneumoniae." Scand J Infect Dis 29(3): 255-257.	Title
252	Gonçalves, T. M., et al. (2008). "Determination of ampicillin in human plasma by solid-phase extraction–liquid chromatography–tandem mass spectrometry (spe-lc-ms/ms) and its use in bioequivalence studies." Arzneimittelforschung 58(02): 91-96.	Title
253	Gong, Q., et al. (2007). "Pharmacokinetic interaction between cefaclor and bromhexine in healthy Chinese volunteers." Zhong Nan Da Xue Xue Bao Yi Xue Ban 32(5): 855-861.	Title
254	Gonzalez, I. (1989). Absorption of flurbiprofen and some other drugs through the oral mucosa, University of Michigan.	Title
255	Goumas, P. D., et al. (1992). "Pharmacokinetic study of cefaclor in chronic maxillary sinusitis." J Chemother 4(3): 155-158.	Title
256	Grassi, G. G. (1994). "Comparative clinical efficacy of cefetamet pivoxil in lower respiratory tract infection." Drugs 47 Suppl 3: 35-42.	Title
257	Green, E. R., et al. (1981). "A Comparison of the Penetration Characteristics of Cephapirin and Cephalothin into Right Atrial Appendage, Muscle, Fat, and Pericardial Fluid of Pediatric Patients Undergoing Open-Heart Operation." The Annals of Thoracic Surgery 31(2): 155-160.	Title
258	Greene, D. S., et al. (1976). "Pharmacokinetics of cephalexin: an evaluation of one- and two-compartment model pharmacokinetics." J Clin Pharmacol 16(5-6): 257-264.	Full text
259	Greenfield, R. A. (1993). "Symposium on antimicrobial therapy. IV. The cephalosporins." J Okla State Med Assoc 86(1): 7-12.	Title
260	Griffith, R. S. (1983). "The pharmacology of cephalexin." Postgrad Med J 59 Suppl 5: 16-27.	Title
261	Griffith, R. S. and H. R. Black (1970). "Cephalexin." Med Clin North Am 54(5): 1229-1244.	Abstract
262	Groneberg, D. A., et al. (2001). "Intestinal peptide transport: ex vivo uptake studies and localization of peptide carrier PEPT1." Am J Physiol Gastrointest Liver Physiol 281(3): G697-704.	Title
263	Groneberg, D. A., et al. (2002). "Distribution and function of the peptide transporter PEPT2 in normal and cystic fibrosis human lung." Thorax 57(1): 55-60.	Title
264	Grujić, Z., et al. (2009). "[Single dose of antibiotic prophylaxis in elective cesarean sections]." Med Pregl 62(3-4): 101-106.	Title
265	Guay, D. R. (2004). "Oritavancin and tigecycline: investigational antimicrobials for multidrug-resistant bacteria." Pharmacotherapy: The Journal of Human Pharmacology Drug Therapy 24(1): 58-68.	Title
266	Guay, D. R. P. (2002). "Cefdinir: An advanced-generation, broad-spectrum oral cephalosporin." Clin Ther 24(4): 473-489.	Title

267	Gubbins, P. O., et al. (1998). "Rapid and sensitive high performance liquid chromatographic method for the determination of itraconazole and its hydroxy-metabolite in human serum1Presented at the Analysis and Pharmaceutical Quality Section of the Eleventh Annual American Association of Pharmaceutical Scientists Meeting, October 1996, Seattle, Washington, USA.1." J Pharm Biomed Anal 16(6): 1005-1012.	Title
268	Guggenbichler, J. P. and G. Kienel (1978). "[Bioavailability of new galenic preparations of cephalexin (author's transl)]." Padiatr Padol 13(3): 315-320.	Title
269	Guggenbichler, J. P. and G. Kienel (1979). "Bioavailability of oral antibiotics in cystic fibrosis." Monogr Paediatr 10: 34-40.	Title
270	Guggenbichler, J. P. and G. Kienel (1979). "[Bioavailability of orally administered antibiotics: influences of food on resorption (author's transl)]." Padiatr Padol 14(1): 69-74.	Title
271	Guggenbichler, J. P., et al. (1981). "[Pharmacokinetic of antibiotics in patients with mucoviscidosis (author's transl)]." Padiatr Padol 16(4): 393-402.	Title
272	Guggenbichler, J. P. and J. Schneeberger (1987). "Antimicrobial chemotherapy in patients with cystic fibrosis." Infection 15(5): 397-402.	Title
273	Gupta, A., et al. (2013). "Simultaneous quantification of prodrug oseltamivir and its metabolite oseltamivir carboxylate in human plasma by LC–MS/MS to support a bioequivalence study." Journal of Pharmaceutical Analysis 3(3): 149-160.	Title
274	Gwee, A., et al. (2020). "Twice- and Thrice-daily Cephalexin Dosing for Staphylococcus aureus Infections in Children." Pediatr Infect Dis J 39(6): 519-522.	Abstract
275	Hagihara, M., et al. (2021). "Pharmacodynamic evaluation of meropenem, cefepime, or aztreonam combined with a novel β-lactamase inhibitor, nacubactam, against carbapenem-resistant and/or carbapenemase-producing Klebsiella pneumoniae and Escherichia coli using a murine thigh-infection model." Int J Antimicrob Agents 57(5): 106330.	Animal
276	Haginaka, J., et al. (1980). "Chromatographic analysis and pharmacokinetic investigation of cephaloglycin and its metabolites in man." J Antibiot (Tokyo) 33(2): 236-243.	Title
277	Haginaka, J., et al. (1979). "Evaluation of effect of food ingestion on bioavailability of cephalexin by moment analysis." Chem Pharm Bull (Tokyo) 27(12): 3156-3159.	Abstract
278	Haines, S. T. and M. A. Kraynak (1994). "Twice-daily cefaclor therapy." Ann Pharmacother 28(12): 1353-1355.	Title
279	Hammami, M. M., et al. (2017). "Generic-reference and generic-generic bioequivalence of forty-two, randomly-selected, on-market generic products of fourteen immediate-release oral drugs." BMC Pharmacol Toxicol 18(1): 78.	Title

280	Hammami, M. M., et al. (2017). "Does the placebo effect modulate drug bioavailability? Randomized cross-over studies of three drugs." J Negat Results Biomed 16(1): 10.	Title
281	Hammond, D. and M. A. Abate (1989). "Theophylline toxicity, acute illness, and cefaclor administration." Dicp 23(4): 339-340.	Title
282	Hampel, B., et al. (1982). "Pharmacokinetics of cefadroxil and cefaclor during an eight-day dosage period." Antimicrob Agents Chemother 22(6): 1061-1063.	Title
283	Hampson, N. B., et al. (1991). "Oral Antibiotics for Pneumonia." Clinics in Chest Medicine 12(2): 395-407.	Title
284	Han, H. K., et al. (1998). "Cellular uptake mechanism of amino acid ester prodrugs in Caco-2/hPEPT1 cells overexpressing a human peptide transporter." Pharm Res 15(9): 1382-1386.	Title
285	Han, S. Y., et al. (2012). "Reduced clearance of ε-acetamidocaproic acid in rats with acute renal failure induced by uranyl nitrate." Journal of Pharmacy Pharmacology 64(10): 1452-1460.	Animal
286	Hansen, E., et al. (2014). "Perioperative Antibiotics." The Journal of Arthroplasty 29(2, Supplement): 29-48.	Title
287	Hara, J., et al. (1985). "Study on the transfer of cefroxadine to human tears." Japanese journal of antibiotics 38(2): 230-234.	Title
288	Harrison, C. J., et al. (1985). "A multicenter comparison of related pharmacologic features of cephalexin and dicloxacillin given for two months to young children with cystic fibrosis." Pediatr Pharmacol (New York) 5(1): 7-16.	Abstract
289	Hartstein, A. I., et al. (1977). "Comparison of pharmacological and antimicrobial properties of cefadroxil and cephalexin." Antimicrob Agents Chemother 12(1): 93-97.	Abstract
290	Harvengt, C., et al. (1973). "Cephradine absorption and excretion in fasting and nonfasting volunteers." J Clin Pharmacol New Drugs 13(1): 36-40.	Title
291	Hayakawa, F., et al. (1989). "[Laboratory and clinical studies on cefteram pivoxil in the field of pediatrics]." Jpn J Antibiot 42(8): 1849-1859.	Title
292	Henry, N. K., et al. (2000). "Antimicrobial Therapy for Infants and Children: Guidelines for the Inpatient and Outpatient Practice of Pediatric Infectious Diseases." Mayo Clinic Proceedings 75(1): 86-97.	Title
293	Henwood, J. M. and J. P. Monk (1988). "Enoxacin. A review of its antibacterial activity, pharmacokinetic properties and therapeutic use." Drugs 36(1): 32-66.	Title
294	Hermann, R., et al. (1996). "Enantioselective pharmacokinetics and bioavailability of different racemic α-lipoic acid formulations in healthy volunteers." European Journal of Pharmaceutical Sciences 4(3): 167-174.	Title

295	Hernandez, B. N., et al. (2023). "MIC Discrepancies between Parenteral and Oral Anti-Staphylococcal Beta-Lactams among MSSA." Chemotherapy 68(1): 55-60.	Title
296	Hernández, M., et al. (2003). "Analysis of antibiotics in biological samples by capillary electrophoresis." TrAC Trends in Analytical Chemistry 22(7): 416-427.	Title
297	Herrera-Ruiz, D. and G. T. Knipp (2003). "Current Perspectives on Established and Putative Mammalian Oligopeptide Transporters." J Pharm Sci 92(4): 691-714.	Animal
298	Hicks, M. J. and C. M. Flaitz (1993). "The role of antibiotics in platelet dysfunction and coagulopathy." Int J Antimicrob Agents 2(3): 129-149.	Title
299	Hidalgo, I. J., et al. (1993). "pH-dependent transepithelial transport of cephalexin in rabbit intestinal mucosa." Int J Pharm 98(1): 83-92.	Animal
300	Hirata-Dulas, C. A. I., et al. (1994). "Evaluation of Two Intravenous Single-Bolus Methods for Measuring Effective Renal Plasma Flow." American Journal of Kidney Diseases 23(3): 374-381.	Title
301	Hirayama, F. and K. Uekama (2007). "Colonic delivery." Biotechnology: Pharmaceutical Aspects Ronald T. Borchardt	Title
302	C. Russell Middaugh, Series Editors Volume I: Pharmaceutical Profiling in Drug Discovery for Lead Selection.	Title
303	Hiremath, C. N. (2021). "Abbreviated Profile of Drugs (APOD): modeling drug safety profiles to prioritize investigational COVID-19 treatments." Heliyon 7(8): e07666.	Title
304	Hochman, J., et al. (2015). "Drug–drug interactions related to altered absorption and plasma protein binding: Theoretical and regulatory considerations, and an industry perspective." J Pharm Sci 104(3): 916-929.	Title
305	Hoekman, J. D. (2010). The Impact of Enhanced Olfactory Deposition and Retention on Direct Nose-to-Brain Drug Delivery, University of Washington.	Title
306	Hoepelman, I. M. and M. M. E. Schneider (1995). "Azithromycin: the first of the tissue-selective azalides." Int J Antimicrob Agents 5(3): 145-167.	Title
307	Höffler, D. and P. Koeppe (1975). "[Pharmacokinetics of intravenous Cefradin in normal and restricted renal function (author's transl)]." MMW Munch Med Wochenschr 117(27): 1169-1174.	Title
308	Holm, S. E. (1994). "Reasons for failures in penicillin treatment of streptococcal tonsillitis and possible alternatives." Pediatr Infect Dis J 13(1 Suppl 1): S66-69; discussion S78-69.	Title
309	Hopkins, S. (1991). "Clinical toleration and safety of azithromycin." The American Journal of Medicine 91(3, Supplement 1): S40-S45.	Title

310	Hori, R., et al. (1983). "Ampicillin and cephalexin in renal insufficiency." Clin Pharmacol Ther 34(6): 792-798.	Abstract
311	Hori, R., et al. (1985). "A new dosing regimen in renal insufficiency: application to cephalexin." Clin Pharmacol Ther 38(3): 290-295.	Abstract
312	Horii, M., et al. (1985). "Clinical evaluation of sustained release preparations of cefaclor in dental infections. Comparative double blind clinical studies of sustained release preparations with a regular preparation of cefaclor." Japanese journal of antibiotics 38(4): 1117-1140.	Title
313	Howard, C. R. and R. A. Lawrence (1999). "Drugs and Breastfeeding." Clinics in Perinatology 26(2): 447-478.	Title
314	Hu, L., et al. (2011). "Floating matrix dosage form for dextromethorphan hydrobromide based on gas forming technique: in vitro and in vivo evaluation in healthy volunteers." 42(1-2): 99-105.	Title
315	Hu, M., et al. (1996). "Uptake characteristics of loracarbef and cephalexin in the Caco-2 cell culture model: effects of the proton gradient and possible presence of a distinctive second component." J Pharm Sci 85(7): 767-772.	Abstract
316	Hu, M., et al. (1994). "Mechanism and kinetics of transcellular transport of a new beta-lactam antibiotic loracarbef across an intestinal epithelial membrane model system (Caco-2)." Pharm Res 11(10): 1405-1413.	Abstract
317	Hu, M., et al. (1999). "Transport and metabolic characterization of Caco-2 cells expressing CYP3A4 and CYP3A4 plus oxidoreductase." Pharm Res 16(9): 1352-1359.	Title
318	Hu, Y. (2015). Development, characterization and application of a novel mouse line humanized for the intestinal peptide transporter PEPT1, University of Michigan.	Animal
319	Hu, Y. and D. E. Smith (2016). "Species differences in the pharmacokinetics of cefadroxil as determined in wildtype and humanized PepT1 mice." Biochem Pharmacol 107: 81-90.	Animal
320	Hu, Y. and D. E. Smith (2019). "In Silico Prediction of the Absorption and Disposition of Cefadroxil in Humans using an Intestinal Permeability Method Scaled from Humanized PepT1 Mice." Drug Metab Dispos 47(3): 173-183.	Animal
321	Huang, C., et al. (2014). "Determination of cefaclor by UPLC-MS-MS for a Chinese pharmacokinetic study." J Chromatogr Sci 52(7): 636-640.	Title
322	Hubsher, J. A., et al. (1976). "Double-blind comparison of cephradine and cephalexin in the treatment of skin and soft-tissue infections due to Staphylococcus aureus." Curr Ther Res Clin Exp 19(6): 579-588.	Abstract
323	Huh, Y. M. (2013). Effect of Inflammation on Cefadroxil Pharmacokinetics in Brain and Kidney and Pharmacometric Modeling of PEPT2-Mediated Disposition of Glycylsarcosine in Brain.	Title
324	Humair, L. (1973). "[Antibiotic therapy in renal failure (author's transl)]." Munch Med Wochenschr 115(39): 1657-1660.	Title

325	Huo, X., et al. (2014). "Inhibitory Effect of Valsartan on the Intestinal Absorption and Renal Excretion of Bestatin in Rats." J Pharm Sci 103(2): 719-729.	Animal
326	Hurst, D. J. (1984). "A comparison of cefaclor and tetracycline in the treatment of bacterial bronchitis." Clin Ther 6(2): 163-169.	Title
327	Iakovlev, V. P., et al. (1977). "[Pharmacokinetics of cephalexin and cephradine in surgical patients]." Antibiotiki 22(8): 739-742.	Abstract
328	Ilett, K. F., et al. (2006). "Transfer of probenecid and cephalexin into breast milk." Ann Pharmacother 40(5): 986-989.	Title
329	Imran, M., et al. (2016). "Glycoside-based niosomal nanocarrier for enhanced in-vivo performance of Cefixime." Int J Pharm 505(1): 122-132.	Title
330	Inoue, D., et al. (2020). "Effect of cerebrospinal fluid circulation on nose-to-brain direct delivery and distribution of caffeine in rats." Molecular Pharmaceutics 17(11): 4067-4076.	Animal
331	Inui, K., et al. (1992). "Transepithelial transport of oral cephalosporins by monolayers of intestinal epithelial cell line Caco-2: specific transport systems in apical and basolateral membranes." J Pharmacol Exp Ther 261(1): 195-201.	Title
332	Iravani, A. and W. Bischoff (1992). "Antibiotic therapy for urinary tract infections." The American Journal of Medicine 92(6, Supplement 1): S95-S100.	Title
333	Iseki, K., et al. (1994). "[Effect of Enterued administration on the intestinal absorption of orally active cefem antibiotics]." Yakugaku Zasshi 114(4): 233-240.	Title
334	Ishikawa, J., et al. (1988). "[Clinical studies on sultamicillin fine granule in pediatric field]." Jpn J Antibiot 41(12): 1965-1971.	Title
335	Isla, A., et al. (2005). "Análisis farmacocinético/farmacodinámico (PK/PD) de la antibioterapia en odontoestomatología." Enfermedades Infecciosas y Microbiología Clínica 23(3): 116-121.	Language
336	Ito, S., et al. (1993). "Prospective follow-up of adverse reactions in breast-fed infants exposed to maternal medication." American Journal of Obstetrics and Gynecology 168(5): 1393-1399.	Title
337	Ito, S., et al. (2012). "Competitive inhibition of the luminal efflux by multidrug and toxin extrusions, but not basolateral uptake by organic cation transporter 2, is the likely mechanism underlying the pharmacokinetic drug-drug interactions caused by cimetidine in the kidney." J Pharmacol Exp Ther 340(2): 393-403.	Title
338	Iwai, N., et al. (1986). "[Fundamental and clinical studies on cefixime in pediatrics]." Jpn J Antibiot 39(4): 1087-1105.	Title
339	Izquierdo-Barba, I., et al. (2016). "Zwitterionic ceramics for biomedical applications." Acta Biomaterialia 40: 201-211.	Title

340	Jackson, A. J. and M. L. Chen (1987). "Application of moment analysis in assessing rates of absorption for bioequivalency studies." J Pharm Sci 76(1): 6-9.	Title
341	Jacobs, M. R., et al. (2007). "Oral beta-lactams applied to uncomplicated infections of skin and skin structures." Diagn Microbiol Infect Dis 57(3 Suppl): 55s-65s.	Title
342	Jahanfar, S., et al. (2013). "Antibiotics for mastitis in breastfeeding women." Cochrane Database of Systematic Reviews(2).	Title
343	Jain, D. S., et al. (2016). "Thermosensitive PLA based nanodispersion for targeting brain tumor via intranasal route." Materials Science and Engineering: C 63: 411-421.	Title
344	James, A. J., et al. (2020). "Pharmacokinetic profile of gilteritinib: a novel FLT-3 tyrosine kinase inhibitor." Clin Pharmacokinet 59: 1273-1290.	Title
345	James, N. C., et al. (1991). "Pharmacokinetics of cefuroxime axetil and cefaclor: relationship of concentrations in serum to MICs for common respiratory pathogens." Antimicrob Agents Chemother 35(9): 1860-1863.	Title
346	Jana, S. and H. Rastogi (2017). "Effects of caffeic acid and quercetin on in vitro permeability, metabolism and in vivo pharmacokinetics of melatonin in rats: potential for herb-drug interaction." European journal of drug metabolism pharmacokinetics 42: 781-791.	Animal
347	Jappar, D. (2009). Role of proton-coupled oligopeptide transporters in small peptide absorption and disposition, University of Michigan.	Title
348	Jappar, D., et al. (2011). "Effect of dose escalation on the in vivo oral absorption and disposition of glycylsarcosine in wild-type and Pept1 knockout mice." Drug metabolism disposition 39(12): 2250-2257.	Animal
349	Jayasagar, G., et al. (2002). "Effect of cephalexin on the pharmacokinetics of metformin in healthy human volunteers." Drug Metabol Drug Interact 19(1): 41-48.	Full text
350	Jeong, S.-H., et al. (2021). "Population pharmacokinetic analysis of cefaclor in healthy Korean subjects." Pharmaceutics 13(5): 754.	Title
351	Johansson, S., et al. (2017). "Tenapanor administration and the activity of the H(+) -coupled transporter PepT1 in healthy volunteers." Br J Clin Pharmacol 83(9): 2008-2014.	Title
352	Johnson, C. A., et al. (1984). "Pharmacokinetics of oral cephradine in continuous ambulatory peritoneal dialysis patients." Nephron 38(1): 57-61.	Title
353	Johnson, D. M., et al. (2000). "Antimicrobial activity and in vitro susceptibility test development for cefditoren against Haemophilus influenzae, Moraxella catarrhalis, and Streptococcus species." Diagn Microbiol Infect Dis 37(2): 99-105.	Title

354	Johnson, V. M., et al. (2000). "Determination of the cephalosporin antibiotic cephradine in human plasma by high-performance liquid chromatography with ultraviolet detection." J Chromatogr B Biomed Sci Appl 740(1): 71-80.	Title
355	Jones, S., et al. (1985). "Pharmacokinetic and therapeutic trial of sultamicillin in acute sinusitis." Antimicrob Agents Chemother 28(6): 832-833.	Title
356	Jonkman, J. H. and R. A. Upton (1984). "Pharmacokinetic drug interactions with theophylline." Clin Pharmacokinet 9(4): 309-334.	Title
357	Since up to 90% of a theophylline dose is biotransformed, drugs influencing microsomal enzyme systems in the liver may affect the elimination of theophylline. Other integrated mechanisms (e.g., hepatic uptake) may also be altered by concurrent administration of other drugs. Whatever the mechanism, the interaction may be sufficient to necessitate adjustment of the theophylline dosage, preferably guided by plasma theophylline determinations. Comedication with phenobarbitone may require an increase in theophylline dose by about 30% due to increased clearance resulting from enzyme induction. Similarly, with phenytoin and carbamazepine, a dose increase of about 40-50% may be required. In the case of rifampicin, isoniazid, or sulphinpyrazone comedication, an increase in dose of theophylline by about 20-25% may be needed. On the other hand, other drugs decrease theophylline clearance, making a reduction in the dose of concurrent theophylline advisable; with usual doses of erythromycin, propranolol, and isoprenaline (isoproterenol), a reduction of about 25% is needed; with cimetidine and oral contraceptive by about 30% or more; and with triacetyloleandomycin (troleandomycin), by about 50%. In high doses, the xanthine oxidase inhibitor allopurinol can also retard theophylline elimination, and a reduction of the theophylline dose by about 20% may be advisable. Conflicting results have been reported on the influence of frusemide (furosemide) and influenza vaccines, while data regarding the effect of corticosteroids, benzodiazepines, and verapamil on theophylline kinetics are not yet conclusive. Many drugs, however, appear not to significantly affect theophylline clearance. Some are from the same therapeutic group as the drugs mentioned above and offer clinical alternatives for coadministration with theophylline. Examples of drugs not found to have a significant effect on theophylline pharmacokinetics are ranitidine, josamycin, midecamycin, amoxycillin, tetracycline, cephalexin, cefaclor, orciprenaline, metoprolol, antacids, medroxyprogesterone acetete, metoclopramide, and metronidazole. Most of the drugs discussed in this review appear to not affect the volume of distribution of theophylline significantly.	Title
358	Jonkman, J. H., et al. (1985). "No effect of cefaclor on theophylline pharmacokinetics." Eur J Respir Dis 66(1): 47-49.	Title
359	Jonkman, J. H., et al. (1986). "Clinical pharmacokinetics of theophylline during co-treatment with cefaclor." Int J Clin Pharmacol Ther Toxicol 24(2): 88-92.	Title
360	Jung, H., et al. (1991). "Bioavailability of cephalexine dosage forms." Drug Dev Ind Pharm 17(16): 2173-2183.	Abstract

361	Kafetzis, D. A. (1994). "Multi-investigator evaluation of the efficacy and safety of cefprozil, amoxicillin-clavulanate, cefixime and cefaclor in the treatment of acute otitis media." European journal of clinical microbiology & infectious diseases 13(10): 857-865.	Title
362	Kamal, M. A., et al. (2008). "Role and relevance of PEPT2 in drug disposition, dynamics, and toxicity." Drug Metab Pharmacokinet 23(4): 236-242.	Title
363	Kamath, A. V., et al. (2005). "Multiple pathways are involved in the oral absorption of BMS-262084, a tryptase inhibitor, in rats: role of paracellular transport, binding to trypsin, and P-glycoprotein efflux." J Pharm Sci 94(5): 1115-1123.	Animal
364	Kamiya, A., et al. (1983). "Quantitative investigation on renal handling of drugs in rabbits, dogs, and humans." J Pharm Sci 72(4): 440-443.	Animal
365	Kammer, R. B. and R. Ress (1991). "Randomized comparative study of ceftibuten versus cefaclor in the treatment of acute lower respiratory tract infections." Diagn Microbiol Infect Dis 14(1): 101-105.	Title
366	Kang, Y.-S., et al. (2006). "A specific and rapid HPLC assay for the determination of cefroxadine in human plasma and its application to pharmacokinetic study in Korean." J Pharm Biomed Anal 40(2): 369-374.	Title
367	Kano, E. K., et al. (2008). "Bioequivalence study of two oral formulations of cefadroxil in healthy volunteers." Arzneimittelforschung 58(1): 42-47.	Title
368	Kaplan, N., et al. (2013). "AFN-1252 in vitro absorption studies and pharmacokinetics following microdosing in healthy subjects." European Journal of Pharmaceutical Sciences 50(3): 440-446.	Title
369	Karaoui, L. R., et al. (2013). "Oritavancin: an investigational lipoglycopeptide antibiotic." Am J Health Syst Pharm 70(1): 23-33.	Title
370	Karbownik, A., et al. (2020). "Pharmacokinetic interaction between sorafenib and atorvastatin, and sorafenib and metformin in rats." Pharmaceutics 12(7): 600.	Animal
371	Karim, S., et al. (2003). "The effect of four different types of food on the bioavailability of cefaclor." Eur J Drug Metab Pharmacokinet 28(3): 185-190.	Title
372	Kato, K., et al. (2014). "Investigation of endogenous compounds for assessing the drug interactions in the urinary excretion involving multidrug and toxin extrusion proteins." Pharm Res 31: 136-147.	Title
373	Kato, Y., et al. (2009). "Investigation of the role of oligopeptide transporter PEPT1 and sodium/glucose cotransporter SGLT1 in intestinal absorption of their substrates using small GTP-binding protein Rab8-null mice." Drug metabolism disposition 37(3): 602-607.	Animal

374	Katragadda, S., et al. (2008). "Pharmacokinetics of amino acid ester prodrugs of acyclovir after oral administration: interaction with the transporters on Caco-2 cells." Int J Pharm 362(1-2): 93-101.	Title
375	Kawami, M., et al. (2017). "Effect of Curcuma comosa extracts on the functions of peptide transporter and P-glycoprotein in intestinal epithelial cells." Pharmazie 72(2): 123-127.	Title
376	Kearns, G. L., et al. (1994). "Serum sickness-like reactions to cefaclor: role of hepatic metabolism and individual susceptibility." J Pediatr 125(5 Pt 1): 805-811.	Title
377	Kerin, M. J., et al. (1992). "Is antibiotic penetration compromised in the ischaemic tissues of patients undergoing amputation?" Ann R Coll Surg Engl 74(4): 274-276.	Title
378	Kesarwani, K. and R. Gupta (2013). "Bioavailability enhancers of herbal origin: An overview." Asian Pacific Journal of Tropical Biomedicine 3(4): 253-266.	Title
379	Khamdang, S., et al. (2003). "Interaction of human and rat organic anion transporter 2 with various cephalosporin antibiotics." Eur J Pharmacol 465(1-2): 1-7.	Animal
380	Khan, B. A., et al. (2004). "Comparative effect of different types of food on the bioavailability of cefaclor extended release tablet." Eur J Drug Metab Pharmacokinet 29(2): 125-132.	Title
381	Kiani, R. (1991). "Double-blind, double-dummy comparison of azithromycin and cephalexin in the treatment of skin and skin structure infections." European journal of clinical microbiology & infectious diseases 10(10): 880-884.	Abstract
382	Kim, Y. C., et al. (2014). "Effects of 1α, 25-dihydroxyvitamin D3, the natural vitamin D receptor ligand, on the pharmacokinetics of cefdinir and cefadroxil, organic anion transporter substrates, in rat." J Pharm Sci 103(11): 3793-3805.	Animal
383	Kimoto, E., et al. (2017). "Hepatobiliary Clearance Prediction: Species Scaling From Monkey, Dog, and Rat, and In Vitro–In Vivo Extrapolation of Sandwich-Cultured Human Hepatocytes Using 17 Drugs." J Pharm Sci 106(9): 2795-2804.	Animal
384	Kimura, H., et al. (1989). "[Bacteriological, pharmacokinetic and clinical studies on cefpodoxime proxetil in the pediatric field]." Jpn J Antibiot 42(7): 1593-1606.	Title
385	Kinasewitz, G. and R. G. Wood (1991). "Azithromycin versus cefaclor in the treatment of acute bacterial pneumonia." European journal of clinical microbiology & infectious diseases 10(10): 872-877.	Title
386	Kirby, W. M. and C. Regamey (1973). "Pharmacokinetics of cefazolin compared with four other cephalosporins." J Infect Dis 128: Suppl:S341-346.	Title

387	Kiss, I. J., et al. (1976). "Serum and lung tissue levels of cephradine in thoracic surgery." Br J Clin Pharmacol 3(5): 891-895.	Title
388	Kitaura, T., et al. (1989). "Ocular pharmacokinetics of latamoxef and cefaclor in humans. Penetration into aqueous humor." J Pharmacobiodyn 12(1): 60-66.	Title
389	Klimova, V. S. and V. P. Iakovlev (1979). "[Comparative study of the interaction of cephalosporins with blood serum proteins and organ homogenates]." Antibiotiki 24(11): 835-838.	Title
390	Klixbüll, U. and H. Bundgaard (1984). "Prodrugs as drug delivery systems. XXX. 4-Imidazolidinones as potential bioreversible derivatives for the α-aminoamide moiety in peptides." Int J Pharm 20(3): 273-284.	Title
391	Kobayashi, H., et al. (1986). "Comparative clinical study of T-2588 and cefaclor in chronic respiratory tract infections by a double-blind method." Kansenshogaku Zasshi 60(9): 1052-1077.	Title
392	Kodaira, H., et al. (2011). "Quantitative evaluation of the impact of active efflux by p-glycoprotein and breast cancer resistance protein at the blood-brain barrier on the predictability of the unbound concentrations of drugs in the brain using cerebrospinal fluid concentration as a surrogate." Journal of Pharmacology Experimental Therapeutics 339(3): 935-944.	Title
393	Komarova, V. P., et al. (1997). "[Treatment of lower respiratory tract infections in the elderly]." Antibiot Khimioter 42(12): 19-24.	Title
394	Koot, M. J., et al. (1992). "High pressure liquid chromatographic analysis of the serum concentration of cefuroxime after an intravenous bolus injection of cefuroxime in patients with a coronary artery bypass grafting." Pharm Weekbl Sci 14(6): 360-364.	Title
395	Korzeniowski, O. M., et al. (1977). "Comparative pharmacology of cefaclor and cephalexin." Antimicrob Agents Chemother 12(2): 157-162.	Title
396	Kovach, P. M., et al. (1991). "High-performance liquid chromatographic determination of loracarbef, a potential metabolite, cefaclor and cephalexin in human plasma, serum and urine." Journal of Chromatography B: Biomedical Sciences and Applications 567(1): 129-139.	Abstract
397	Koytchev, R., et al. (2004). "Studies on the bioequivalence of second generation cephalosporins: cefaclor capsules and suspension." Arzneimittelforschung 54(9a): 583-587.	Title
398	Kramer, W., et al. (1999). "Substrate specificity of the ileal and the hepatic Na+/bile acid cotransporters of the rabbit. I. Transport studies with membrane vesicles and cell lines expressing the cloned transporters." Journal of Lipid Research 40(9): 1604-1617.	Animal
399	Kramer, W., et al. (1997). "Modified bile acids as carriers for peptides and drugs." Journal of Controlled Release 46(1): 17-30.	Title
400	Kratochwil, N. A., et al. (2002). "Predicting plasma protein binding of drugs: a new approach." Biochem Pharmacol 64(9): 1355-1374.	Title

401	Kubbinga, M., et al. (2019). "The effect of chitosan on the bioaccessibility and intestinal permeability of acyclovir." European Journal of Pharmaceutics and Biopharmaceutics 136: 147-155.	Title
402	Kudo, Y., et al. (1989). "Carrier-mediated transport system for cephalexin in human placental brush-border membrane vesicles." Biochim Biophys Acta 978(2): 313-318.	Title
403	Kuemmerle, H. P. (1985). "New perspectives in oral antibiotic therapy: cefatrizine. Conclusions." Drugs Exp Clin Res 11(7): 461.	Title
404	Kulapina, O. I. and A. M. Vostrikova (2014). "[Rapid determination of cephalexin in biological media]." Antibiot Khimioter 59(1-2): 6-9.	Title
405	Kumar, K., et al. (2020). "Formulation, characterization and evaluation to establish the bioavailability of gastroretentive mucoadhesive dosage of atenolol in human subjects with possible in-vitro-in-vivo correlation." Iranian Journal of Pharmaceutical Sciences: 53-70.	Title
406	Kusuhara, H., et al. (2013). "In vivo characterization of interactions on transporters." Transporters in Drug Development: Discovery, Optimization, Clinical Study Regulation: 67-97.	Title
407	Kwon, H. (2003). Pharmacokinetic/pharmacodynamic modeling/simulation and novel gastric retention formulation, Oregon State University.	Title
408	La Rosa, F., et al. (1982). "Pharmacokinetics of cefadroxil after oral administration in humans." Antimicrob Agents Chemother 21(2): 320-322.	Title
409	Lai, X., et al. (2021). "An Open-Label, Randomized, 2-Way, Crossover Bioequivalence Study of Cefradine Capsules in Healthy Chinese Volunteers." Clin Pharmacol Drug Dev 10(12): 1478-1484.	Title
410	Landowski, C. P. (2005). Designing anticancer prodrugs for targeting drug transporters and activating enzymes, University of Michigan.	Title
411	Landowski, C. P., et al. (2003). "A fluorescent hPept1 transporter substrate for uptake screening." Pharm Res 20(11): 1738-1745.	Title
412	Landowski, C. P., et al. (2003). "Gene expression in the human intestine and correlation with oral valacyclovir pharmacokinetic parameters." Journal of Pharmacology Experimental Therapeutics 306(2): 778-786.	Title
413	LaPorte, B. and F. M. Musteata (2020). "Influence of Sampling Site and Fluid Flow on the Accuracy of Total Body Clearance Calculation." J Pharm Sci 109(6): 2079-2089.	Title
414	Law, M. R., et al. (1983). "Cefaclor and amoxycillin in the treatment of infective exacerbations of chronic bronchitis." Journal of antimicrobial chemotherapy 11(1): 83-88.	Title

415	Lawrence, X. Y. and G. L. Amidon (1998). "Saturable small intestinal drug absorption in humans: modeling and interpretation of cefatrizine data." European journal of pharmaceutics biopharmaceutics 45(2): 199-203.	Title
416	Lee, C. M. (2019). Physiologically-based Pharmacokinetic Modeling of Drugs with Transporter-mediated Disposition: Prediction of Vincristine and Amoxicillin Disposition in Children.	Title
417	Lee, C. S. and T. C. Marbury (1984). "Drug therapy in patients undergoing haemodialysis. Clinical pharmacokinetic considerations." Clin Pharmacokinet 9(1): 42-66.	Title
418	Lee, G. S., et al. (1980). "A comparison of cefaclor and ampicillin in the treatment of respiratory infection in elderly in-patients." Curr Med Res Opin 6(8): 564-568.	Title
419	Lee, J.-H., et al. (2022). "Oral pharmacokinetic profile and withdrawal time estimation for tylosin tartrate in the cultured olive flounder Paralichthys olivaceus." Aquaculture Reports 26: 101332.	Title
420	Lee, K. (2000). Novel mechanisms of absorptive and secretory transport of H2-antagonists across intestinal epithelium, The University of North Carolina at Chapel Hill.	Title
421	Lees, A. S., et al. (1993). "The pharmacokinetics, tissue penetration and in-vitro activity of loracarbef, a beta-lactam antibiotic of the carbacephem class." J Antimicrob Chemother 32(6): 853-859.	Title
422	Leigh, D. A. (1989). "Determination of serum and bone concentrations of cephradine and cefuroxime by HPLC in patients undergoing hip and knee joint replacement surgery." J Antimicrob Chemother 23(6): 877-883.	Title
423	Leitner, F., et al. (1979). "Bactericidal activity of cefadroxil, cephalexin, and cephradine in an in vitro pharmacokinetic model." J Antibiot (Tokyo) 32(7): 718-726.	Abstract
424	Lennernäs, H. (2014). "Human in vivo regional intestinal permeability: importance for pharmaceutical drug development." Molecular Pharmaceutics 11(1): 12-23.	Title
425	Leroy, A., et al. (1981). "[Pharmacokinetics of cefaclor in subjects with normal renal function and in uraemic patients (author's transl)]." Therapie 36(5): 533-540.	Title
426	Leroy, A., et al. (1982). "Pharmacokinetics of cefadroxil in patients with impaired renal function." J Antimicrob Chemother 10 Suppl B: 39-46.	Title
427	Levin, L. A., et al. (1996). "The spectrum of orbital aspergillosis: a clinicopathological review." Survey of Ophthalmology 41(2): 142-154.	Title

428	Levine, L. R. (1985). "Quantitative comparison of adverse reactions to cefaclor vs. amoxicillin in a surveillance study." Pediatric infectious disease 4(4): 358-361.	Title
429	Levison, M. E., et al. (1979). "In vitro activity and pharmacokinetics of cefaclor in normal volunteers and patients with renal failure." Postgrad Med J 55 Suppl 4: 12-16.	Title
430	Li, F., et al. (2006). "Transport of levovirin prodrugs in the human intestinal Caco-2 cell line." J Pharm Sci 95(6): 1318-1325.	Title
431	Li, J. and I. J. Hidalgo (1996). "Molecular modeling study of structural requirements for the oligopeptide transporter." J Drug Target 4(1): 9-17.	Title
432	Li, J. and I. J. Hidalgo (2019). The evolving role of the Caco-2 cell model to estimate intestinal absorption potential and elucidate transport mechanisms. Drug Discovery and Development, CRC Press: 333-357.	Title
433	Li, J., et al. (1998). "Structure-affinity relationships of Val-Val and Val-Val-Val stereoisomers with the apical oligopeptide transporter in human intestinal Caco-2 cells." J Drug Target 5(5): 317-327.	Title
434	Li, M., et al. (2009). "Effects of cranberry juice on pharmacokinetics of beta-lactam antibiotics following oral administration." Antimicrob Agents Chemother 53(7): 2725-2732.	Title
435	Li, Y. (2001). Mechanisms of region-dependent absorption of a weakly basic hiv protease inhibitor, indinavir: clinical ramifications and comparison with nelfinavir, University of Michigan.	Title
436	Liang, L. M., et al. (2020). "Diabetes downregulates peptide transporter 1 in the rat jejunum: possible involvement of cholate-induced FXR activation." Acta Pharmacol Sin 41(11): 1465-1475.	Animal
437	Lildholdt, T., et al. (1981). "Pharmacokinetics of cefaclor in chronic middle ear effusions." Ann Otol Rhinol Laryngol Suppl 90(3 Pt 3): 44-47.	Title
438	Lin, Y. T., et al. (2002). "Prophylactic antibiotics in cirrhotics with upper gastrointestinal hemorrhage: a prospective, controlled trial." Zhonghua yi xue za zhi [Chinese medical journal; Free China ed] 65(8): 365-371.	Title
439	Linnankoski, J., et al. (2008). "Passive oral drug absorption can be predicted more reliably by experimental than computational models—Fact or myth." European Journal of Pharmaceutical Sciences 34(2): 129-139.	Title
440	Lipsky, B. A., et al. (1999). "Sparfloxacin versus ciprofloxacin for the treatment of community-acquired, complicated skin and skin-structure infections." Clin Ther 21(4): 675-690.	Title

441	LiPuma, J. J. and T. L. Stull (1995). "ANTIBACTERIAL AGENTS IN PEDIATRICS." Infectious Disease Clinics of North America 9(3): 561-574.	Title
442	Litvak, A. S., et al. (1976). "Cefazolin and cephalexin levels in prostatic tissue and sera." Urology 7(5): 497-499.	Abstract
443	Liu, M., et al. (2016). "An LC-MS/MS method for simultaneous determination of cefprozil diastereomers in human plasma and its application for the bioequivalence study of two cefprozil tablets in healthy Chinese volunteers." Biomed Chromatogr 30(3): 288-293.	Title
444	Liu, Z., et al. (2011). "Uptake, transport and regulation of JBP485 by PEPT1 in vitro and in vivo." Peptides 32(4): 747-754.	Title
445	Lode, H. (2010). "Safety and Tolerability of Commonly Prescribed Oral Antibiotics for the Treatment of Respiratory Tract Infections." The American Journal of Medicine 123(4, Supplement): S26-S38.	Title
446	Lode, H., et al. (1973). "[Oral antibiotic therapy in gastrectomized patients]." Verh Dtsch Ges Inn Med 79: 837-839.	Title
447	Lode, H., et al. (1975). "Comparative pharmacokinetics and clinical experience with a new cephalosporin-derivative: cefazolin." Chemotherapy 21(1): 19-32.	Title
448	Lode, H., et al. (1979). "[Pharmacokinetics of cefaclor and initial therapeutical experience (author's transl)]." Infection 7 Suppl 6: 600-605.	Title
449	Lode, H., et al. (1980). "[Comparative pharmacokinetics of oral cephalosporins: cephalexin, cefaclor and cefadroxil (author's transl)]." Arzneimittelforschung 30(3): 505-509.	Full text
450	Lowther, J., et al. (1990). "Uptake of cephalosporins by human intestinal brush-border membrane vesicles." J Antimicrob Chemother 25(1): 183-184.	Title
451	Lu, H., et al. (2006). "Enantiomer–enantiomer interaction of ifosfamide in the rat." Xenobiotica 36(6): 535-549.	Animal
452	Lu, W., et al. (2008). "Modulation of brain delivery and copulation by intranasal apomorphine hydrochloride." Int J Pharm 349(1): 196-205.	Title
453	Lu, W.-Y., et al. (2021). "Application of marine natural products in drug research." Bioorg Med Chem 35: 116058.	Title
454	Ma, J., et al. (2010). "Effects of cefaclor on gastric emptying and cholecystokinin release in healthy humans." Regulatory peptides 159(1-3): 156-159.	Title
455	Ma, K. (2010). Role, Relevance and Regulation of PEPT1 in Peptide Intestinal Absorption.	Title
456	Madubala, P. (2020). Formulation and Evaluation of Floatting Drug Delivey System of Famotidine, PGP College of Pharmaceutical Science and Research Institute, Namakkal.	Title

457	Manda, P., et al. (2016). "Delivery of ziconotide to cerebrospinal fluid via intranasal pathway for the treatment of chronic pain." Journal of Controlled Release 224: 69-76.	Title
458	Mansmann, H. C. (1980). "THE EVALUATION, CONTROL, AND MODIFICATION OF CONTINUING ASTHMA." Clinics in Chest Medicine 1(3): 339-360.	Title
459	Marcon, M. J. and R. C. Bartlett (1983). "Laboratory evaluation of the serum dilution test in serious staphylococcal infection." Am J Clin Pathol 80(2): 176-181.	Title
460	Marino, A. M., et al. (1996). "Distribution of the dipeptide transporter system along the gastrointestinal tract of rats based on absorption of a stable and specific probe, SQ-29852." J Pharm Sci 85(3): 282-286.	Animal
461	Mariño, E. L. and A. Dominguez-Gil (1980). "Influence of dose on the pharmacokinetics of cefadroxil." Eur J Clin Pharmacol 18(6): 505-509.	Title
462	Mariño, E. L. and A. Dominguez-Gil (1981). "The pharmacokinetics of cefadroxil associated with probenecid." Int J Clin Pharmacol Ther Toxicol 19(11): 506-508.	Title
463	Mariño, E. L., et al. (1982). "Influence of dosage form and administration route on the pharmacokinetic parameters of cefadroxil." Int J Clin Pharmacol Ther Toxicol 20(2): 73-77.	Title
464	Marshall, B. C. and W. M. Samuelson (1998). "BASIC THERAPIES IN CYSTIC FIBROSIS: Does Standard Therapy Work?" Clinics in Chest Medicine 19(3): 487-504.	Title
465	Martea, M., et al. (1987). "Pharmacokinetics of cefradine, sulfamethoxazole and trimethoprim and their metabolites in a patient with peritonitis undergoing continuous ambulatory peritoneal dialysis." Pharm Weekbl Sci 9(2): 110-116.	Title
466	Martinez, M. N. and M. G. Papich (2009). "Factors Influencing the Gastric Residence of Dosage Forms in Dogs." J Pharm Sci 98(3): 844-860.	Animal
467	Marzo, A. and E. Heftmann (2002). "Enantioselective analytical methods in pharmacokinetics with specific reference to genetic polymorphic metabolism." Journal of Biochemical and Biophysical Methods 54(1): 57-70.	Title
468	Masahiro, N., et al. (1980). "Sustained urinary excretion of sulfamethizole following oral administration of enteric coated microcapsules in humans." Int J Pharm 4(4): 291-298.	Title
469	Mason, I. S. and M. Kietzmann (1999). "Cephalosporins - pharmacological basis of clinical use in veterinary dermatology." Vet Dermatol 10(3): 187-192.	Title

470	Mastrandrea, V., et al. (1985). "Pharmacokinetics of cefatrizine after oral administration in human volunteers." Int J Clin Pharmacol Res 5(5): 319-323.	Title
471	Matsumoto, S., et al. (1994). "Transcellular transport of oral cephalosporins in human intestinal epithelial cells, Caco-2: interaction with dipeptide transport systems in apical and basolateral membranes." J Pharmacol Exp Ther 270(2): 498-504.	Title
472	Matsunaga, T., et al. (1987). "A parallel comparative double blind study of cefixime with cefroxadine in the treatment of acute lacunar tonsillitis." Japanese journal of antibiotics 40(1): 25-54.	Title
473	Mattioli, F., et al. (2003). "Low plasma cefaclor levels in cystectomized bladder cancer patients with various types of urinary diversion." Eur J Clin Pharmacol 58(10): 715-716.	Title
474	Mazzei, T. and P. Dentico (2000). "The pharmacokinetics of oral cephalosporins." Clinical Microbiology and Infection 6: 53-54.	Abstract
475	Mazzei, T., et al. (2000). "New insight into the clinical pharmacokinetics of cefaclor: tissue penetration." J Chemother 12(1): 53-62.	Title
476	McCracken, G. H., Jr., et al. (1978). "Pharmacologic evaluation of orally administered antibiotics in infants and children: effect of feeding on bioavailability." Pediatrics 62(5): 738-743.	Title
477	Meimeti, E., et al. (2020). "Ointments containing Ceratothoa oestroides extract: evaluation of their healing potential in the treatment of diabetic foot ulcers." Wound repair and regeneration 28(2): 234-241.	Title
478	Meir, D. B. and P. M. Livne (2004). "Is prophylactic antimicrobial treatment necessary after hypospadias repair?" Journal of urology 171(6 Pt 2): 2621-2622.	Title
479	Menardi, G. and J. P. Guggenbichler (1984). "Bioavailability of oral antibiotics in children with short-bowel syndrome." J Pediatr Surg 19(1): 84-86.	Title
480	Mendelson, J., et al. (1979). "Effect of single and multidose cephradine prophylaxis on infectious morbidity of vaginal hysterectomy." Obstetrics and gynecology 53(1): 31-35.	Title
481	Meng, F., et al. (2005). "Sensitive liquid chromatography–tandem mass spectrometry method for the determination of cefixime in human plasma: Application to a pharmacokinetic study." Journal of Chromatography B 819(2): 277-282.	Title
482	Mesina, P., et al. (1984). "[Assay of blood and urinary levels of 2 different solutions of cephalexin for oral administration]." G Batteriol Virol Immunol 77(7-12): 137-144.	Abstract
483	Meunier, V., et al. (1995). "The human intestinal epithelial cell line Caco-2; pharmacological and pharmacokinetic applications." Cell biology toxicology 11: 187-194.	Title

484	Meyers, B. R. (2000). "Cefaclor revisited." Clin Ther 22(2): 154-166.	Title
485	Miao, L., et al. (2020). "Using a physiologically based pharmacokinetic absorption model to establish dissolution bioequivalence safe space for oseltamivir in adult and pediatric populations." The AAPS Journal 22: 1-10.	Title
486	Middlehurst, R. J., et al. (1989). "Cephradine penetration of mandibular bone." J Oral Maxillofac Surg 47(7): 672-673.	Title
487	Midtvedt, T. (1992). Penicillins, cephalosporins and tetracyclines. Side Effects of Drugs Annual. M. N. G. Dukes and J. K. Aronson, Elsevier. 16: 262-272.	Title
488	Minn, F. L., et al. (1976). "Cephradine excretion in humans with pH-altered urine." J Clin Pharmacol 16(4): 171-173.	Title
489	Miret, S., et al. (2004). "Comparison of in vitro models for the prediction of compound absorption across the human intestinal mucosa." J Biomol Screen 9(7): 598-606.	Title
490	Miyazaki, K., et al. (1983). "Determination of ampicillin, amoxicillin, cephalexin, and cephradine in plasma by high-performance liquid chromatography using fluorometric detection." J Chromatogr 276(2): 478-482.	Title
491	Moatti, N., et al. (1978). "[Human lung tissue and serum concentrations of cefradin (author's transl)]." Pathol Biol (Paris) 26(9-10): 577-580.	Title
492	Modr, Z., et al. (1982). "[Pharmacokinetics of cephradine]." Cesk Farm 31(5): 153-158.	Title
493	Mogabgab, W. J. (1976). "Parenteral use of cephradine in the treatment of lower respiratory tract infections: results of two studies." Curr Ther Res Clin Exp 20(3): 241-253.	Title
494	Molokhia, A. M. (1984). "Effect of storage on the bioavailability of cephalexin from its capsules." Res Commun Chem Pathol Pharmacol 45(2): 219-224.	Abstract
495	Moore, K., et al. (2019). "Double blind, placebo-controlled trial of antibiotics in women with refractory detrusor overactivity: effect on urge incontinence and quality of life." Neurourology and urodynamics 38: S120-S121.	Title
496	MORENO, L., et al. (2009). FOOD SAFETY AND DRUG RESIDUES.	Title
497	Morihana, T., et al. (1980). "[Fundamental and clinical studies of sustained release cephalexin (L-Keflex) in the field of oral surgery (author's transl)]." Jpn J Antibiot 33(4): 514-521.	Abstract
498	Morimoto, K., et al. (2011). "Effect of Milk on the Pharmacokinetics of Oseltamivir in Healthy Volunteers." J Pharm Sci 100(9): 3854-3861.	Title

499	Morrison, R. A., et al. (1996). "Suitability of enalapril as a probe of the dipeptide transporter system: in vitro and in vivo studies." Pharm Res 13(7): 1078-1082.	Title
500	Morse, D. R., et al. (1990). "A comparison of erythromycin and cefadroxil in the prevention of flare-ups from asymptomatic teeth with pulpal necrosis and associated periapical pathosis." Oral surgery, oral medicine, and oral pathology 69(5): 619-630.	Title
501	Motohiro, T., et al. (1992). "[Pharmacokinetic and clinical studies on cefprozil granules in the pediatric field]." Jpn J Antibiot 45(12): 1700-1735.	Title
502	Motohiro, T., et al. (1992). "Clinical studies on cefprozil granules in pediatric skin soft tissues infections." Japanese journal of antibiotics 45(12): 1684-1699.	Title
503	Motohiro, T., et al. (1989). "[Pharmacokinetic and clinical studies on cefpodoxime proxetil dry syrup in the field of pediatrics]." Jpn J Antibiot 42(7): 1629-1666.	Title
504	Motohiro, T., et al. (1988). "[Pharmacokinetic and clinical studies of sultamicillin granule in the pediatric field]." Jpn J Antibiot 41(12): 1980-2000.	Title
505	Motohiro, T., et al. (1981). "[Absorption, excretion and clinical trials of cefroxadine in the field of pediatrics (author's transl)]." Jpn J Antibiot 34(12): 1703-1731.	Title
506	Motohiro, T., et al. (1983). "[Clinical studies on cefadroxil in the field of pediatrics]." Jpn J Antibiot 36(1): 93-102.	Title
507	Motohiro, T., et al. (1989). "[Pharmacokinetic and clinical studies of cefteram pivoxil granule in the pediatric field]." Jpn J Antibiot 42(9): 2023-2061.	Title
508	Muijsers, R. B. R. and B. Jarvis (2002). "Moxifloxacin: in uncomplicated skin and skin structure infections." Drugs 62(6): 967-973.	Title
509	Muranushi, N., et al. (1994). "Characteristics of ceftibuten uptake into Caco-2 cells." Pharm Res 11(12): 1761-1765.	Title
510	Mustafa, M. M. and G. H. McCracken (1989). "Antimicrobial Agents in Pediatrics." Infectious Disease Clinics of North America 3(3): 491-506.	Title
511	Muth, P., et al. (1996). "Improved high-performance liquid chromatographic determination of amoxicillin in human plasma by means of column switching." J Chromatogr A 729(1-2): 259-266.	Title
512	Naber, K. and T. Ahrens (1980). "Dynamics of intial antibacterial effects in the treatment of bacteriuria." Urological research 8(4): 231.	Title
513	Naber, K. and W. Kaldewey (1979). "Comparative study of cefaclor versus amoxicillin in urinary tract infections (author's transl)." Infection 7 Suppl 6: 617-621.	Title

514	Naber, K. G. and T. Ahrens (1982). "Cefroxadine and cephalexin in urinary tract infections. Kinetics of germ elimination." Fortschritte der Medizin 100(39): 1827-1831.	Abstract
515	Nadai, M., et al. (2006). "Effect of chitosan on gastrointestinal absorption of water-insoluble drugs following oral administration in rats." Biological Pharmaceutical Bulletin 29(9): 1941-1946.	Animal
516	Nagpal, V. and R. Saha "INTRANASAL DELIVERY-OPPORTUNITIES FOR SYSTEMIC AND BRAIN TARGETING." Drug delivery and translational research.	Title
517	Nahata, M. C., et al. (1983). "Decreased absorption of cefaclor in short bowel syndrome." Drug Intell Clin Pharm 17(3): 201-202.	Title
518	Nahata, M. C., et al. (1990). "Cefadroxil kinetics and dynamics in a pediatric patient with acute osteomyelitis." Chemotherapy 36(6): 392-395.	Title
519	Najib, N., et al. (1987). "High performance liquid chromatographic analysis of cephalexin in serum and urine." Journal of clinical pharmacy therapeutics 12(6): 419-426.	Abstract
520	Najjar, T. A., et al. (2009). "Mechanism and implication of cephalosporin penetration into oropharyngeal mucosa." J Infect Chemother 15(2): 70-74.	Abstract
521	Nakamura, K., et al. (2021). "Pharmacologic therapy for engraftment arrhythmia induced by transplantation of human cardiomyocytes." Stem Cell Reports 16(10): 2473-2487.	Title
522	Nakashima, S., et al. (2022). "Effect of Excessive Serotonin on Pharmacokinetics of Cephalexin after Oral Administration: Studies with Serotonin-Excessive Model Rats." Pharm Res 39(9): 2163-2178.	Animal
523	Nakazawa, S., et al. (1981). "[Clinical studies with cefroxadine dry syrup in the field of pediatrics (author's transl)]." Jpn J Antibiot 34(12): 1588-1594.	Title
524	Nasrollahzadeh, M., et al. (2022). "Drug in adhesive transdermal patch containing antibiotic-loaded solid lipid nanoparticles." J Biosci Bioeng 134(5): 471-476.	Title
525	Nation, R. L., et al. (2014). "Pharmacokinetics and pharmacodynamics of colistin." Fundamentals of Antimicrobial Pharmacokinetics Pharmacodynamics: 351-380.	Title
526	Naumann, P. (1975). "[Cephalosporin antibiotics from microbiologic viewpoint. A comparison of antibacterial and pharmacokinetic properties]." Int J Clin Pharmacol Biopharm 11(2): 85-92.	Title

527	Naumann, P. and E. Reintjens (1974). "Antibacterial activity and pharmacokinetic behavior of cefazolin as compared with five other cephalosporin antibiotics." Infection 2(1): 19-24.	Title
528	Nct (2005). "The Impact of Treating Staphylococcus Aureus Infection and Colonization on the Clinical Severity of Atopic Dermatitis." https://clinicaltrials.gov/show/NCT00179959.	Title
529	Nct (2006). "Pediatric Fingertip Injuries: are Antibiotics Required?" https://clinicaltrials.gov/show/NCT00300092.	Title
530	Nct (2011). "Generic Formulations of Commonly-used Oral Drugs in Saudi Arabia: interchangeability & Post-marketing Quality." https://clinicaltrials.gov/show/NCT01344070.	Title
531	Nct (2011). "The Placebo Effect May Involve Modulating Drug Bioavailability." https://clinicaltrials.gov/show/NCT01501747.	Title
532	Nct (2011). "Safety and Efficacy Study of Ceftaroline Versus a Comparator in Pediatric Subjects With Complicated Skin Infections." https://clinicaltrials.gov/show/NCT01400867.	Title
533	Nct (2012). "Use of a Single Dose of Oral Prednisone in the Treatment of Cellulitis." https://clinicaltrials.gov/show/NCT01671423.	Title
534	Nct (2013). "Bioequivalence Study of Cephalexin Suspension 125." https://clinicaltrials.gov/show/NCT01767532.	Abstract
535	Nct (2013). "Bioequivalence Study of Cephalexin Suspension 250." https://clinicaltrials.gov/show/NCT01767571.	Abstract
536	Nct (2013). "Bioequivalence Study of Cephalexin Tablets 1g." https://clinicaltrials.gov/show/NCT01767584.	Abstract
537	Nct (2013). "Therapeutic Equivalence Between Branded and Generic WARFArin Tablets in Brazil." https://clinicaltrials.gov/show/NCT02017197.	Title
538	Nct (2014). "Anti-TNFα Use During Elective Foot and Ankle Surgery in Patients With Rheumatoid Arthritis." https://clinicaltrials.gov/show/NCT02242474.	Title
539	Nct (2014). "To Assess the Effect of AZD1722 on the Pharmacokinetics of Cefadroxil in Healthy Volunteers." https://clinicaltrials.gov/show/NCT02140281.	Title
540	Nct (2015). "A Bioequivalence Study of Cefadroxil From Duricef 1 gm F.C.T (GSK) and Biodroxil 1 gm F.C.T (Novartis Pharma)." https://clinicaltrials.gov/show/NCT02479867.	Title
541	Nct (2017). "Impact of Antibiotic Treatment Following Implantation of Cardiac Electronic Device on Patient's Outcome." https://clinicaltrials.gov/show/NCT03148444.	Title
542	Nct (2019). "Cefadroxil and Cephalexin Drug Levels and Dosing in Pediatric Musculoskeletal Infections." https://clinicaltrials.gov/show/NCT03802552.	Title

543	Nct (2022). "3-day IV Antibiotic Treatment Versus 3-day IV Followed by 7-day Oral Antibiotic Treatment for AP in Children." https://clinicaltrials.gov/show/NCT05544565.	Title
544	Nct (2023). "Single Dose Aminoglycosides for Acute Uncomplicated Cystitis in the Emergency Department Setting." https://clinicaltrials.gov/show/NCT05702762.	Title
545	Nelson, J. D., et al. (1981). "Concentrations of antimicrobial agents in middle ear fluid, saliva and tears." International journal of pediatric otorhinolaryngology 3(4): 327-334.	Title
546	Nelson, J. D., et al. (1978). "Oral antibiotic therapy for skeletal infections of children. I. Antibiotic concentrations in suppurative synovial fluid." Journal of pediatrics 92(1): 131-134.	Title
547	Neuhaus, F. C. and W. P. Hammes (1981). "Inhibition of cell wall biosynthesis by analogues of alanine." Pharmacology & Therapeutics 14(3): 265-319.	Title
548	Nguyen, H. M. and C. J. Graber (2020). "A Critical Review of Cephalexin and Cefadroxil for the Treatment of Acute Uncomplicated Lower Urinary Tract Infection in the Era of "Bad Bugs, Few Drugs"." Int J Antimicrob Agents 56(4): 106085.	Animal
549	Nicklin, P. L., et al. (1996). "Uptake and transport of the ACE-inhibitor ceronapril (SQ 29852) by monolayers of human intestinal absorptive (Caco-2) cells in vitro." Int J Pharm 140(2): 175-183.	Title
550	Nies, A. T., et al. (2016). "Structure and function of multidrug and toxin extrusion proteins (MATEs) and their relevance to drug therapy and personalized medicine." Arch Toxicol 90(7): 1555-1584.	Title
551	Nies, A. T., et al. (2012). "Multidrug and toxin extrusion proteins as transporters of antimicrobial drugs." Expert Opin Drug Metab Toxicol 8(12): 1565-1577.	Title
552	Nies, B. A. (1989). "Comparative activity of cefixime and cefaclor in an in vitro model simulating human pharmacokinetics." Eur J Clin Microbiol Infect Dis 8(6): 558-561.	Title
553	Nieto, M. J., et al. (1983). "Elimination of cefroxadine (CGP-9000) from patients undergoing dialysis." Eur J Clin Pharmacol 24(1): 109-112.	Title
554	Nightingale, C. (1980). "Pharmacokinetics of the oral cephalosporins in adults." J Int Med Res 8(Suppl 1): 2-8.	Abstract
555	Nightingale, C. H., et al. (1975). "Pharmacokinetics and clinical use of cephalosporin antibiotics." J Pharm Sci 64(12): 1899-1926.	Abstract
556	Nishimura, H., et al. (1969). "[Experimental evaluation of cephalexin (Lilly), a new cephalosporin C antibiotic. 2. Biological assay, absorption and excretion]." Saishin Igaku 24(9): 1983-1989.	Abstract

557	Nishimura, T., et al. (1981). "[Laboratory and clinical studies of cefroxadine (author's transl)]." Jpn J Antibiot 34(12): 1634-1646.	Title
558	Nishizawa, K., et al. (2019). "Changes of drug pharmacokinetics mediated by downregulation of kidney organic cation transporters Mate1 and Oct2 in a rat model of hyperuricemia." 14(4): e0214862.	Animal
559	Nix, D. E., et al. (1997). "Comparative pharmacokinetics of oral ceftibuten, cefixime, cefaclor, and cefuroxime axetil in healthy volunteers." Pharmacotherapy 17(1): 121-125.	Title
560	Nolen, T. (1994). "Comparative studies of cefprozil in the management of skin and soft-tissue infections." Eur J Clin Microbiol Infect Dis 13(10): 866-871.	Title
561	Nordt, S. P., et al. (1999). "Anaphylactic reaction to dermal exposure to cephalexin." Am J Emerg Med 17(5): 492-493.	Abstract
562	Nuñez-Vergara, L. J., et al. (1991). "Drug-acetaldehyde interactions during ethanol metabolism in vitro." Alcohol Alcohol 26(2): 139-146.	Title
563	Nungu, K. S., et al. (1995). "Bone and wound fluid concentrations of cephalosporins. Oral cefadroxil and parenteral cefuroxime compared in 52 patients with a trochanteric fracture." Acta Orthop Scand 66(2): 161-165.	Title
564	Ocheltree, S. M. (2005). The role and relevance of proton-coupled oligopeptide transporters in multiple organ systems: Implications for the regional and systemic disposition of peptides/mimetics, University of Michigan.	Title
565	Oguma, T., et al. (1991). "Pharmacokinetic analysis of the effects of different foods on absorption of cefaclor." Antimicrob Agents Chemother 35(9): 1729-1735.	Title
566	Ohkawa, M., et al. (1981). "Pharmacokinetics of cefroxadine in healthy volunteers and patients with impaired renal function." Chemotherapy 27(3): 149-154.	Title
567	Ohnhaus, E. E., et al. (1981). "Estimation of the biliary excretion of different cephalosporins utilizing retrograde cholangio-pancreatography (ERCP)." Endoscopy 13(1): 31-32.	Title
568	Okamura, M., et al. (2003). "Inhibitory effect of zinc on PEPT1-mediated transport of glycylsarcosine and beta-lactam antibiotics in human intestinal cell line Caco-2." Pharm Res 20(9): 1389-1393.	Title
569	Oliveira, C. H., et al. (2000). "Comparative bioavailability of two cefadroxil formulations in healthy human volunteers after a single-dose administration." Biopharm Drug Dispos 21(6): 243-247.	Title
570	Orsolini, P. (1970). "Tissue distribution and serum levels of cephalexin in man." Postgrad Med J: Suppl:13-16.	Abstract

571	Otoom, S., et al. (2004). "Comparative bioavailability of two cefadroxil products using serum and urine data in healthy human volunteers." Clin Exp Pharmacol Physiol 31(7): 433-437.	Title
572	Oulianova, N., et al. (2007). "Human oral drugs absorption is correlated to their in vitro uptake by brush border membrane vesicles." Int J Pharm 336(1): 115-121.	Title
573	Ovalle, A., et al. (2000). "Prospective, randomized, comparative study of the efficacy, safety and cost of cefuroxime versus cephradine in acute pyelonephritis during pregnancy." Revista medica de Chile 128(7): 749-757.	Title
574	Owens, D. R., et al. (1975). The Cephalosporin Group of Antibiotics. Advances in Pharmacology. S. Garattini, A. Goldin, F. Hawking, I. J. Kopin and R. J. Schnitzer, Academic Press. 13: 83-172.	Title
575	Padoin, C., et al. (1998). "Analysis of the pharmacokinetic interaction between cephalexin and quinapril by a nonlinear mixed-effect model." Antimicrob Agents Chemother 42(6): 1463-1469.	Abstract
576	Paixão, P., et al. (2012). "Prediction of the human oral bioavailability by using in vitro and in silico drug related parameters in a physiologically based absorption model." Int J Pharm 429(1): 84-98.	Title
577	Pallin, D. J., et al. (2013). "Clinical trial: comparative effectiveness of cephalexin plus trimethoprim-sulfamethoxazole versus cephalexin alone for treatment of uncomplicated cellulitis: a randomized controlled trial." Clinical infectious diseases 56(12): 1754-1762.	Title
578	Pan, X., et al. (2003). "Altered diurnal rhythm of intestinal peptide transporter by fasting and its effects on the pharmacokinetics of ceftibuten." Journal of Pharmacology Experimental Therapeutics 307(2): 626-632.	Title
579	Pankey, G. A. (1989). "Review of tissue penetration and clinical efficacy of enoxacin in skin and skin structure infections and in osteomyelitis." Clin Pharmacokinet 16 Suppl 1: 46-51.	Title
580	Papantoniou, N., et al. (2007). "Pharmacokinetics of oral cefatrizine in pregnant and non-pregnant women with reference to fetal distribution." Fetal Diagn Ther 22(2): 100-106.	Title
581	Papich, M. G. (2014). "Pharmacokinetic–pharmacodynamic (PK–PD) modeling and the rational selection of dosage regimes for the prudent use of antimicrobial drugs." Veterinary Microbiology 171(3): 480-486.	Title
582	Parish, L. C., et al. (1987). "Cefuroxime axetil in the treatment of cutaneous infections." International journal of dermatology 26(6): 389-393.	Title
583	Parish, L. C., et al. (2000). "Moxifloxacin versus cephalexin in the treatment of uncomplicated skin infections." Int J Clin Pract 54(8): 497-503.	Abstract

584	Parish, L. C. and J. A. Witkowski (1991). "Cephalosporin therapy in dermatologic practice." Clinics in Dermatology 9(4): 459-469.	Title
585	Parsons, R. L., et al. (1975). "The absorption of antibiotics in adult patients with coeliac disease." J Antimicrob Chemother 1(1): 39-50.	Title
586	Parsons, R. L. and G. M. Paddock (1975). "Absorption of two antibacterial drugs, cephalexin and co-trimoxazole, in malabsorption syndromes." J Antimicrob Chemother 1(3 Suppl): 59-67.	Title
587	Patel, H., et al. (2017). "Review of pharmacokinetic data of different drug classes in Goto-Kakizaki rats, a non-obese model for Type 2 diabetes mellitus: case studies and perspectives." European journal of drug metabolism pharmacokinetics 42: 173-182.	Animal
588	Paton, T. W., et al. (1985). "Drug therapy in patients undergoing peritoneal dialysis. Clinical pharmacokinetic considerations." Clin Pharmacokinet 10(5): 404-425.	Title
589	Paupe, J., et al. (1992). "Clinical trials on pediatric lower-respiratory-tract infection: results and comments with cefetamet pivoxil." Chemotherapy 38 Suppl 2: 29-32.	Title
590	Pedersen, S. S. (1989). "Clinical efficacy of ciprofloxacin in lower respiratory tract infections." Scand J Infect Dis Suppl 60: 89-97.	Title
591	Pennick, M. (2010). "Absorption of lisdexamfetamine dimesylate and its enzymatic conversion to d-amphetamine." Neuropsychiatr Dis Treat 6: 317-327.	Title
592	Perbellini, A., et al. (1975). "[Clinical experiences with the use of a new antibiotic in inflammatory diseases of surgical interest]." Minerva Chir 30(9): 520-531.	Title
593	Pérez-Bendito, D., et al. (1996). "Advances in drug analysis by kinetic methods." J Pharm Biomed Anal 14(8): 917-930.	Title
594	Perry, C. M. and L. J. Scott (2004). "Cefdinir: a review of its use in the management of mild-to-moderate bacterial infections." Drugs 64(13): 1433-1464.	Title
595	Peters, H. J. and G. Ludwig (1973). "[Antibiotic therapy of urinary tract infections]." Med Klin 68(26): 888-894.	Title
596	Pfeffer, M., et al. (1983). "Human intravenous pharmacokinetics and absolute oral bioavailability of cefatrizine." Antimicrob Agents Chemother 24(6): 915-920.	Title
597	Pfeffer, M., et al. (1977). "Comparative human oral clinical pharmacology of cefadroxil, cephalexin, and cephradine." Antimicrob Agents Chemother 11(2): 331-338.	Title
598	Pfeifer, H. J., et al. (1979). "Effects of three antibiotics on theophylline kinetics." Clin Pharmacol Ther 26(1): 36-40.	Title
599	Nine healthy subjects received single doses of intravenous aminophylline (5 mg/kg) on four occasions separated by at least 1 wk. One trial was a control; the other three were performed during administration of therapeutic doses (250 mg every 6 hr) of tetracycline,	Title

	erythromycin, and cephalexin. Theophylline concentrations in multiple plasma samples drawn during 24 hr after each dose were determined by high-pressure liquid chromatography. Mean kinetic variables for theophylline during control, tetracycline, erythromycin, and cephalexin trials, respectively, were: volume of distribution, 0.57, 0.61, 0.63, and 0.62 l/kg; elimination half-life (t1/2), 7.9, 9.2, 10.3, and 8.6 hr; total clearance, 0.90, 0.84, 0.78, and 0.89 ml/min/kg. In no case did differences between treatment conditions approach significance. Thus there is no consistent evidence of a kinetic interaction between theophylline and three commonly prescribed antibiotics.	
600	Phan, D. D., et al. (2003). "Intra- and interindividual variabilities of valacyclovir oral bioavailability and effect of coadministration of an hPEPT1 inhibitor." Antimicrob Agents Chemother 47(7): 2351-2353.	Title
601	Philipson, A., et al. (1987). "Comparison of the pharmacokinetics of cephradine and cefazolin in pregnant and non-pregnant women." Clin Pharmacokinet 12(2): 136-144.	Title
602	Piotrovskij, V. K., et al. (1994). "Modeling of the saturable time-constrained amoxicillin absorption in humans." 11: 1346-1351.	Title
603	Plöger, G. F., et al. (2020). "Biowaiver Monographs for Immediate Release Solid Oral Dosage Forms: Cephalexin Monohydrate." J Pharm Sci 109(6): 1846-1862.	Title
604	Polubiec, A., et al. (1988). "Bacterial infections of the lower urinary tract treated with ciprofloxacin or cefalexin--a comparative study." Infection 16 Suppl 1: S62-64.	Title
605	Popowicz, N. D., et al. (2018). "A rapid, LC-MS/MS assay for quantification of piperacillin and tazobactam in human plasma and pleural fluid; application to a clinical pharmacokinetic study." Journal of Chromatography B 1081-1082: 58-66.	Title
606	Posada Henao, M. M. (2012). Importance of PEPT1 in the Absorption, Tissue Distribution and Disposition of Cefadroxil.	Title
607	Posada, M. M. and D. E. Smith (2013). "In vivo absorption and disposition of cefadroxil after escalating oral doses in wild-type and PepT1 knockout mice." Pharm Res 30: 2931-2939.	Animal
608	Posada, M. M. and D. E. Smith (2013). "Relevance of PepT1 in the intestinal permeability and oral absorption of cefadroxil." Pharm Res 30: 1017-1025.	Title
609	Pradhan, S. (2020). Dose adjustment based on renal function, University of Otago.	Title
610	Prandota, J. (1988). "Clinical pharmacology of antibiotics and other drugs in cystic fibrosis." Drugs 35(5): 542-578.	Title
611	Putnam, W. S., et al. (2002). "Comparison of bidirectional cephalexin transport across MDCK and caco-2 cell monolayers: interactions with peptide transporters." Pharm Res 19(1): 27-33.	Title

612	Putnam, W. S., et al. (2002). "Functional characterization of monocarboxylic acid, large neutral amino acid, bile acid and peptide transporters, and P-glycoprotein in MDCK and Caco-2 cells." J Pharm Sci 91(12): 2622-2635.	Title
613	Qian, S., et al. (2014). "Development, characterization and application of in situ gel systems for intranasal delivery of tacrine." Int J Pharm 468(1): 272-282.	Title
614	Quenzer, R. U. and D. R. P. Guay (1995). "Antimicrobial management strategies for patients with community-acquired respiratory tract infections." Current Therapeutic Research 56(5): 466-477.	Title
615	Quevedo, A., et al. (1993). "Cefetamet pivoxil in otitis media." ORL; journal for oto-rhino-laryngology and its related specialties 55(2): 93-96.	Title
616	Quigley, J. M., et al. (1994). "The synthesis, hydrolysis kinetics and lipophilicity of O-acyl esters of propranolol." Int J Pharm 101(1): 145-163.	Title
617	Quintiliani, R. (1996). "Cefixime: a pharmacoeconomic perspective." Current Therapeutic Research 57(12): 892-912.	Title
618	Raeissi, S. D., et al. (1999). "The role of an alpha-amino group on H+ -dependent transepithelial transport of cephalosporins in Caco-2 cells." J Pharm Pharmacol 51(1): 35-40.	Title
619	Rahal, J. J. and M. S. Simberkoff (1978). "Adverse reactions to anti-infective agents." Disease-a-Month 25(1): 1-67.	Title
620	Rahim, N., et al. (2016). "Comparative bioavailability and pharmacokinetic study of Cefadroxil capsules in male healthy volunteers of Pakistan." Pak J Pharm Sci 29(2): 453-459.	Title
621	Rahim, S. A., et al. (2017). "Influence of calcium carbonate and sodium carbonate gassing agents on pentoxifylline floating tablets properties." Powder Technology 322: 65-74.	Title
622	Rakic, A., et al. (2007). "High-performance liquid chromatographic method for the determination of moclobemide and its two major metabolites in human plasma." J Pharm Biomed Anal 43(4): 1416-1422.	Title
623	Ranaldi, G., et al. (1994). "D-cycloserine uses an active transport mechanism in the human intestinal cell line Caco 2." Antimicrob Agents Chemother 38(6): 1239-1245.	Title
624	Randolph, M. F. (1988). "Clinical comparison of once-daily cefadroxil and thrice-daily cefaclor in the treatment of streptococcal pharyngitis." Chemotherapy 34(6): 512-518.	Title
625	Ranganath, S. H., et al. (2010). "The use of submicron/nanoscale PLGA implants to deliver paclitaxel with enhanced pharmacokinetics and therapeutic efficacy in intracranial glioblastoma in mice." Biomaterials 31(19): 5199-5207.	Animal

626	Rattie, E. S., et al. (1976). "Pharmacokinetic interpretation of cephradine levels in serum after intravenous and extravascular administration in humans." Antimicrob Agents Chemother 10(2): 283-287.	Title
627	Rauchegger, H., et al. (1989). "[Rational dosing intervals in streptococcal infections of the pharynx]." Wien Klin Wochenschr 101(7): 230-233.	Title
628	Reboli, A. C. and V. E. Del Bene (1988). "Oral Antibiotic Therapy of Dermatologic Conditions." Dermatologic Clinics 6(4): 497-520.	Title
629	Redrobe, S. (2000). "Treatment of avian liver disease." Seminars in Avian and Exotic Pet Medicine 9(3): 136-145.	Title
630	Reed, B. D., et al. (1991). "Treatment of beta-hemolytic streptococcal pharyngitis with cefaclor or penicillin. Efficacy and interaction with beta-lactamase-producing organisms in the pharynx." Journal of family practice 32(2): 138-144.	Title
631	Reed, M. D. and J. L. Blumer (1988). "Urologic Pharmacology in the Office Setting." Urologic Clinics of North America 15(4): 737-751.	Title
632	Regamey, C. and L. Humair (1971). "Pharmacokinetics of cephalexin in renal insufficiency." Postgrad Med J 47: Suppl:69-78.	Abstract
633	Rehm, S. J. and M. C. McHenry (1983). "Oral Antimicrobial Drugs." Medical Clinics of North America 67(1): 57-98.	Title
634	Reigner, B. G., et al. (1990). "Saturable rate of cefatrizine absorption after oral administration to humans." J Pharmacokinet Biopharm 18(1): 17-34.	Title
635	Retsema, J. (1999). "Susceptibility and resistance emergence studies with macrolides." Int J Antimicrob Agents 11: S15-S21.	Title
636	Reyes, L., et al. (2002). "Observer-blinded comparison of two nonopioid analgesics for postoperative pain in piglets." Pharmacology Biochemistry and Behavior 73(3): 521-528.	Title
637	Roberts, D. H., et al. (1981). "Pharmacokinetics of cephradine given intravenously with and without probenecid." Br J Clin Pharmacol 11(6): 561-564.	Title
638	Roberts, J. and N. Tumer (1988). "Age and diet effects on drug action." Pharmacology & Therapeutics 37(1): 111-149.	Title
639	Rodriguez, W. J., et al. (1979). "Cefaclor in the treatment of susceptible infections in infants and children." Postgrad Med J 55 Suppl 4: 35-38.	Title
640	Rooney, R., et al. (1988). "Serum concentrations of cephalosporins and the cell saver in cardiopulmonary bypass surgery." J Antimicrob Chemother 22(2): 266-268.	Title
641	Roseboom, P. H., et al. (2021). "Evidence in primates supporting the use of chemogenetics for the treatment of human refractory neuropsychiatric disorders." Molecular Therapy 29(12): 3484-3497.	Title

642	Rotschafer, J. C., et al. (1982). "Cefaclor pharmacokinetic parameters: serum concentrations determined by a new high-performance liquid chromatographic technique." Antimicrob Agents Chemother 21(1): 170-172.	Title
643	Rouan, M., et al. (1984). "Pharmacokinetics and dosage adjustment of cefotiam in renal impaired patients." Journal of antimicrobial chemotherapy 13(6): 611-618.	Title
644	Rouan, M. C., et al. (1983). "Systematic approach to the determination of cephalosporins in biological fluids by reversed-phase liquid chromatography." Journal of Chromatography B: Biomedical Sciences and Applications 275: 133-144.	Title
645	Rouan, M.-C., et al. (1985). "Pharmacokinetics of cefotiam in humans." Antimicrobial Agents Chemotherapy 27(2): 177-180.	Title
646	Roujeau, J.-C., et al. (1990). "Toxic epidermal necrolysis (Lyell syndrome)." Journal of the American Academy of Dermatology 23(6, Part 1): 1039-1058.	Title
647	Rylander, M., et al. (1979). "Penetration of cephradine and cefazolin into ulcers of patients suffering from peripheral arterial circulatory insufficiency." Scand J Infect Dis 11(4): 281-286.	Title
648	Sabella, C. and J. Goldfarb (1999). "Principles of selection and use of antimicrobial agents." Seminars in Pediatric Infectious Diseases 10(1): 3-13.	Title
649	Sader, H. S., et al. (2007). "Review of the spectrum and potency of orally administered cephalosporins and amoxicillin/clavulanate." Diagn Microbiol Infect Dis 57(3 Suppl): 5s-12s.	Title
650	Sadick, N. S. (2001). "SYSTEMIC ANTIBIOTIC AGENTS." Dermatologic Clinics 19(1): 1-21.	Title
651	Sain, B., et al. (2018). "Dalafloxacin-Antibacterial: a review." Int. J. Res 6: 91-101.	Title
652	Saito, A., et al. (2021). "Impact of Direction of Transport on the Evaluation of Inhibition Potencies of Multidrug and Toxin Extrusion Protein 1 Inhibitors." Drug Metab Dispos 49(2): 152-158.	Title
653	Saito, A., et al. (1977). "[Long-acting cefalexin preparation (S-6436): pharmacokinetics and clinical evaluation (author's transl)]." Kansenshogaku Zasshi 51(6): 303-311.	Abstract
654	Saito, H. and K. Inui (1993). "Dipeptide transporters in apical and basolateral membranes of the human intestinal cell line Caco-2." Am J Physiol 265(2 Pt 1): G289-294.	Title
655	Saito, Y., et al. (2019). "Effects of thylakoid-rich spinach extract on the pharmacokinetics of drugs in rats." Biological Pharmaceutical Bulletin 42(1): 103-109.	Animal

656	Saitoh, H., et al. (2002). "In vitro permeation of beta-lactam antibiotics across rat jejunum and its correlation with oral bioavailability in humans." Br J Clin Pharmacol 54(4): 445-448.	Title
657	Sakai, K., et al. (1985). "Clinical evaluation of S6472 (prolonged action preparation of cefaclor) in the treatment of skin and soft tissue infections. A double blind comparison of S6472 and cefaclor." Japanese journal of antibiotics 38(10): 2716-2734.	Title
658	Sakamoto, H., et al. (1985). "Pharmacokinetics of FK027 in rats and dogs." J Antibiot (Tokyo) 38(4): 496-504.	Animal
659	Sakamoto, H., et al. (1988). "Pharmacokinetics of FK482, a new orally active cephalosporin, in animals." J Antibiot (Tokyo) 41(12): 1896-1905.	Animal
660	Sakamoto, T., et al. (1985). "[Prolonged action preparation of cefaclor]." Jpn J Antibiot 38(3): 813-821.	Title
661	Sakane, T., et al. (1999). "Transnasal delivery of 5-fluorouracil to the brain in the rat." J Drug Target 7(3): 233-240.	Animal
662	Sala-Rabanal, M., et al. (2008). "Molecular mechanism of dipeptide and drug transport by the human renal H+/oligopeptide cotransporter hPEPT2." Am J Physiol Renal Physiol 294(6): F1422-1432.	Title
663	Sammeta, S. M., et al. (2009). "Dermal Drug Levels of Antibiotic (Cephalexin) Determined by Electroporation and Transcutaneous Sampling (ETS) Technique." J Pharm Sci 98(8): 2677-2685.	Title
664	Sánchez-Picó, A., et al. (1989). "Non-linear intestinal absorption kinetics of cefadroxil in the rat." J Pharm Pharmacol 41(3): 179-185.	Animal
665	Sandberg, T., et al. (1990). "Randomised double-blind study of norfloxacin and cefadroxil in the treatment of acute pyelonephritis." European journal of clinical microbiology & infectious diseases 9(5): 317-323.	Title
666	Sandberg, T., et al. (1985). "Cefadroxil once daily for three or seven days versus amoxycillin for seven days in uncomplicated urinary tract infections in women." Scand J Infect Dis 17(1): 83-87.	Title
667	Santella, P. J. and D. Henness (1982). "A review of the bioavailability of cefadroxil." J Antimicrob Chemother 10 Suppl B: 17-25.	Title
668	Santella, P. J., et al. (1978). "An overview of results of world-wide clinical trials with cefadroxil." J Int Med Res 6(6): 441-451.	Title
669	Sasaki, I., et al. (1990). "Clinical evaluation of cefuroxime axetil in acute dental infections. Double blind comparative study vs. cefaclor." Japanese journal of antibiotics 43(12): 2035-2068.	Title
670	Sasaki, J., et al. (1979). "[Absorption, excretion and pharmacokinetics of cefadroxil in human (author's transl)]." Jpn J Antibiot 32(12): 1372-1377.	Title
671	Satterwhite, J. H., et al. (1992). "Pharmacokinetics of cefaclor AF: effects of age, antacids and H2-receptor antagonists." Postgrad Med J 68 Suppl 3: S3-9.	Title

672	Scaglione, F., et al. (2003). "Measurement of cefaclor and amoxicillin-clavulanic acid levels in middle-ear fluid in patients with acute otitis media." Antimicrob Agents Chemother 47(9): 2987-2989.	Title
673	Schelleman, H., et al. (2008). "Warfarin with fluoroquinolones, sulfonamides, or azole antifungals: interactions and the risk of hospitalization for gastrointestinal bleeding." Clin Pharmacol Ther 84(5): 581-588.	Title
674	Schneider, H., et al. (1978). "Evaluation of an oral prolonged-release antibiotic formulation." J Pharm Sci 67(11): 1620-1622.	Title
675	Schneider, P., et al. (1982). "Antibacterial effects of cefroxadine, cephalexin and cephradine in a new in vitro pharmacokinetic model." J Antibiot (Tokyo) 35(7): 843-849.	Abstract
676	Schulze, J. D. R., et al. (2005). "Excipient effects on gastrointestinal transit and drug absorption in beagle dogs." Int J Pharm 300(1): 67-75.	Animal
677	Schwinghammer, T. L., et al. (1990). "Pharmacokinetics of cephradine administered intravenously and orally to young and elderly subjects." J Clin Pharmacol 30(10): 893-899.	Title
678	Segreti, J. and G. M. Trenholme (1986). "Antibiotics I: Beta-Lactam Antibiotics, the Tetracyclines, Chloramphenicol, Erythromycin, Clindamycin, Metronidazole, and the Quinolones." Clinics in Chest Medicine 7(3): 393-412.	Title
679	Selva Olid, A., et al. (2015). "Systemic antibiotics for treating diabetic foot infections." Cochrane Database of Systematic Reviews(9).	Title
680	Serra, C. H. S., et al. (2015). "Dissolution efficiency and bioequivalence study using urine data from healthy volunteers: a comparison between two tablet formulations of cephalexin." Brazilian journal of pharmaceutical sciences 51(2): 383-392.	Abstract
681	Seta, Y., et al. (1988). "Design and preparation of captopril sustained-release dosage forms and their biopharmaceutical properties." Int J Pharm 41(3): 245-254.	Title
682	Shanker, S. A., et al. (1983). "Effect of food on cefaclor bioavailability in children." Med J Aust 2(8): 372.	Title
683	Sharma, S. (2020). "Galantamine delivery for Alzheimer's disease." Sustainable Agriculture Reviews 43: Pharmaceutical Technology for Natural Products Delivery Vol. 1 Fundamentals Applications: 131-159.	Title
684	Sheen-Chen, S. M., et al. (1995). "Postoperative T-tube cholangiography: is routine antibiotic prophylaxis necessary? A prospective, controlled study." Archives of surgery (Chicago, Ill. : 1960) 130(1): 20-23.	Title
685	Shen, H. (2006). PEPT2 immunolocalization and its role in cefadroxil renal tubular reabsorption and brain penetration, University of Michigan.	Title

686	Shen, H., et al. (2007). "Impact of genetic knockout of PEPT2 on cefadroxil pharmacokinetics, renal tubular reabsorption, and brain penetration in mice." Drug metabolism disposition 35(7): 1209-1216.	Animal
687	Shibaki, H., et al. (1986). "A double blind comparative study on the efficacy of S6472, cefaclor and amoxicillin, in the treatment of bacterial pneumonia." Japanese journal of antibiotics 39(3): 853-886.	Title
688	Shimizu, R., et al. (2008). "Quantitative prediction of oral absorption of PEPT1 substrates based on in vitro uptake into Caco-2 cells." Int J Pharm 354(1-2): 104-110.	Title
689	Shimizu, T., et al. (2011). "PDZK1 regulates breast cancer resistance protein in small intestine." 39(11): 2148-2154.	Title
690	Shingaki, T., et al. (2011). "Nasal delivery of P-gp substrates to the brain through the nose–brain pathway." Drug metabolism pharmacokinetics 26(3): 248-255.	Title
691	Shingaki, T., et al. (2010). "Transnasal delivery of methotrexate to brain tumors in rats: a new strategy for brain tumor chemotherapy." Molecular Pharmaceutics 7(5): 1561-1568.	Animal
692	Shipkova, M. and D. Svinarov (2016). "LC–MS/MS as a tool for TDM services: Where are we?" Clinical Biochemistry 49(13): 1009-1023.	Title
693	Shoaib, M. H., et al. (2008). "Pharmacokinetic study of cephradine in Pakistani healthy male volunteers." Pak J Pharm Sci 21(4): 400-406.	Title
694	Shyu, W. C., et al. (1991). "Simultaneous high-performance liquid chromatographic analysis of cefprozil diastereomers in a pharmacokinetic study." Pharm Res 8(8): 992-996.	Title
695	Sides, G. D., et al. (1988). "A comprehensive review of the clinical pharmacology and pharmacokinetics of cefaclor." Clin Ther 11 Suppl A: 5-19.	Title
696	Simon, C. (1980). "[Pharmacokinetics of cefadroxil, a new oral cephalosporin (author's transl)]." Arzneimittelforschung 30(3): 502-504.	Title
697	Simon, C. and U. Gatzemeier (1979). "Serum and sputum levels of cefaclor." Postgrad Med J 55 Suppl 4: 30-34.	Title
698	Simon, C., et al. (1970). "[Pharmacokinetics of cephalexin in adults and children]." Dtsch Med Wochenschr 95(42): 2103-2108.	Abstract
699	Simone, C., et al. (1994). "Drug Transfer Across the Placenta: Considerations in Treatment and Research." Clinics in Perinatology 21(3): 463-481.	Title
700	Singhvi, S. M., et al. (1978). "Pharmacokinetics of cephalosporin antibiotics: protein-binding considerations." Chemotherapy 24(3): 121-133.	Title

701	Sinko, P. J., et al. (1993). "Mass balance approaches for estimating the intestinal absorption and metabolism of peptides and analogues: theoretical development and applications." Pharm Res 10(2): 271-275.	Title
702	Sjögren, E., et al. (2013). "In silico predictions of gastrointestinal drug absorption in pharmaceutical product development: application of the mechanistic absorption model GI-Sim." European Journal of Pharmaceutical Sciences 49(4): 679-698.	Title
703	Smith, L. G. and J. Sensakovic (1982). "Trimethoprim-Sulfamethoxazole." Medical Clinics of North America 66(1): 143-156.	Title
704	Smith, P., et al. (1994). SK&F 110679: comparison of absorption following oral or respiratory administration. Advances in Drug Delivery Systems, 6, Elsevier: 67-77.	Title
705	Smith, P. L., et al. (1994). "SK&F 110679: comparison of absorption following oral or respiratory administration." Journal of Controlled Release 28(1): 67-77.	Title
706	Snyder, N. J., et al. (1997). "Structure-activity relationship of carbacephalosporins and cephalosporins: antibacterial activity and interaction with the intestinal proton-dependent dipeptide transport carrier of Caco-2 cells." Antimicrob Agents Chemother 41(8): 1649-1657.	Title
707	Soback, S., et al. (1987). "Clinical pharmacokinetics of five oral cephalosporins in calves." Research in Veterinary Science 43(2): 166-172.	Animal
708	Sondheimer, J. H. and S. D. Migdal (1987). "Toxic Nephropathies." Critical Care Clinics 3(4): 883-907.	Title
709	Song, F., et al. (2017). "Species Differences in Human and Rodent PEPT2-Mediated Transport of Glycylsarcosine and Cefadroxil in Pichia Pastoris Transformants." Drug Metab Dispos 45(2): 130-136.	Title
710	Soranzo, M. L., et al. (1981). "[Clinical study on a new cephalosporin: CGP 9000 (cefroxadine)]." Minerva Med 72(13): 813-818.	Title
711	Sourgens, H., et al. (1997). "Pharmacokinetic profile of cefaclor." Int J Clin Pharmacol Ther 35(9): 374-380.	Title
712	Spreafico, P., et al. (1987). "Cesarean section: antibiotic prophylaxis with ceftezole." Chemioterapia 6(2 Suppl): 613-616.	Title
713	Spyker, D. A., et al. (1982). "Pharmacokinetics of cefaclor in renal failure: effects of multiple doses and hemodialysis." Antimicrob Agents Chemother 21(2): 278-281.	Title
714	Stamey, T. A. (1987). "Recurrent urinary tract infections in female patients: an overview of management and treatment." Rev Infect Dis 9 Suppl 2: S195-210.	Title
715	Statelova, M., et al. (2020). "On the design of food effect studies in adults for extrapolating oral drug absorption data to infants: an exploratory study highlighting the importance of infant food." The AAPS Journal 22: 1-11.	Title

716	Steffansen, B., et al. (1999). "Stability, metabolism and transport of D-Asp(OBzl)-Ala--a model prodrug with affinity for the oligopeptide transporter." Eur J Pharm Sci 8(1): 67-73.	Title
717	Stein, G. E., et al. (1997). "Pharmacodynamic activity of five oral cephalosporins against Haemophilus influenzae." Pharmacotherapy 17(2): 235-241.	Title
718	Stevens, D. L., et al. (1993). "Comparison of oral cefpodoxime proxetil and cefaclor in the treatment of skin and soft tissue infections." Diagn Microbiol Infect Dis 16(2): 123-129.	Title
719	Stevens, R. C. and J. H. Rodman (1998). "Pharmacokinetics of antimicrobial therapy." Seminars in Pediatric Infectious Diseases 9(4): 273-280.	Title
720	Stiefeld, S. M., et al. (1991). "Toxicities of Antimicrobial Agents Used to Treat Osteomyelitis." Orthopedic Clinics of North America 22(3): 439-465.	Title
721	Stillerman, M., et al. (1984). "Comparison between cephalexin two- and four-time per day regimens in group A streptococcal pharyngitis." Clinical pediatrics 23(6): 348-351.	Title
722	Stillhart, C., et al. (2020). "Impact of gastrointestinal physiology on drug absorption in special populations––An UNGAP review." European Journal of Pharmaceutical Sciences 147: 105280.	Title
723	Stone, J. W., et al. (1989). "Cefixime, in-vitro activity, pharmacokinetics and tissue penetration." J Antimicrob Chemother 23(2): 221-228.	Title
724	Strömberg, A., et al. (1987). "Concentrations of phenoxymethylpenicillin and cefadroxil in tonsillar tissue and tonsillar surface fluid." Eur J Clin Microbiol 6(5): 525-529.	Title
725	Strömberg, A., et al. (1988). "Five versus ten days treatment of group A streptococcal pharyngotonsillitis: a randomized controlled clinical trial with phenoxymethylpenicillin and cefadroxil." Scand J Infect Dis 20(1): 37-46.	Title
726	Subba Rao, S. D., et al. (1998). "A randomized, observer-blind trial of amoxycillin/clavulanate versus cefaclor in the treatment of children with acute otitis media. Augmentin 415 Study Group." J Chemother 10(6): 460-468.	Title
727	Sugawara, M., et al. (1991). "Transport characteristics of ceftibuten, cefixime and cephalexin across human jejunal brush-border membrane." J Pharm Pharmacol 43(12): 882-884.	Abstract
728	Sugawara, M., et al. (1992). "Transport characteristics of cephalosporin antibiotics across intestinal brush-border membrane in man, rat and rabbit." J Pharm Pharmacol 44(12): 968-972.	Animal

729	Sugiura, T., et al. (2008). "PDZK1 regulates two intestinal solute carriers (Slc15a1 and Slc22a5) in mice." Drug Metab Dispos 36(6): 1181-1188.	Animal
730	Sugiura, T., et al. (2010). "PDZK1 Regulates Organic Anion Transporting Polypeptide Oatpla in Mouse Small Intestine." Drug Metabolism Pharmacokinetics 25(6): 588-598.	Animal
731	Sugiura, T., et al. (2013). "Pharmacokinetic Modeling of Hepatocyte Growth Factor in Experimental Animals and Humans." J Pharm Sci 102(1): 237-249.	Title
732	Suleiman, M. S., et al. (1988). "A bioequivalence study of six brands of cephalexin." J Clin Pharm Ther 13(1): 65-72.	Abstract
733	Sun, B. W., et al. (2003). "Hormonal regulation of dipeptide transporter (PepT1) in Caco-2 cells with normal and anoxia/reoxygenation management." World J Gastroenterol 9(4): 808-812.	Title
734	Sun, L. N., et al. (2022). "LC-MS/MS methods for determination of unstable pivmecillinam and mecillinam in acidified human plasma: Application to a pharmacokinetic study." J Sep Sci 45(14): 2543-2554.	Title
735	Sun, Y., et al. (2018). "PEPT1-mediated prodrug strategy for oral delivery of peramivir." Asian journal of pharmaceutical sciences 13(6): 555-565.	Title
736	Sunakawa, K. and K. Shiba (1994). "[Reevaluation of current antimicrobials. Cefaclor]." Jpn J Antibiot 47(7): 853-860.	Title
737	Susanto, M. (2002). Investigating the role of P-glycoprotein on drugs exhibiting enhanced renal clearance in cystic fibrosis patients, University of California, San Francisco.	Title
738	Syposs, T., et al. (1986). "[Serum level of antibiotics in burn patients]." Zentralbl Chir 111(24): 1519-1525.	Title
739	Tagaya, E., et al. (2002). "Effect of a short course of clarithromycin therapy on sputum production in patients with chronic airway hypersecretion." Chest 122(1): 213-218.	Title
740	Tajana, A. (1991). "Stereoselective Pharmacokinetics." New Trends in Pharmacokinetics: 227-255.	Title
741	Takagi, T., et al. (2006). "A provisional biopharmaceutical classification of the top 200 oral drug products in the United States, Great Britain, Spain, and Japan." Molecular Pharmaceutics 3(6): 631-643.	Title
742	Takahashi, T., et al. (2010). "Effects of Acanthopanax senticosus HARMS extract on drug transport in human intestinal cell line Caco-2." J Nat Med 64(1): 55-62.	Title
743	Takeda, M., et al. (2002). "Interaction of human organic anion transporters with various cephalosporin antibiotics." Eur J Pharmacol 438(3): 137-142.	Title

744	Takimoto, M., et al. (1981). "[Clinical results of cefadroxil in children and pharmacokinetics of the drug (author's transl)]." Jpn J Antibiot 34(2): 140-142.	Title
745	Talluri, R. S. (2008). Stereoisomeric dipeptide prodrugs of acyclovir: A tool to improve cellular absorption, oralbioavailability and corneal permeation of Acyclovir in treatment of oral, genital and ocular herpes simplex viral infections, University of Missouri-Kansas City.	Title
746	Tamai, I., et al. (1995). "Functional expression of transporter for beta-lactam antibiotics and dipeptides in Xenopus laevis oocytes injected with messenger RNA from human, rat and rabbit small intestines." J Pharmacol Exp Ther 273(1): 26-31.	Animal
747	Tamassia, V., et al. (1979). "[Correlation between antibacterial activity of some cephalosporins and pharmacokinetic properties "in vitro" (author's transl)]." Quad Sclavo Diagn 15 Suppl 1: 785-792.	Title
748	Tamura, K., et al. (1996). "Metabolism, uptake, and transepithelial transport of the stereoisomers of Val-Val-Val in the human intestinal cell line, Caco-2." Pharm Res 13(11): 1663-1667.	Title
749	Tan, Z., et al. (2021). "Physiologically Based Pharmacokinetic Modeling of Cefadroxil in Mouse, Rat, and Human to Predict Concentration–Time Profile at Infected Tissue." Frontiers in pharmacology: 3630.	Animal
750	Tanaka, Y., et al. (2021). "Intraluminal Behavior of Various Transporter Substrates in the Rat Gastrointestinal Tract." Journal of Pharmacy Pharmaceutical Sciences 24: 563-570.	Animal
751	Tanimura, H., et al. (2003). "Study of the absorption of cefcapene pivoxil in patients with infectious disease and soft stool or diarrhea." Journal of Infection and Chemotherapy 9(1): 75-82.	Title
752	Tanrisever, B. and P. J. Santella (1986). "Cefadroxil. A review of its antibacterial, pharmacokinetic and therapeutic properties in comparison with cephalexin and cephradine." Drugs 32 Suppl 3: 1-16.	Title
753	Tao, W., et al. (2018). "Intestinal absorption and activation of decitabine amino acid ester prodrugs mediated by peptide transporter PEPT1 and enterocyte enzymes." Int J Pharm 541(1-2): 64-71.	Title
754	Tateno, M., et al. (1985). "[Absorption and excretion studies of S6472 (sustained release preparations of cefaclor)]." Jpn J Antibiot 38(3): 834-848.	Title
755	Tauchnitz, C. (1976). "[The cephalosporins and their clinical value]." Z Gesamte Inn Med 31(20): 828-834.	Title
756	Terada, T., et al. (2006). "Molecular cloning, functional characterization and tissue distribution of rat H+/organic cation antiporter MATE1." Pharm Res 23(8): 1696-1701.	Animal

757	Tetzlaff, T. R., et al. (1978). "Bioavailability of cephalexin in children: relationship to drug formulations and meals." J Pediatr 92(2): 292-294.	Abstract
758	Thompson, B. (2020). Mechanisms and Modeling of the Intestinal Absorption, Activation, and Systemic Availability of Gemcitabine and a Gemcitabine Prodrug for Oral Administration.	Title
759	Thornsberry, C. (1992). "Review of the in vitro antibacterial activity of cefprozil, a new oral cephalosporin." Clin Infect Dis 14 Suppl 2: S189-194; discussion S195-186.	Title
760	Thwaites, D. T., et al. (1994). "Substrate specificity of the di/tripeptide transporter in human intestinal epithelia (Caco-2): identification of substrates that undergo H(+)-coupled absorption." Br J Pharmacol 113(3): 1050-1056.	Title
761	Tian, C., et al. (2017). "Improving intestinal absorption and oral bioavailability of curcumin via taurocholic acid-modified nanostructured lipid carriers." International journal of nanomedicine 12: 7897.	Title
762	Todd, W. M. (1994). "Cefpodoxime proxetil: a comprehensive review." Int J Antimicrob Agents 4(1): 37-62.	Title
763	Tomatsu, K., et al. (1987). "In vitro and in vivo evaluations of BMY-28100, a new oral cephalosporin." J Antibiot (Tokyo) 40(8): 1175-1183.	Title
764	Tomić, Z., et al. (2016). "Do We Bury Antibacterials When Launching? Cefaclor Example." J Pharm Sci 105(3): 1295-1300.	Title
765	Touitou, E. and B. W. Barry (2006). Enhancement in drug delivery, CRC Press.	Title
766	Toyonaga, Y., et al. (1992). "[Bacteriological, pharmacokinetic and clinical studies of 5% and 10% granules of cefdinir in the pediatric field]." Jpn J Antibiot 45(1): 48-73.	Title
767	Toyonaga, Y., et al. (1989). "[Bacteriological, pharmacokinetic and clinical studies on cefteram pivoxil in the pediatric field]." Jpn J Antibiot 42(8): 1799-1814.	Title
768	Toyonaga, Y., et al. (1989). "[Bacteriological, pharmacokinetic and clinical studies on cefpodoxime proxetil in the pediatric field]." Jpn J Antibiot 42(7): 1519-1546.	Title
769	Toyonaga, Y., et al. (1986). "[Fundamental and clinical studies on cefixime (5% granules) in the pediatric field]." Jpn J Antibiot 39(4): 1055-1075.	Title
770	Trabucchi, E., Jr., et al. (1977). "[Absorption and elimination of a new cephalosporin]." Clin Ter 81(4): 299-307.	Title
771	Trachtman, H., et al. (2008). "Pilot Study of Mycophenolate Mofetil for Treatment of Kidney Disease due to Congenital Urinary Tract Disorders in Children." American Journal of Kidney Diseases 52(4): 706-715.	Title

772	Tschanz, C., et al. (1996). Interactions Between Drugs and Nutrients. Advances in Pharmacology. J. T. August, M. W. Anders, F. Murad and J. T. Coyle, Academic Press. 35: 1-26.	Title
773	Tsui, K., et al. (2016). "Sequential intravenous high dose oral antibiotics in the treatment of osteoarticular infections in children: a randomized controlled trial." Open forum infectious diseases 3.	Title
774	Tsuji, A., et al. (1983). "Physiologically based pharmacokinetic model for β-lactam antibiotics I: Tissue distribution and elimination in rats." J Pharm Sci 72(11): 1239-1252.	Animal
775	Tucker, G. T. and M. S. Lennard (1990). "Enantiomer specific pharmacokinetics." Pharmacology & Therapeutics 45(3): 309-329.	Title
776	Turgeon, J., et al. (1989). "Improved high-performance liquid chromatographic assay for encainide and its metabolites in human body fluids." Journal of Chromatography B: Biomedical Sciences and Applications 490: 165-174.	Title
777	Turnidge, J. D. (2011). "Cefazolin and enterobacteriaceae: rationale for revised susceptibility testing breakpoints." Clin Infect Dis 52(7): 917-924.	Title
778	Turuvekere Vittala Murthy, N., et al. (2021). "Polyphenols against infectious diseases: Controlled release nano-formulations." European Journal of Pharmaceutics and Biopharmaceutics 161: 66-79.	Title
779	Tutunji, M., et al. (2001). "Bioequivalence evaluation of two brands of cefaclor 500 mg capsules: quantification of cefaclor using solid phase extraction technique." J Clin Pharm Ther 26(2): 149-153.	Title
780	Upton, R. A. (1991). "Pharmacokinetic interactions between theophylline and other medication (Part I)." Clin Pharmacokinet 20(1): 66-80.	Title
781	Ustariz-Peyret, C., et al. (1999). "Cephradin-plaga microspheres for sustained delivery to cattle." J Microencapsul 16(2): 181-194.	Animal
782	Valassis, I. N., et al. (1999). "Quantitative determination of cefepime in plasma and vitreous fluid by high-performance liquid chromatography." Journal of Chromatography B: Biomedical Sciences and Applications 721(2): 249-255.	Title
783	Valko, K., et al. (2003). "Fast Gradient HPLC Method to Determine Compounds Binding to Human Serum Albumin. Relationships with Octanol/Water and Immobilized Artificial Membrane Lipophilicity." J Pharm Sci 92(11): 2236-2248.	Title
784	van Crugten, J., et al. (1986). "Selectivity of the cimetidine-induced alterations in the renal handling of organic substrates in humans. Studies with anionic, cationic and zwitterionic drugs." J Pharmacol Exp Ther 236(2): 481-487.	Title
785	Van Den Berg, M. P., et al. (2003). "Hydroxocobalamin uptake into the cerebrospinal fluid after nasal and intravenous delivery in rats and humans." J Drug Target 11(6): 325-331.	Title

786	Ved, P. M. and K. Kim (2011). "Poly(ethylene oxide/propylene oxide) copolymer thermo-reversible gelling system for the enhancement of intranasal zidovudine delivery to the brain." Int J Pharm 411(1): 1-9.	Title
787	Verhaegen, J. and L. Verbist (1992). "Oral cephalosporins." Acta Clin Belg 47(6): 377-386.	Title
788	Vila Jato, J. L., et al. (1982). "[Oral bioavailability of cefadroxil: influence of food]." Arch Farmacol Toxicol 8(3): 171-178.	Title
789	Vinchurkar, K., et al. (2022). "Features and Facts of a Gastroretentive Drug Delivery System-A Review." Turkish Journal of Pharmaceutical Sciences 19(4).	Title
790	Vittorini, C., et al. (1981). "Controlled assessment of the concentrations of a new cephalosporin in bile and gallbladder." Arzneimittelforschung 31(7): 1163-1165.	Title
791	von Hattingberg, H. M. (1975). "[Special pharmacokinetic aspects in newborns and young infants]." Monatsschr Kinderheilkd (1902) 123(1): 23-26.	Title
792	Vukovich, R. A., et al. (1975). "Sex differences in the intramuscular absorption and bioavailability of cephradine." Clin Pharmacol Ther 18(2): 215-220.	Title
793	Vukovich, R. A., et al. (1975). "Effect of 2% procaine hydrochloride solution on the bioavailability of cephradine after intramuscular injection." Curr Ther Res Clin Exp 18(5): 711-719.	Title
794	Wagner, J. G. (1974). "Relevant Pharmacokinetics of Antimicrobial Drugs." Medical Clinics of North America 58(3): 479-492.	Title
795	Walker, J. R., et al. (2009). "Quantitative structure-property relationships modeling to predict in vitro and in vivo binding of drugs to the bile sequestrant, colesevelam (Welchol)." The Journal of Clinical Pharmacology 49(10): 1185-1195.	Title
796	Walsh, M. L. and C. C. Johnson (1991). "Update on Antimicrobial Agents." Nursing Clinics of North America 26(2): 341-360.	Title
797	Wang, F., et al. (2003). "Profiles of methotrexate in blood and CSF following intranasal and intravenous administration to rats." Int J Pharm 263(1): 1-7.	Animal
798	Wang, Q., et al. (2007). "Pharmacokinetics of Gastrodin in rat plasma and CSF after i.n. and i.v." Int J Pharm 341(1): 20-25.	Animal
799	Wang, Q., et al. (2020). "Exclusion of unsuitable CNS drug candidates based on their physicochemical properties and unbound fractions in biomatrices for brain microdialysis investigations." J Pharm Biomed Anal 178: 112946.	Title
800	Watanabe, K., et al. (2004). "Effects of progesterone and norethisterone on cephalexin uptake in the human intestinal cell line Caco-2." Biol Pharm Bull 27(4): 559-563.	Title

801	Watanabe, K., et al. (2004). "Effect of insulin on cephalexin uptake and transepithelial transport in the human intestinal cell line Caco-2." Eur J Pharm Sci 21(1): 87-95.	Title
802	Watanabe, K., et al. (2003). "Intestinal absorption of cephalexin in diabetes mellitus model rats." European Journal of Pharmaceutical Sciences 19(2): 91-98.	Animal
803	Watanabe, S., et al. (2010). "Reduced renal clearance of a zwitterionic substrate cephalexin in MATE1-deficient mice." Journal of Pharmacology Experimental Therapeutics 334(2): 651-656.	Animal
804	Webster, C. A., et al. (1996). "Comparative in-vitro activity of cefaclor against urinary tract isolates of Escherichia coli." J Antimicrob Chemother 38(1): 59-66.	Title
805	Weese, J. S., et al. (2019). "International Society for Companion Animal Infectious Diseases (ISCAID) guidelines for the diagnosis and management of bacterial urinary tract infections in dogs and cats." The Veterinary Journal 247: 8-25.	Animal
806	Welling, P. G., et al. (1979). "The pharmacokinetics of the oral cephalosporins cefaclor, cephradine and cephalexin." Int J Clin Pharmacol Biopharm 17(9): 397-400.	Abstract
807	Welling, P. G., et al. (1979). "Probenecid: an unexplained effect on cephalosporin pharmacology." Br J Clin Pharmacol 8(5): 491-495.	Title
808	Wenzler, E., et al. (2018). "Drug-Food Interactions." Drug Interactions in Infectious Diseases: Mechanisms Models of Drug Interactions: 87-162.	Title
809	Whelton, A. (1982). "Antibiotic Pharmacokinetics and Clinical Application in Renal Insufficiency." Medical Clinics of North America 66(1): 267-281.	Title
810	Wierzba, K., et al. (1984). "[Pharmacokinetics of drugs in the growing child]." Pediatr Pol 59(11): 955-963.	Title
811	Wijma, R. (2019). Pharmacokinetic Profiling of Fosfomycin and Nitrofurantoin to Optimize the Treatment of Uncomplicated Urinary Tract Infections.	Title
812	Wijma, R. A., et al. (2020). "An audit of nitrofurantoin use in three Australian hospitals." Infection, Disease & Health 25(2): 124-129.	Title
813	Williams, J. D., et al. (1976). "Bacteriology and pharmacokinetics of the new amidino penicillin-mecillinam." J Antimicrob Chemother 2(1): 61-69.	Title
814	Williams, K. J., et al. (1987). "Cefuroxime axetil in the treatment of uncomplicated UTI: a comparison with cefaclor and augmentin." Drugs Exp Clin Res 13(2): 95-99.	Title
815	Wilson, C. G. J. D. B. (2003). "Considerations for Orally Administered Medications." 547.	Title

816	Wilson, R. (1993). "Cefaclor." Int J Antimicrob Agents 2(3): 185-198.	Title
817	Wilson, R. C., et al. (2022). "Addition of probenecid to oral β-lactam antibiotics: a systematic review and meta-analysis." J Antimicrob Chemother 77(9): 2364-2372.	Title
818	Windorfer, A. and P. Bauer (1982). "Pharmacokinetics and clinical studies with cefadroxil in paediatrics." J Antimicrob Chemother 10 Suppl B: 85-91.	Title
819	Wise, R. (1990). "The pharmacokinetics of the oral cephalosporins--a review." J Antimicrob Chemother 26 Suppl E: 13-20.	Abstract
820	Wise, R. (1994). "Comparative microbiological activity and pharmacokinetics of cefprozil." Eur J Clin Microbiol Infect Dis 13(10): 839-845.	Title
821	Wong, A. K., et al. (2002). "Determination of transport in the Caco-2 cell assay of compounds varying in lipophilicity using LC-MS: enhanced transport of Leu-enkephalin analogues." Eur J Pharm Sci 16(3): 113-118.	Title
822	Wortman, P. D. (1993). "Bacterial infections of the skin." Current Problems in Dermatology 5(6): 197-224.	Title
823	Xerri, L., et al. (1986). "Ofloxacin: bactericidal effect in an in vitro pharmacokinetic model." Chemioterapia 5(2): 83-87.	Title
824	Xiao, Y. H., et al. (2004). "[Comparison of pharmacokinetics/pharmacodynamics of cefdinir, cefpodoxime proxetil and cefaclor against common bacteria of community acquired infections]." Zhonghua Yi Xue Za Zhi 84(22): 1867-1871.	Title
825	Xie, B., et al. (2019). "Tenacigenin B ester derivatives from Marsdenia tenacissima actively inhibited CYP3A4 and enhanced in vivo antitumor activity of paclitaxel." Journal of Ethnopharmacology 235: 309-319.	Title
826	Xie, Y. (2015). Role and Relevance of PEPT1 in Intestinal Absorption and Pharmacokinetics of 5-Aminolevulinic Acid.	Title
827	Xie, Y., et al. (2016). "Population pharmacokinetic modeling of cefadroxil renal transport in wild-type and Pept2 knockout mice." Xenobiotica 46(4): 342-349.	Animal
828	Yamada, T., et al. (2022). "Probability of target attainment of oral antimicrobials for Escherichia coli and Klebsiella pneumoniae based on Monte Carlo simulations." Diagn Microbiol Infect Dis 103(1): 115662.	Title
829	Yamamoto, S., et al. (2017). "Utility of Göttingen minipigs for prediction of human pharmacokinetic profiles after dermal drug application." Pharmaceutical Research 34: 2415-2424.	Title
830	Yamamoto, T., et al. (1977). "[Laboratory and clinical studies on cefatrizine (author's transl)]." Jpn J Antibiot 30(10): 763-769.	Title
831	Yamashita, F., et al. (2006). "Quantitative structure/activity relationship modelling of pharmacokinetic properties using genetic algorithm-combined partial least squares method." J Drug Target 14(7): 496-504.	Title

832	Yamashita, S., et al. (2000). "Optimized conditions for prediction of intestinal drug permeability using Caco-2 cells." European Journal of Pharmaceutical Sciences 10(3): 195-204.	Title
833	Yamazaki, K. and M. Kanaoka (2004). "Computational prediction of the plasma protein-binding percent of diverse pharmaceutical compounds." J Pharm Sci 93(6): 1480-1494.	Title
834	Yáñez, J. A., et al. (2011). "Flip-flop pharmacokinetics–delivering a reversal of disposition: challenges and opportunities during drug development." Therapeutic delivery 2(5): 643-672.	Title
835	Yang, B. (2012). Role of peptide transporter PEPT1 in the intestinal absorption and pharmacokinetics of the amino acid ester prodrug valacyclover, University of Michigan.	Title
836	Yang, B., et al. (2013). "Impact of peptide transporter 1 on the intestinal absorption and pharmacokinetics of valacyclovir after oral dose escalation in wild-type and PepT1 knockout mice." Drug metabolism disposition 41(10): 1867-1874.	Animal
837	Yang, B. and D. E. Smith (2013). "Significance of peptide transporter 1 in the intestinal permeability of valacyclovir in wild-type and PepT1 knockout mice." Drug metabolism disposition 41(3): 608-614.	Animal
838	Yang, B. and D. E. Smith (2017). "In silico absorption analysis of valacyclovir in wildtype and Pept1 knockout mice following oral dose escalation." Pharm Res 34: 2349-2361.	Animal
839	Yang, L. P. and S. J. Keam (2008). "Retapamulin: a review of its use in the management of impetigo and other uncomplicated superficial skin infections." Drugs 68(6): 855-873.	Title
840	Yang, L. P. and S. J. Keam (2008). "Spotlight on retapamulin in impetigo and other uncomplicated superficial skin infections." Am J Clin Dermatol 9(6): 411-413.	Title
841	Yang, Z., et al. (2005). "Microdialysis evaluation of the brain distribution of stavudine following intranasal and intravenous administration to rats." Journal of pharmaceutical sciences 94(7): 1577-1588.	Animal
842	Yen, T. E., et al. (2005). "Prediction of drug absorption based on immobilized artificial membrane (IAM) chromatography separation and calculated molecular descriptors." J Pharm Biomed Anal 38(3): 472-478.	Title
843	Yin, O. Q., et al. (2006). "Variability in renal clearance of substrates for renal transporters in chinese subjects." J Clin Pharmacol 46(2): 157-163.	Title
844	Yost, R. L. and H. Derendorf (1985). "Rapid chromatographic determination of cefotaxime and its metabolite in biological fluids." J Chromatogr 341(1): 131-138.	Title

845	Yu, D. and L. L. Bio (2022). "Shedding Light on Amoxicillin, Amoxicillin-clavulanate, and Cephalexin Dosing in Children from a Pharmacist's Perspective." J Pediatric Infect Dis Soc 11(12): 594-602.	Abstract
846	Yu, L. X. and G. L. Amidon (1998). "Saturable small intestinal drug absorption in humans: modeling and interpretation of cefatrizine data." Eur J Pharm Biopharm 45(2): 199-203.	Title
847	Yuan, Z., et al. (1995). "Sensitive assay for measuring amoxicillin in human plasma and middle ear fluid using solid-phase extraction and reversed-phase high-performance liquid chromatography." J Chromatogr B Biomed Appl 674(1): 93-99.	Title
848	Zaharenko, L. (2015). Perorāla antidiabētiska medikamenta metformīna efektivitātes un panesamības farmakoģenētika.	Language
849	Zaharenko, L. (2015). "Pharmacogenetics of efficiency and tolerance of the peroral antidiabetic drug metformin." Riga: University of Latvia.	Title
850	Zhang, E. Y., et al. (2002). "Modeling of active transport systems." Advanced Drug Delivery Reviews 54(3): 329-354.	Title
851	Zhang, J., et al. (2010). "Pharmacokinetic interaction between JBP485 and cephalexin in rats." Drug Metab Dispos 38(6): 930-938.	Animal
852	Zhang, M., et al. (2018). "Simultaneous Determination of Cefalexin, Cefazolin, Flucloxacillin, and Probenecid by Liquid Chromatography-Tandem Mass Spectrometry for Total and Unbound Concentrations in Human Plasma." Ther Drug Monit 40(6): 682-692.	Abstract
853	Zhang, S., et al. (2019). "Evaluating the bioequivalence of metronidazole tablets and analyzing the effect of in vitro dissolution on in vivo absorption based on PBPK modeling." Drug Development sIndustrial Pharmacy 45(10): 1646-1653.	Title
854	Zhang, Z., et al. (2013). "Intestinal mucosal permeability of children with cefaclor-associated serum sickness-like reactions." Eur J Pediatr 172(4): 537-543.	Title
855	Zhao, M., et al. (2016). "Animal models in the pharmacokinetic/pharmacodynamic evaluation of antimicrobial agents." Bioorg Med Chem 24(24): 6390-6400.	Animal
856	Zhao, X. L. and S. Deng (1995). "Pharmacokinetic studies of long acting cephalexin granules." The chinese journal of clinical pharmacology 11(1): 16-19.	Abstract
857	Zhu, L. (2006). Intestinal barriers to oral bioavailability: Saquinavir as a model compound, University of Illinois at Chicago, Health Sciences Center.	Title
858	Zhu, Q., et al. (2014). "Drug interaction studies reveal that simotinib upregulates intestinal absorption by increasing the paracellular permeability of intestinal epithelial cells." Drug Metab Pharmacokinet 29(4): 317-324.	Title

859	Zuckerman, J. M. and K. M. Kaye (1995). "THE NEWER MACROLIDES: Azithromycin and Clarithromycin." Infectious Disease Clinics of North America 9(3): 731-745.	Title
860	梁骏, et al. (2017). 头孢拉定在糖尿病和正常大鼠体内药动学比较. 36: 256-261.	Language

Supplementary Table S2: Quality assessment of included articles by Jadad scoring

	JADAD Questions						
**Sr. No.**	**Reference**	**Was the study described as randomized?**	**Was the method used to generate the sequence of randomization described and appropriate?**	**Was the study described as double blind?**	**Was the method of double blinding described and appropriate?**	**Was there a description of withdrawals and dropouts?**	**JADAD score**
1-	Liew et. al	1	1	0	0	1	3
2-	Liu R et. al	1	1	0	0	1	3
3-	Hassanzadeh et. al	1	1	1	1	0	4
4-	Bataineh et. al	1	1	0	0	0	2
5-	Ding et. al	1	1	0	0	0	2
6-	Lecailllom et. al	0	0	0	0	0	0
7-	Ginsburg et. al	1	0	0	0	0	1
8-	Spyker et. al	0	0	0	0	0	0
9-	Nakagawa et. al	0	0	0	0	0	0
10-	Nahata et. al	0	0	0	0	0	0
11-	Lode et. al	1	1	0	0	0	2
12-	Evert et. al	1	1	0	0	0	2
13-	Kelly et. al	1	1	0	0	0	2
14-	Akimoto et. al	0	0	0	0	0	0
15-	Barbhayia et. al	1	1	0	0	0	2
16-	Finkelstein et. al	1	1	0	0	0	2
17-	Ding Y et. al	1	1	0	0	0	2

18-	Mohamed S et. al	1	1	0	0	0	2
19-	Welling et. al	1	1	0	0	0	2
20-	Bunke et. al	1	1	0	0	0	2
21-	Liu R et. al	1	1	0	0	0	2
22-	Regamey et. al	0	0	0	0	0	0
23-	Autmizguine et. Al	1	1	0	0	0	2

Supplementary Table S3: Quality assessment of included articles by CACPK scoring

	**References**																						
**Questions**	1	2	3	4	5	6	7	8	9	10	11	12	13	14	15	16	17	18	19	20	21	22	23
Q1	Y	Y	Y	Y	Y	Y	Y	Y	Y	Y	Y	Y	Y	Y	Y	Y	Y	Y	Y	Y	Y	Y	Y
Q2	Y	Y	Y	Y	Y	Y	Y	Y	Y	Y	Y	Y	Y	Y	Y	Y	Y	Y	Y	Y	Y	Y	Y
Q3	Y	Y	Y	Y	Y	N	Y	N	Y	N	Y	Y	Y	N	Y	N	Y	Y	Y	Y	Y	N	N
Q4	Y	Y	Y	Y	Y	Y	Y	Y	Y	Y	Y	Y	Y	Y	Y	Y	Y	Y	Y	Y	Y	Y	Y
Q5	Y	Y	Y	Y	Y	Y	Y	Y	Y	Y	Y	Y	Y	Y	Y	Y	Y	Y	Y	Y	Y	Y	Y
Q6	Y	Y	N	N	Y	Y	Y	Y	N	Y	Y	Y	Y	N	Y	Y	Y	Y	N	N	Y	N	N
Q7	Y	N	Y	Y	Y	Y	Y	Y	Y	Y	Y	Y	Y	Y	Y	Y	Y	Y	Y	Y	Y	Y	Y
Q8	Y	Y	IDK	N	Y	N	IDK	IDK	N	Y	Y	N	Y	N	N	N	Y	Y	IDK	Y	Y	IDK	IDK
Q9	Y	Y	Y	Y	Y	Y	Y	Y	Y	Y	Y	Y	Y	Y	Y	Y	Y	Y	Y	Y	Y	Y	Y
Q10	Y	Y	Y	Y	Y	Y	Y	Y	Y	Y	Y	Y	Y	Y	Y	Y	Y	Y	Y	Y	Y	Y	Y
Q11	Y	Y	Y	Y	Y	Y	Y	Y	Y	Y	Y	Y	Y	Y	Y	Y	Y	Y	Y	Y	Y	Y	Y
Q12	Y	IDK	IDK	IDK	N	N	IDK	N	N	N	N	IDK	N	IDK	IDK	N	N	N	N	N	N	N	N
Q13	Y	Y	Y	Y	Y	Y	N	Y	N	Y	Y	Y	Y	Y	Y	Y	Y	Y	Y	Y	Y	Y	Y
Q14	Y	Y	IDK	N	Y	N	N	N	N	N	Y	Y	Y	N	N	N	N	N	N	Y	Y	N	N
Q15	Y	Y	IDK	N	Y	IDK	N	N	N	N	Y	Y	Y	N	N	N	Y	N	N	Y	Y	N	N
Q16	Y	Y	Y	Y	Y	Y	Y	Y	Y	Y	Y	Y	Y	Y	Y	Y	Y	Y	Y	Y	Y	Y	Y
Q17	N	N	N	N	N	N	N	N	N	Y	Y	Y	N	N	N	N	N	N	Y	Y	N	Y	Y

Q18	Y	Y	Y	Y	Y	Y	Y	Y	N	N	Y	Y	Y	IDK	Y	N	Y	Y	Y	Y	Y	N	Y
Q19	Y	Y	Y	Y	Y	Y	Y	Y	IDK	Y	Y	Y	IDK	Y	Y	IDK	Y	Y	Y	Y	Y	Y	Y
Q20	IDK	IDK	IDK	IDK	IDK	IDK	IDK	IDK	IDK	IDK	IDK	N	N	N	N	N	N	N	N	N	IDK	N	IDK
Q21	Y	Y	Y	Y	Y	Y	Y	Y	N	N	Y	Y	Y	IDK	Y	N	Y	Y	Y	Y	Y	N	Y
**DELPHI Score**	19	17	14	14	18	14	14	14	10	14	19	18	17	11	15	11	17	16	15	18	18	12	14

Y:Yes, N:No, IDK: I don't know

Supplementary Table S4: Quality assessment of included articles by CASP scoring

Reference	Was there a clear statement of the aims of the research?	Is a qualitative methodology appropriate?	Was the research design appropriate to address the aims of the research?	Are the study's theoretical underpinnings clear, concictent and conceptually coherent?	Was the recruitment strategy appropriate to the aims of the search?	Was the data collected in a way that addressed the research issue?	Has the relationship between researher and participants been adequately considered?	Have ethical issues been taken into consideration?	Was the data analysis sufficiently rigrous?	Is there a clear statement of findings?	CASP Score
Liew et. al	Y	Y	Y	Y	Y	Y	Y	Y	Y	Y	10
Liu R et. al	Y	Y	Y	Y	Y	Y	Y	Y	Y	Y	10
Hassanzadeh et. al	Y	Y	Y	Y	Y	Y	Y	Y	Y	Y	10
Bataineh et. al	Y	Y	Y	Y	Y	Y	CT	Y	Y	Y	9
Ding et. al	Y	Y	Y	Y	Y	Y	CT	Y	Y	Y	9
Lecaillom et. al	Y	N	N	Y	Y	Y	CT	N	Y	Y	6
Ginsburg et. al	Y	N	N	Y	Y	Y	N	Y	Y	Y	7
Spyker et. al	Y	N	N	Y	Y	Y	N	Y	Y	Y	7
Nakagawa et. al	Y	N	N	Y	N	Y	CT	N	Y	Y	5

Nahata et. al	Y	N	N	Y	Y	Y	N	Y	Y	Y	7
Lode et. al	Y	Y	Y	Y	Y	Y	CT	Y	Y	Y	9
Evert et. al	Y	Y	Y	Y	Y	Y	N	N	Y	Y	8
Kelly et. al	Y	Y	Y	Y	Y	Y	CT	Y	Y	Y	9
Akimoto et. al	Y	CT	CT	Y	Y	Y	CT	N	Y	Y	6
Barbhayia et. al	Y	Y	Y	Y	Y	Y	CT	Y	Y	Y	9
Finkelstein et. al	Y	Y	Y	Y	Y	Y	N	N	Y	Y	8
Ding Y et. al	Y	Y	Y	Y	Y	Y	Y	Y	Y	Y	10
Mohamed S et. al	Y	Y	Y	Y	Y	Y	CT	Y	Y	Y	9
Welling et. al	Y	Y	Y	Y	Y	Y	CT	Y	Y	Y	9
Bunke et. al	Y	Y	Y	Y	Y	Y	N	N	Y	Y	8
Liu R et. al	Y	Y	Y	Y	Y	Y	Y	Y	Y	Y	10
Regamey et. al	Y	CT	CT	Y	Y	Y	CT	CT	Y	Y	6
Autmizguine et. Al	Y	Y	N	Y	Y	Y	N	Y	Y	Y	8

Y:Yes, N:No, CT: Can't tell

Supplementary Table S5: Risk of bias assessment of included articles by Cochrane Collaboration tool

	Reference	Random sequence Generation (selection bias)	Allocaton concealment (selection bias)	Blinding of participant and researchers (performed bias)	Blinding of outcome assessment (detection bias)	Incomplete outcome data (attrition bias)	Selective reporting (reporting bias)	Other bias	Bias Score
1-	Liew et. al	LR	LR	HR	UR	LR	LR	LR	5
2-	Liu R et. al	LR	LR	UR	UR	LR	LR	LR	5
3-	Hassanzadeh et. al	LR	LR	LR	LR	LR	LR	LR	7
4-	Bataineh et. al	LR	LR	HR	UR	UR	LR	LR	4
5-	Ding et. al	LR	LR	HR	UR	UR	LR	LR	4
6-	Lecailllom et. al	HR	HR	HR	UR	LR	LR	LR	3
7-	Ginsburg et. al	LR	LR	HR	HR	UR	LR	LR	4
8-	Spyker et. al	HR	HR	HR	UR	LR	LR	LR	3
9-	Nakagawa et. al	HR	HR	HR	UR	LR	LR	LR	3
10-	Nahata et. al	HR	HR	HR	UR	LR	LR	LR	3
11-	Lode et. al	LR	LR	HR	UR	LR	LR	LR	5
12-	Evert et. al	LR	LR	HR	HR	UR	LR	LR	4
13-	Kelly et. al	LR	LR	HR	HR	UR	LR	LR	4
14-	Akimoto et. al	HR	HR	HR	UR	LR	LR	LR	3
15-	Barbhayia et. al	LR	LR	HR	UR	LR	LR	LR	5
16-	Finkelstein et. al	HR	HR	HR	UR	LR	LR	LR	3
17-	Ding Y et. al	LR	LR	HR	UR	LR	LR	LR	5
18-	Mohamed S et. al	LR	LR	HR	UR	UR	LR	LR	4
19-	Welling et. al	LR	LR	HR	HR	UR	LR	LR	4

20-	Bunke et. al	HR	HR	HR	UR	LR	LR	LR	3
21-	Liu R et. al	HR	HR	HR	UR	LR	LR	LR	3
22-	Regamey et. al	HR	HR	HR	UR	LR	LR	LR	3
23-	Autmizguine et. Al	LR	LR	HR	HR	UR	LR	LR	4

LR: Low risk, HR: High risk, UR: Unclear risk
